# Isolation of Ce(iv) centered polyoxoalkoxide sandwich-type complexes allows comparison of metal–oxygen bond covalency

**DOI:** 10.1039/d5sc06415e

**Published:** 2025-10-13

**Authors:** Dominic Shiels, Michele Pittalis, Nadeeshan Gunarathna, Adriana C. Berlfein, William W. Brennessel, Michael T. Ruggiero, Ellen M. Matson

**Affiliations:** a Department of Chemistry, University of Rochester Rochester NY 14627 USA shiels@ur.rochester.edu michael.ruggiero@rochester.edu matson@chem.rochester.edu

## Abstract

The new Ce^iii^ centered sandwich-type complex (TBA)_3_[Ce{W_4_O_13_(OMe)_4_MoNO}_2_] is reported. The redox properties of this molecule, and its all-molybdenum analogue, (TBA)_3_[Ce{Mo_5_O_13_(OMe)_4_NO}_2_], were investigated using cyclic voltammetry. The data reveals the presence of reversible Ce^iv^/Ce^iii^ redox couples at modest potentials. One electron oxidation of the complexes provides facile access to the corresponding Ce^iv^ derivatives, which were fully characterized. ^17^O NMR spectroscopy reveals that the chemical shifts of the oxygen nuclei directly bound to Ce^iv^ are much higher than the corresponding signals in isostructural, diamagnetic, Zr^iv^, Hf^iv^, or Th^iv^ centered complexes. Density functional theory (DFT) calculations indicate that the increase in chemical shift correlates with an increase in the covalency of the M^iv^–O bonds, illustrating that ^17^O NMR spectroscopy is a powerful experimental tool for interrogating the nature of metal oxygen bonding in diamagnetic complexes.

## Abstract 2

Ce(iv) centered polyoxoalkoxide sandwich-type complexes have been isolated. Combining ^17^O NMR spectroscopy and density functional theory calculations reveals a relationship between the observed chemical shift and M(IV)–O bond covalency.

## Graphical abstract

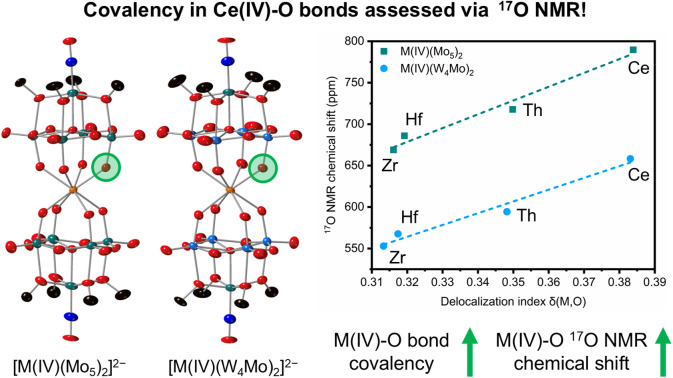

## Introduction

The manipulation of lanthanide (Ln) and actinide (An) redox states is exploited in separation processes, where the ability to selectively oxidize certain metal cations facilitates their isolation from complex mixtures.^1–3^ In this context, cerium is unique amongst the lanthanides due to its ability to access a [Xe]4f^0^ electronic configuration upon oxidation to the +4 oxidation state.^4,5^ Other high valent lanthanides are rarely observed, with only a handful of molecular complexes known.^6–11^ The ability to readily cycle between Ce^iii^ and Ce^iv^ has been exploited extensively in organic chemistry, with cerium complexes being applied as oxidants in a range of organic transformations, while cerium oxide has been used catalytic converters in petrol cars to support the oxidation of CO to CO_2_.^12–18^

Trivalent lanthanides are often considered as possessing localized, core-like, valence 4f orbitals and therefore are said to primarily form ionic bonds.^19,20^ However, recent work has shown that oxidation of Ce^iii^ to Ce^iv^ can “turn on” mixing of metal 4f orbitals with ligand orbitals, leading to the formation of bonds with a higher degree of covalent character.^21^ This phenomenon is well documented for [Ce^iii^Cl_6_]^3−^ and [Ce^iv^Cl_6_]^2−^, where Cl K-edge X-ray absorption spectroscopy (XAS) demonstrates a substantial increase in Ce–Cl orbital mixing upon oxidation of the lanthanide from Ce^iii^ to Ce^iv^.^20–22^ These results were compared to a series of [M^iv^Cl_6_]^2−^ (M = Ti, Zr, Hf, and U) and, interestingly, the 4f-orbital participation in the Ce–Cl bonds of [Ce^iv^Cl_6_]^2−^ was observed to be more than twice that of the 5f-orbital contribution to the corresponding U–Cl bonds of [U^iv^Cl_6_]^2−^. This was attributed to the lower energy of the Ce 4f- *vs.* U 5f-orbitals, implying that matching the energy of Cl 3p orbitals with the f-orbitals is a stronger contributor to the covalency of these types of bonds. A number of studies utilizing XAS experiments and/or computational calculations to assess the covalency of Ln–E/An–E bonds (E = C, N, O, S) exist.^4,23–35^ Nuclear magnetic resonance (NMR) spectroscopy has also proved useful in many studies where simulation of NMR spectra is often used as a tool to verify the accuracy of computational studies.^36–39^ Furthermore, the spin–orbit contributions (*σ*_SO_) to the nuclear shielding constant (*σ*) often provide valuable insights when assessing bond covalency.^40–43^

To carry out investigations of this type, isostructural An and Ln containing complexes are required to make like for like comparisons. In this context, polyoxometalates (POMs), anionic molecular metal oxide clusters typically based on W^vi^, Mo^vi^, or V^v^, have become popular ligands in lanthanide and actinide chemistry.^44–61^ While some studies with Ce-POM systems focus on catalytic^62,63^ and biological^64–67^ applications, many of the studies in this field limit analysis to solid state characterization. Indeed, despite the fact that a growing number of isostructural Ln-POM/An-POM systems are known, there are no studies which employ these complexes as frameworks for systematic comparison of Ln–O/An–O bonding.

Previously, our group utilized lacunary polyoxoalkoxide complexes, (TBA)_2_[Mo_5_O_13_(OMe)_4_NO][Na(MeOH)] (1-NaMo_5_) and (TBA)_2_[W_4_O_13_(OMe)_4_MoNO][Na(MeOH)] (1-NaW_4_Mo), for the synthesis of a series of M^iv^ centered sandwich-type assemblies with the general formula (TBA)_2_[M^iv^{M′_4_O_13_(OMe)_4_MoNO}_2_] (M^iv^ = Zr, Hf, Th, Np, U and M′ = Mo or W).^68–70^ Detailed structural analysis was performed by single crystal X-ray diffraction (SCXRD). Additionally, the solubility of these complexes renders solution phase analysis possible. Indeed, characterization of the (TBA)_2_[M^iv^{M′_4_O_13_(OMe)_4_MoNO}_2_] complexes by ^17^O NMR spectroscopy offers a direct spectroscopic handle for the M^iv^–O bond. Other groups have previously isolated several isostructural lanthanide centered complexes with the general formula (TBA)_3_[Ln^iii^{Mo_5_O_13_(OMe)_4_NO}_2_], where Ln^iii^ = Ce, Eu Tb, Dy, Ho, and Er. These were obtained either by treatment of 1-NaMo_5_ with an appropriate lanthanide salt in methanol^71^ or by treatment of (TBA)_4_[α-Mo_8_O_26_] with hydroxylamine, dicyclohexylcarbodiimide (DCC), and the appropriate lanthanide nitrate in methanol.^72^ The related complexes (TBA)_3_[Ln^iii^{Mo_5_O_13_(OMe)_4_NNC_6_H_4_-*p*-NO_2_}_2_] (where Ln^iii^ = Tb, Dy, Ho, Er, Yb, and Nd) have also been reported and their magnetic properties were investigated.^73^ Interestingly, no attempts were made to interrogate the electrochemistry of any of these lanthanide complexes and, consequently, Ce^iv^ centered complexes remain unknown.

Herein, we extend Proust and Villaneau's method for the synthesis of (TBA)_3_[Ce^iii^{Mo_5_O_13_(OMe)_4_NO}_2_] (2-Ce(Mo_5_)_2_) to access the tungsten containing analogue (TBA)_3_[Ce^iii^{W_4_O_13_(OMe)_4_MoNO}_2_] (2-Ce(W_4_Mo)_2_).^71^ Both complexes were fully characterized by ^1^H NMR spectroscopy, ^17^O NMR spectroscopy, electronic absorption spectroscopy, SCXRD and cyclic voltammetry. Importantly, the cyclic voltammograms (CVs) of 2-Ce(Mo_5_)_2_ and 2-Ce(W_4_Mo)_2_ show the presence of a reversible Ce^iv^/Ce^iii^ redox couple at *ca.* 0.3–0.4 V *vs.* Fc^+/0^. With this knowledge in hand, we carried out one electron oxidation to isolate the Ce^iv^ centered complexes (TBA)_2_[Ce^iv^{Mo_5_O_13_(OMe)_4_NO}_2_] (3-Ce(Mo_5_)_2_) and (TBA)_2_[Ce^iv^{W_4_O_13_(OMe)_4_MoNO}_2_] (3-Ce(W_4_Mo)_2_). Electronic absorption spectroscopy of the oxidized complexes reveals the presence of a new ligand-to-metal charge transfer (LMCT) process between the polyoxoalkoxo metalloligand and the empty 4f^0^ orbital of Ce^iv^, with this assignment supported by time-dependent density functional theory (TD-DFT) calculations. ^17^O NMR spectroscopy of the now diamagnetic 3-Ce(Mo_5_)_2_ and 3-Ce(W_4_Mo)_2_ complexes shows that the chemical shifts of the oxygen nuclei directly bound to Ce^iv^ are much higher than the corresponding signals in the other diamagnetic M^iv^ centered complexes we have reported.^68–70^ This increase in chemical shift correlates with an increase in metal orbital contribution to the M^iv^–O bonds and delocalization index (DI), both of which were obtained from DFT calculations and can be used as a measure for bond covalency. Collectively, these data show that ^17^O NMR chemical shift acts as an experimental handle for M–O bond covalency in diamagnetic polyoxometalate complexes.^33^

## Experimental

### General considerations

Air- and moisture-sensitive manipulations with all complexes were carried out using a standard high-vacuum line, Schlenk techniques, or an MBraun inert atmosphere drybox containing an atmosphere of purified dinitrogen. The MBraun glovebox was equipped with a cold well designed for freezing samples in liquid nitrogen, as well as a −35 °C freezer for cooling samples and crystallizations. Solvents for sensitive manipulations were dried and deoxygenated using literature procedures with a Seca solvent purification system or a glass contour solvent purification system (Pure Process Technology, LLC) and stored over activated 4 Å molecular sieves (Fisher Scientific) prior to use. Deuterated solvents were purchased from Cambridge Isotope Laboratories, dried with molecular sieves and degassed by three freeze–pump–thaw cycles. 40% ^17^O enriched H_2_O was purchased from CortecNet and used as received. (TBA)_4_[Mo_8_O_26_],^74^ (TBA)_2_[WO_4_],^75^ (TBA)_2_[Mo_5_O_13_(OMe)_4_NO][Na(MeOH)] (1-NaMo_5_)^76^ and, (TBA)_2_[W_4_O_13_(OMe)_4_MoNO][Na(MeOH)] (1-NaW_4_Mo)^68,71^ were synthesized according to literature procedures. The synthesis of 2-Ce(Mo_5_)_2_ and 2-Ce(W_4_Mo)_2_ was adapted from the previously reported procedure.^71^ Ce(OTf)_3_ was purchased from Strem Chemicals, while all other reagents were purchased from commercial sources (Fisher Scientific, VWR, and Sigma-Aldrich) and used without further purification.

### General procedure for the synthesis of (TBA)_3_[Ce^iii^{M_4_O_13_(OMe)_4_MoNO}_2_] (M = Mo, 2-Ce(Mo_5_)_2_, M = W, 2-Ce(W_4_Mo)_2_)

In a 15 mL pressure vessel, (TBA)_2_[M_4_O_13_(OMe)_4_MoNO][Na(MeOH)] (0.36 mmol, 2 eq.) was dissolved in MeOH (4 mL) forming a purple solution. Ce(OTf)_3_ (OTf = O_3_SCF_3_; 117 mg, 0.20 mmol, 1.1 eq.) was dissolved in MeOH (4 mL) and added slowly to the mixture. The pressure vessel was sealed, and the solution was heated at 50 °C for 2 hours with stirring. The hot reaction mixture was passed through a filter paper to remove any precipitate. The filtered reaction mixture was then cooled to −30 °C in a freezer and stored overnight, after which block-shaped crystals formed. The mother liquor was decanted, and the crystals were washed with cold MeOH (2 mL) and Et_2_O (10 mL × 2). The crystals were then dried under vacuum.

#### 2-Ce(Mo_5_)_2_

Red/violet solid (317 mg, 68% yield). ^1^H NMR (500 MHz, CD_3_CN): *δ* (ppm) 1.17 (t, 36H), 1.66 (m, 24H), –CH_2_ peak overlap with reference peak at 1.94, 3.34 (t, 24H), 3.84 (s, 24H). ^17^O NMR (67.8 MHz, CD_3_CN): *δ* (ppm) −41.6 (μ_5_–O), 479.7 (Mo–O–Mo), 879.8 (Mo=O), 886.8 (Ce–O–Mo). *λ*_max_ (MeCN) = 552 nm (*ε* = 144 mol^−1^ dm^3^ cm^−1^). Anal. Calcd. for C_56_H_132_N_5_Mo_10_O_36_Ce (mol. wt 2551.287 g mol^−1^): C, 26.36%; H, 5.22%; N, 2.75%. Found: C, 26.452%; H, 4.927%; N, 2.802%.

#### 2-Ce(W_4_Mo)_2_

Blue/purple solid (334 mg, 57% yield). ^1^H NMR (500 MHz, CD_3_CN): *δ* (ppm) 1.42 (t, 36H), 2.06 (m, 24H), 2.38 (m, 24H), 3.18 (s, 24H), 3.92 (t, 24H). ^17^O NMR (67.8 MHz, CD_3_CN): *δ* (ppm) −76.8 (μ_5_–O), 356.4 (W–O–W), 715.7 (W=O), 740.4 (Ce–O–W). *λ*_max_ (MeCN) = 564 nm (*ε* = 129 mol^−1^ dm^3^ cm^−1^). Anal. Calcd. for C_56_H_132_N_5_W_8_Mo_2_O_36_Ce (mol. wt 3254.407 g mol^−1^): C, 20.67%; H, 4.09%; N, 2.15%. Found: C, 20.580%; H, 3.815%; N, 2.146%.

### General procedure for the synthesis of (TBA)_2_[Ce^iv^{M_4_O_13_(OMe)_4_MoNO}_2_] (M = Mo, 3-Ce(Mo_5_)_2_, M = W, 3-Ce(W_4_Mo)_2_)

In a 20 mL scintillation vial, (TBA)_3_[Ce{M_4_O_13_(OMe)_4_MoNO}_2_] (0.039 mmol, 1 eq.) was dissolved in MeCN (2–3 ml) forming a homogenous solution. The solution was transferred to a vial containing solid [N(C_6_H_4_Br-4)_3_][SbCl_6_] (35 mg, 0.043 mmol, 1.1 eq.). The solution immediately turns dark orange/brown when using 2-Ce(Mo_5_)_2_ or dark yellow/brown when using 3-Ce(W_4_Mo)_2_. The mixture was stirred 15 minutes, and subsequently dried under vacuum leaving a dark residue. The residue was suspended in MeOH (5 mL) and the resultant suspension filtered through a bed of Celite (*ca.* 1 cm) and the eluent was discarded. The solid was washed with MeCN : Et_2_O (1 : 5, 2 mL) and then extracted with DCM until eluent ran colorless (*ca.* 5 mL). This solution was then dried under vacuum to give crude product.

#### 3-Ce(Mo_5_)_2_

Orange solid (70 mg, 78% yield). Dark orange block-shaped single crystals were obtained by vapor diffusion of Et_2_O into a saturated solution of the product in MeCN at room temperature. ^1^H NMR (500 MHz, CD_2_Cl_2_): *δ* (ppm) 1.12 (t, 24H), 1.58 (m, 16H), 1.83 (m, 16H), 3.34 (s, 24H), 4.73 (t, 24H). ^17^O NMR (67.8 MHz, CD_2_Cl_2_): *δ* (ppm) 16.3 (μ_5_–O), 534.9 (Mo–O–Mo), 789.7 (Ce–O–Mo), 925.1 (Mo=O). Anal. Calcd. for C_40_H_96_N_4_Mo_10_O_36_Ce.0.5 Et_2_O (mol. wt 2345.878 g mol^−1^): C, 21.50%; H, 4.34%; N, 2.39%. Found: C, 21.748%; H, 4.389%; N, 2.486%.

#### 3-Ce(W_4_Mo)_2_

Yellow solid (81 mg, 69% yield). Yellow block-shaped single crystals were obtained by vapor diffusion of Et_2_O into a saturated solution of the product in MeCN at room temperature. ^1^H NMR (500 MHz, CD_2_Cl_2_): *δ* (ppm) 1.12 (t, 24H), 1.59 (m, 16H), 1.85 (m, 16H), 3.37 (s, 24H), 4.87 (t, 24H). ^17^O NMR (67.8 MHz, CD_2_Cl_2_): *δ* (ppm) −12.8 (μ_5_–O), 395.8 (W–O–W), 660.8 (Ce–O–W), 755.9 (W=O). *λ*_max_ (MeCN) = 568 nm (*ε* ≈ 105 mol^−1^ dm^3^ cm^−1^). Anal. Calcd. for C_40_H_96_N_4_Mo_10_O_36_Ce.MeOH (mol. wt 3043.978 g mol^−1^): C, 16.18%; H, 3.31%; N, 1.84%. Found: C, 16.407%; H, 2.994%; N, 1.627%.

### Physical measurements

^1^H NMR spectra for all other compounds were recorded at room temperature on a 400 MHz Bruker AVANCE spectrometer, a 500 MHz Bruker AVANCE spectrometer, or a JEOL 500 spectrometer and locked on the signal of deuterated solvents. All chemical shifts are reported relative to tetramethylsilane using the chosen deuterated solvent as a standard. ^17^O NMR spectra were collected at room temperature on a JEOL 500 spectrometer or a 500 MHz Bruker AVANCE spectrometer (both at 67.8 MHz), with the spectrometer locked on the signal of the deuterated solvent and all chemical shifts given relative to an external standard of D_2_O. Cyclic voltammetry (CV) was performed using a three-electrode setup inside a glove box (MBraun UniLab, USA) using a Bio-Logic SP 150 potentiostat/galvanostat. The concentration of the cluster and the supporting electrolyte (TBAPF_6_) were kept at 1 mM and 100 mM respectively throughout all measurements. CVs were recorded using a 3 mm diameter glassy carbon working electrode (CH Instruments, USA), a Pt wire auxiliary electrode (CH Instruments, USA), and a Ag/Ag^+^ non-aqueous reference electrode with 0.01 M AgNO_3_ in 0.1 M TBAPF_6_ in acetonitrile (BASi, USA). Ferrocene was used as an internal standard after completion of the measurements, and potentials were referenced *versus* the Fc^+/0^ couple. Electronic absorption measurements were recorded at room temperature in anhydrous MeCN or DCM in sealed 1 cm quartz cuvettes using an Agilent Cary 6000i UV-vis-NIR spectrophotometer. Elemental analysis data were obtained from the Elemental Analysis Facility at the University of Rochester. Microanalysis samples were weighed with a PerkinElmer model AD6000 autobalance, and their compositions were determined with a PerkinElmer 2400 series II analyzer. Air-sensitive samples were handled in a VAC Atmospheres glovebox.

### X-ray crystallography

Crystals were placed onto a nylon loop and mounted on a Rigaku XtaLAB Synergy-S Dualflex diffractometer equipped with a HyPix-6000HE HPC area detector for data collection at 100.00(10) K. A preliminary set of cell constants and an orientation matrix were calculated from a small sampling of reflections.^77^ A short pre-experiment was run, with both CuK*α* and MoK*α* radiation, from which an optimal data collection strategy was determined. All data for the reported crystal structures were ultimately collected with CuK*α* radiation, as the results of the pre-experiments indicated that using MoK*α* radiation did not offer any significant improvement in structure quality, but greatly increased the collection time. After the intensity data were corrected for absorption, the final cell constants were calculated from the full dataset from the xyz centroids of the strong reflections.^77^ The structures were solved using SHELXT and refined using SHELXL.^78,79^ Full-matrix least squares/difference Fourier cycles were performed to assign the non-hydrogen atoms. All non-hydrogen atoms were first refined isotropically, followed by using anisotropic displacement parameters. All hydrogen atoms were placed in ideal positions and refined as riding atoms with relative isotropic displacement parameters. Additional refinement details are given in the SI, Section S5.

### Computational methods

All DFT calculations were performed in the gas phase using ORCA 6.0.0.^80^ The initial geometry for all complexes studied was taken from SCXRD structures. Solvent molecules and counter cations were removed. Relevant bond distances were compared to the experimental crystal structure, resulting in absolute average errors of 0.65% for Ce^iv^–O bonds and −1.33% for M^vi^–O bonds. All calculations presented here were performed using the hybrid exchange-correlation functional PBE0,^81^ and scalar relativistic corrections were added with the ZORA method as developed in ORCA.^82,83^ An all-electron basis set, SARC/ZORA-def2TZVP was used for all the atoms.^84^ Fine tolerances on energy (10^−11^), density matrix (10^−8^), and integrations were used. To account for solvent effects, the electronic structure was then obtained with the conductor-like polarizable continuum model (CPCM) using acetonitrile as the solvent.

Natural bond order (NBO) analyses were performed within the ORCA interface.^85^ From the NBO, it was possible to obtain the metal atom contribution to the natural localized molecular orbitals (NLMOs). The obtained NBO Lewis structure and shape of the relevant orbitals were checked against various DFT functionals and basis sets (see SI) and were found to be consistent across methods. QTAIM analysis was performed using the Basin Analysis function within the program Multiwfn.^86,87^ Using the electron density, it was possible to extract the Delocalization Index (DI) value for the studied systems.

## Results & discussion

### Synthesis and characterization of Ce^iii^ complexes

**Fig. 1:**
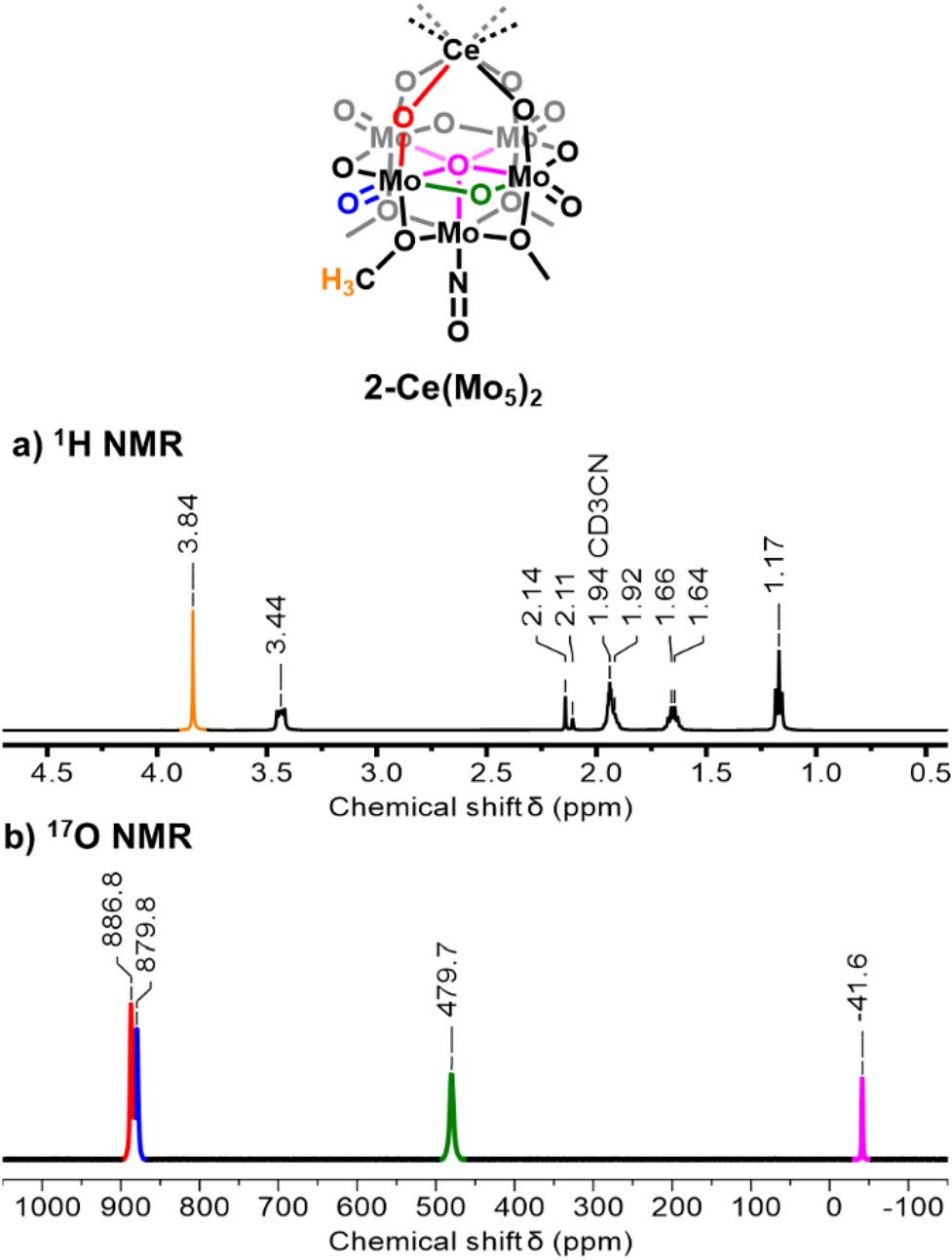
^1^H NMR spectrum (a) and ^17^O NMR spectrum (b) of 2-Ce(Mo_5_)_2_. Spectra were obtained in CD_3_CN at room temperature.

Villanneau and co-workers have previously reported the synthesis of (TBA)_3_[Ce^iii^{Mo_5_O_13_(OMe)_4_NO}_2_] (2-Ce(Mo_5_)_2_) by refluxing 1-NaMo_5_ with half an equivalent of Ce^iv^(SO_4_)_2_.^71^ They verified the reaction also works when using Ce^iii^(NO_3_)_2_·6H_2_O in place of Ce^iv^(SO_4_)_2_, though no experimental description is provided.^71^ To gain familiarity with these Ce containing systems, we first attempted to repeat the synthesis of 2-Ce(Mo_5_)_2_ using a slightly modified procedure. A purple solution of 1-NaMo_5_ in MeOH was added directly to a vial containing half an equivalent of Ce^iii^(OTf)_3_ and the mixture was stirred at 50 °C for two hours. Cooling this solution to −30 °C induced the formation of red/violet crystals (see experimental for more details). Characterization of these crystals using ^1^H NMR spectroscopy (Fig. 1a, S1 and S2) reveals five resonances, four of which are assigned to the TBA cations (at 1–3.5 ppm), and an additional signal observed at 3.84 ppm assigned to the –OMe groups of 2-Ce(Mo_5_)_2_. This signal is shifted in comparison to the corresponding resonance of the analogous Bi^iii^ centered complex (*i.e.*, (TBA)_3_[Bi^iii^{Mo_5_O_13_(OMe)_4_NO}_2_]; *δ* = 4.60 ppm in CD_3_CN),^70^ likely due to the influence of the paramagnetic Ce^iii^ (*f*^1^) center.

We have recently reported that the use of ^17^O enriched 1-NaMo_5_ in the synthesis of M(Mo_5_)_2_ sandwich-type complexes provides access to complexes which are readily characterized by ^17^O NMR spectroscopy. This was pursued for 2-Ce(Mo_5_)_2_ (see experimental section), with the ^17^O NMR spectrum of the obtained product shown in Fig. 1b and S7. The spectrum contains four resonances, consistent with the four oxygen environments. The signals associated with the bridging Mo–O–Mo groups (green) and the μ_5_–O centers (magenta) are assigned based on literature precedent.^68,69,88,89^ Assignment of the Ce–O–Mo and terminal Mo=O groups is more difficult due to the small chemical shift between these two signals (*ca.* 7 ppm). We note that bridging M–O–Mo groups are typically observed upfield (*ca.* 400–700 ppm);^88–91^ the unusually high chemical shift of the oxygen nuclei present in the Ce–O–Mo bridges is likely a result of the influence of the paramagnetic Ce^iii^ center.

In the interpretation of signals of NMR spectra that are paramagnetically broadened or shifted, the observed chemical shift (*δ*) can be decomposed into three components. As described in eqn (1), these are orbital shift (*δ*^orb^), the Fermi contact shift (*δ*^FC^), and the pseudocontact shift (*δ*^PC^).^92,93^

1 *∂* = *∂*^orb^ + *∂*^FC^ + *∂*^PC^

The orbital shift can be thought of as the typical chemical shift in the corresponding diamagnetic compound and is approximately temperature independent. Together, the Fermi contact shift and pseudocontact shift, which represent through bond and through space interactions between the nuclear spins and the unpaired electrons of the paramagnetic center respectively, cause deviations in the observed chemical shifts of paramagnetic complexes from that of their diamagnetic counterparts. Importantly, the Fermi contact shift and pseudocontact shift are inversely proportional to distance from the paramagnetic center and to temperature.^92–94^ Considering these factors in the context of the ^17^O NMR spectrum 2-Ce(Mo_5_)_2_, it is fair to assume that the oxygen nuclei of the Ce–O–Mo bridges will be more strongly affected by the paramagnetic Ce(iii) center than the oxygen nuclei of Mo=O groups. Additionally, it is also expected that as temperature increases the contributions of *δ*^FC^ and *δ*^PC^ to the observed chemical shift should decrease, resulting in a spectrum that appears more similar to that of a diamagnetic complex. In the case of 2-Ce(Mo_5_)_2_, this means that the peak corresponding to the Ce–O–Mo bridges should be much more sensitive to temperature than that of the Mo=O groups, moving upfield (*i.e.*, to a position more typical of M–O–Mo bridges) as temperature increases.

To verify this, variable temperature (VT) ^17^O NMR spectroscopy was performed on a sample of 2-Ce(Mo_5_)_2_ in CD_3_CN (Fig. S11). As expected, the signals observed at 886.8 ppm and 879.6 ppm in Fig. 1b (collected 19.4 °C) behave differently during VT experiments. The resonance that was originally observed at 886.8 ppm in Fig. 1b shows a strong temperature dependence, moving from 905.6 ppm at −17.5 °C to 863.1 ppm at 80.1 °C. This *ca.* 40 ppm shift upfield over the temperature range studied is consistent with the expected behavior of the oxygen nuclei of the Ce–O–Mo bridges. Conversely, the peak observed at 879.6 ppm in Fig. 1b is less sensitive to temperature, moving only *ca.* 3.5 ppm downfield as temperature is increased from −20 °C → 80 °C. As such, we assign this signal to the Mo=O groups. The other oxygen nuclei present in 2-Ce(Mo_5_)_2_ behave similarly to the Mo=O groups, showing downfield shifts of *ca.* 6 ppm and 36 ppm for the Mo–O–Mo and μ_5_–O groups respectively. We note that the magnitude of these shifts increases as the distance of the respective oxygen nuclei to the Ce^iii^ center decreases.

**Fig. 2:**
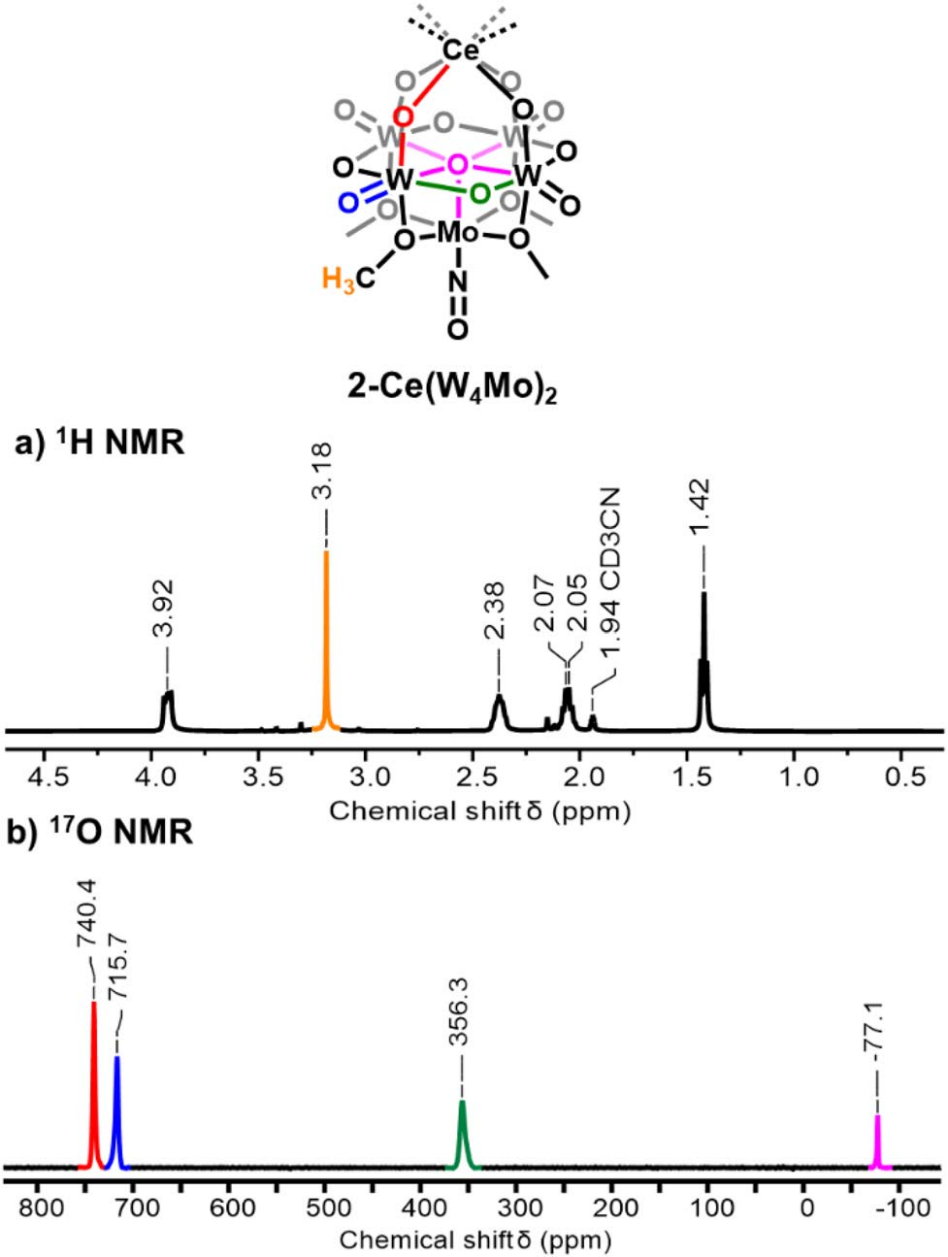
^1^H NMR spectrum (a) and ^17^O NMR spectrum (b) of 2-Ce(W_4_Mo)_2_. Spectra were obtained in CD_3_CN at room temperature.

We next set out to extend the family of Ce sandwich-type complexes through the synthesis of tungsten-containing analogue (TBA)_3_[Ce^iii^{W_4_O_13_(OMe)_4_MoNO}_2_] (2-Ce(W_4_Mo)_2_). Following the methods described above, 1-NaW_4_Mo was treated with 0.55 eq. of Ce^iii^(OTf)_3_ in MeOH at 50 °C, resulting in the formation of a blue/purple solution. Cooling the solution to −30 °C led to the formation of blue/purple crystals. The crystals were analyzed by ^1^H NMR spectroscopy (Fig. 2a, S4 and S5). The spectrum is very similar to that of 2-Ce(Mo_5_)_2_, with five signals in the correct ratio to be assigned to the expected resonances of the TBA cations and –OMe groups of the product. Interestingly, the signals assigned to the TBA cations of 2-Ce(W_4_Mo)_2_ are systematically shifted downfield compared to the corresponding resonances in the ^1^H NMR spectrum of 2-Ce(Mo_5_)_2_, while an upfield shift is observed for the –OMe signal (3.18 ppm for 2-Ce(W_4_Mo)_2_*vs.* 3.84 ppm for 2-Ce(Mo_5_)_2_). This observation suggests that the influence of the paramagnetic Ce^iii^ center changes as a function of the framework metal present in the system.

^17^O NMR spectroscopy was performed on ^17^O enriched 2-Ce(W_4_Mo)_2_ (Fig. 2b and S9). The spectrum is very similar to that of 2-Ce(Mo_5_)_2_, with four peaks observed that can be readily assigned to the oxygen nuclei of the {W_4_Mo} units which can undergo isotopic enrichment during the synthesis, these being the Ce–O–W bridges (red), the W–O–W bridges (green), the terminal W=O groups (blue), and the central μ_5_–O nuclei (magenta) (Fig. 2b). There is less ambiguity in the assignment of the Ce–O–W groups *vs.* the W=O groups in 2-Ce(W_4_Mo)_2_ considering the larger chemical shift difference between the two peaks (*ca.* 26 ppm) in the ^17^O NMR spectrum and the VT ^17^O NMR study performed on 2-Ce(Mo_5_)_2_. The major difference between the ^17^O NMR spectra of 2-Ce(W_4_Mo)_2_ and 2-Ce(Mo_5_)_2_ is the systematic shifting of all peaks upfield, with the effect more pronounced for the resonances observed at higher chemical shifts. This observation is common when comparing the ^17^O NMR spectra of isostructural polyoxotungstates (POTs) and polyoxomolybdates (POMos), and is attributed to the longer, more ionic, W–O bonds of these compounds.^88,89^

**Fig. 3:**
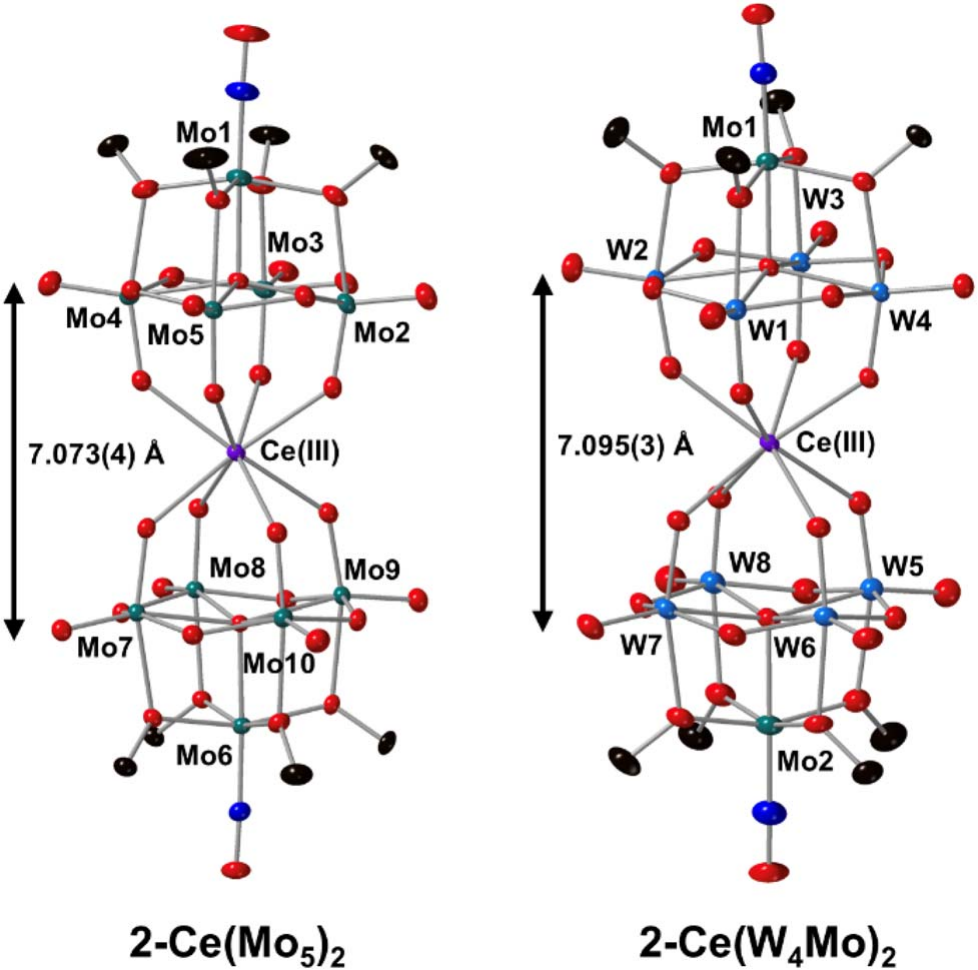
SCXRD structures of 2-Ce(Mo_5_)_2_ and 2-Ce(W_4_Mo)_2_ with probability ellipsoids set at 50%. The tetrabutylammonium cations, solvent molecules and some disorder has been masked for clarity.

Single crystals of 2-Ce(W_4_Mo)_2_ were grown by vapor diffusion of Et_2_O into a saturated solution of the complex dissolved in MeOH. Analysis by single crystal X-ray diffraction (SCXRD) gave, after data refinement, the structure shown in Fig. 3. The structure of 2-Ce(Mo_5_)_2_ was previously reported by Villanneau and co-workers, though this structure features an unusually long terminal nitrosyl bond of 1.35(4) Å. This is *ca.* 0.14–0.15 Å longer than the corresponding bonds in 2-Ce(W_4_Mo)_2_ and analogous M^iv^ centered complexes (*i.e.* (TBA)_2_[M{M′_4_O_13_(OMe_4_)MoNO}_2_] M = Zr^iv^, Hf^iv^, Th^iv^, U^iv^, M′ = Mo^vi^ or W^vi^). We therefore re-acquired structural data for 2-Ce(Mo_5_)_2_. The obtained structure is also shown in Fig. 3 and features terminal nitrosyl bond lengths that are the same, within error, as those in 2-Ce(W_4_Mo)_2_. In both structures, the cerium center occupies an approximately square antiprismatic coordination environment, with average Ce–O bonds lengths of *ca.* 2.48 Å. This illustrates that framework metal substitution has practically no influence on the local coordination environment at Ce. Other Ce^iii^ centered sandwich-type polyoxometalate complexes present in the literature (*e.g.* [Ce(W_5_O_18_)_2_]^6−^, [Ce(α-PW_11_O_39_)_2_]^11−^, *cis*- or *trans*- [Ce(α_2_-P_2_W_17_O_61_)]^17−^) also feature a square antiprismatic coordination environment at Ce, with average Ce–O bond lengths of *ca.* 2.47–2.49 Å.^53,54,95–98^ These distances resemble those of 2-Ce(Mo_5_)_2_ and 2-Ce(W_4_Mo)_2_ which, along with the presence of three TBA cations per sandwich complex, support the presence of trivalent cerium center.

With full structural characterization in hand, we turned our attention to examination of the electronic structure of the Ce(iii)-centered sandwich-type complexes. We first recorded the electronic absorption spectra of 2-Ce(Mo_5_)_2_ and 2-Ce(W_4_Mo)_2_, with the obtained spectra shown in Fig. S13 and S16 (and in Fig. 5, black lines). The main feature in both spectra is a broad low intensity transition centered at 552 nm (*ε* = 144 mol^−1^ dm^3^ cm^−1^) for 2-Ce(Mo_5_)_2_ and 564 nm (*ε* = 129 mol^−1^ dm^3^ cm^−1^) for 2-Ce(W_4_Mo)_2_. This peak has been observed in 1-NaMo_5_, 1-NaW_4_Mo, and a number of the corresponding M^ii^, M^iii^, and M^iv^ sandwich-type complexes.^68–71,76^ We have previously shown this peak can be attributed to transitions originating from occupied orbitals localized on the {Mo–NO}^4^ units.^68^ However, in our previous work, we have shown that the energy of this transition is not sensitive to the framework metal, observing close to identical *λ*_max_ values for this transition in pairs of isostructural sandwich-type complexes.^68^ Therefore, the 12 nm difference between the *λ*_max_ values of 2-Ce(Mo_5_)_2_ and 2-Ce(W_4_Mo)_2_ is surprising. Both spectra also show an intense absorption below 400 nm. This intense absorption is often observed in the electronic absorption spectra of polyoxometalate compounds and is attributed to O(2p) → M(4d/5d) (M = Mo or W) ligand-to-metal charge transfer (LMCT).^68,99,100^ This feature likely obscures resolution of characteristic Ce(iii) 4f → 5d transitions, which typically occur at *ca.* 300–400 nm.^101–107^

**Fig. 4:**
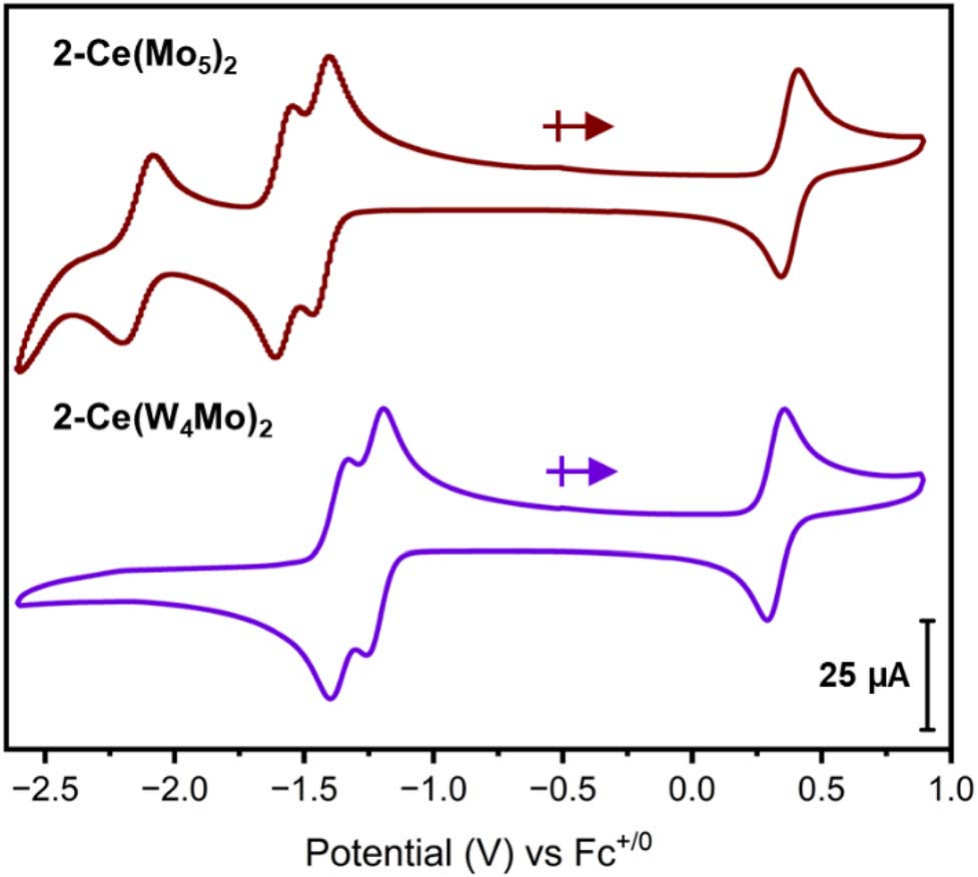
Cyclic voltammograms of 2-Ce(Mo_5_)_2_ (maroon) and 2-Ce(W_4_Mo)_2_ (purple). The data was acquired in MeCN with 0.1 M TBA(PF_6_) supporting electrolyte, 1 mM of cluster, and a scan rate of 200 mV s^−1^.

The redox properties of the Ce^iii^ centered complexes were assessed using cyclic voltammetry on 2-Ce(Mo_5_)_2_ and 2-Ce(W_4_Mo)_2_ (Fig. 4). The CVs of both complexes possess multiple reduction events. The most obvious differences are the potentials of the first two reduction processes of 2-Ce(Mo_5_)_2_ (*E*_1/2_ = −1.44 V, −1.59 V *vs.* Fc^+/0^) compared to 2-Ce(W_4_Mo)_2_ (*E*_1/2_ = −1.21 V, −1.33 V *vs.* Fc^+/0^) and the presence of an additional pseudo-reversible reduction event at −2.18 V *vs.* Fc^+/0^ in the CV of 2-Ce(Mo_5_)_2_. The observation of a third reduction event in the CV of 2-Ce(Mo_5_)_2_ suggests that a higher energy LUMO (*e.g.* LUMO+2) is more accessible for 2-Ce(Mo_5_)_2_ than in 2-Ce(W_4_Mo)_2_.

When comparing these CV's to those of An^iv^ centered sandwich-type complexes with the general formula (TBA)_2_[M{M′_4_O_13_(OMe)_4_MoNO}_2_] (M = Th, U, Np and M′ = Mo or W), both 2-Ce(Mo_5_)_2_ and 2-Ce(W_4_Mo)_2_ have access to one less redox event than their An^iv^ counterparts and the observed redox events are shifted cathodically by *ca.* 0.5–0.75 V.^68–70^ These discrepancies are likely a result of the increased overall negative charge of complex, caused by incorporation of Ce^iii^*vs.* An^iv^. Interestingly, the potentials of the 1st and 2nd reduction events are also closer together (Δ*E* = 0.15 V and 0.12 V for 2-Ce(Mo_5_)_2_ and 2-Ce(W_4_Mo)_2_) than the corresponding reduction events of the An^iv^ centered analogues (Δ*E* = 0.44–0.53 V and 0.19 V for An(Mo_5_)_2_ and An(W_4_Mo)_2_ systems, respectively). This may be a result of the increased distance between the two halves of the sandwich-type complexes in 2-Ce(Mo_5_)_2_ (μ_5_–O → μ_5_–O = 7.073(4) Å) and 2-Ce(W_4_Mo)_2_ (μ_5_–O → μ_5_–O = 7.095(3) Å) compared to the An(Mo_5_)_2_ (average μ_5_–O → μ_5_–O *ca.* 6.87 Å) and An(W_4_Mo)_2_ (average μ_5_–O → μ_5_–O *ca.* 6.88 Å) systems.^68–70^ If the 1st and 2nd reduction events correspond to sequential addition of an electron to each polyoxoalkoxide unit, then increasing the distance between these units should serve to electronically decouple the events, leading to smaller potential differences between the 1st and 2nd reduction processes. The higher sensitivity to this distance observed in the case of the all-molybdenum system can be rationalized by considering the nature of the LUMO/LUMO+1 in these sandwich-type complexes, as previously reported.^68^ In all molybdenum complexes (like 2-Ce(Mo_5_)_2_), these orbitals are localized on the equatorial planes formed by the molybdenum centers either side of the central metal.^68^ Given these orbitals are spatially close, changing the separation between the polyoxoalkoxide units is likely to drive large changes in the energy difference between the 1st and 2nd reduction events. Conversely, in tungsten-containing systems (like 2-Ce(W_4_Mo)_2_), the LUMO and LUMO+1 are localized on the peripheral Mo centers of the {Mo–NO}^4^ units.^68^ The large innate spatial separation between these orbitals caused by the change in electronic structure means these orbitals are already more electronically decoupled and therefore less sensitive to changes in the μ_5_–O → μ_5_–O distance.

The CVs both possess a chemically reversible Ce^iv^/Ce^iii^ redox couple, occurring at 0.38 V *vs.* Fc^+/0^ (Δ*E*_p_ = 65 mV, *I*_p,a_/*I*_p,c_ = 1.08) for 2-Ce(Mo_5_)_2_ and 0.32 V *vs.* Fc^+/0^ (Δ*E*_p_ = 65 mV, *I*_p,a_/*I*_p,c_ = 1.07) 2-Ce(W_4_Mo)_2_. Other Ce^iv^/Ce^iii^ couples reported for Ce(POM)_2_ sandwich-type complexes range from 0.28 V to 0.86 V *vs.* SCE (saturated calomel electrode), however direct comparison of these values to those of 2-Ce(Mo_5_)_2_ and 2-Ce(W_4_Mo)_2_ is difficult given the different reference electrode and the fact all of these values are measured in water (typically 0.1 M KCl or buffered pH 4.5 solutions).^5,55,108,109^ Comparing the potentials of the Ce^iv^/Ce^iii^ redox couples of 2-Ce(Mo_5_)_2_ and 2-Ce(W_4_Mo)_2_ to those of other Ce complexes supported by organic (anionic) oxygen bearing ligands where the redox couples were reported in MeCN shows the values for 2-Ce(Mo_5_)_2_ and 2-Ce(W_4_Mo)_2_ are more positive. For these organic complexes, Ce^iv^/Ce^iii^ redox couples can occur anywhere from −0.56 V *vs.* Fc^+/0^ to −1.18 V *vs.* Fc^+/0^.^5,110,111^ This suggests that polyanionic organic ligands are better at stabilizing Ce^iv^ than the polyoxoalkoxide clusters present in 2-Ce(Mo_5_)_2_ and 2-Ce(W_4_Mo)_2_. However, the observed reversibility of the Ce^iv^/Ce^iii^ redox couples and the stability of the Ce^iv^ complexes (see below) suggest 2-Ce(Mo_5_)_2_ and 2-Ce(W_4_Mo)_2_ could potentially be applied as redox mediators or electrocatalysts in the future.^18,112–114^

### One-electron oxidation of 2-Ce(Mo_5_)_2_ and 2-Ce(W_4_Mo)_2_

**Fig. 5:**
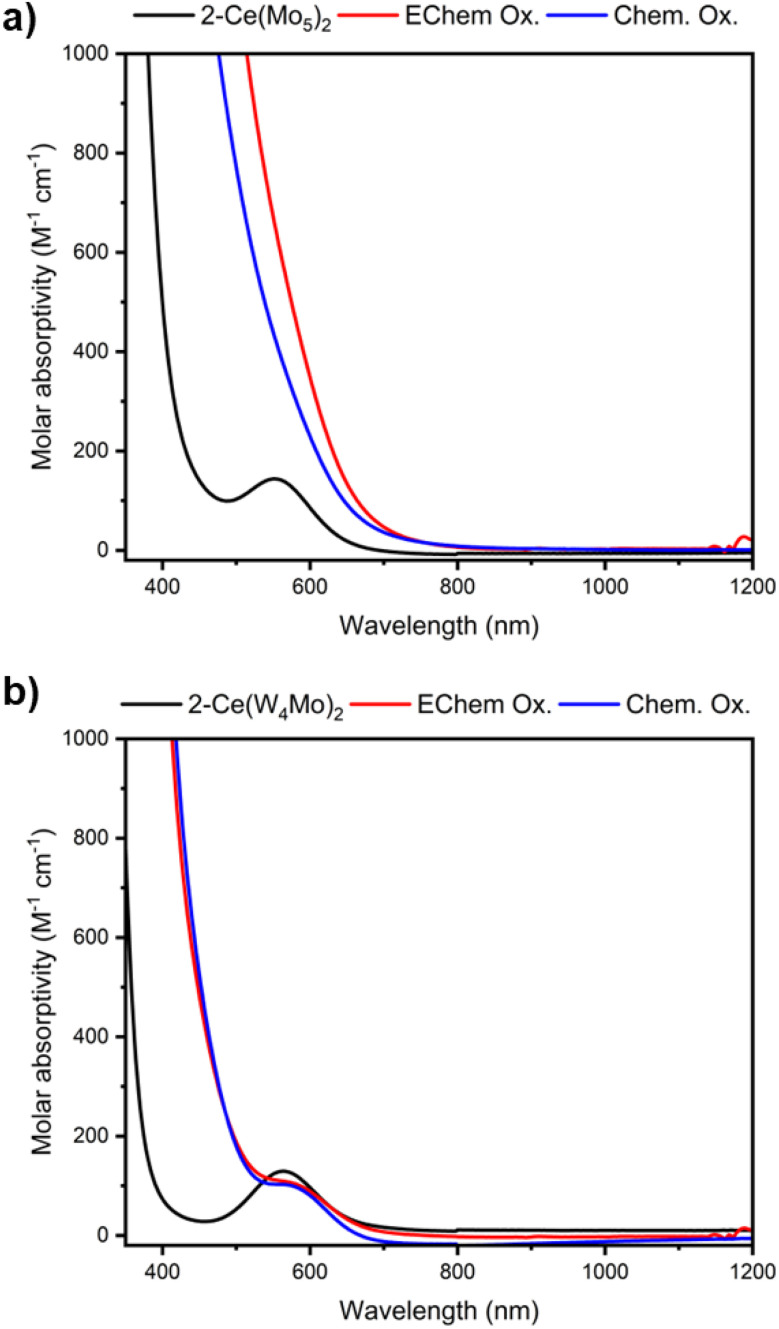
UV-vis spectra of pristine 2-Ce(Mo_5_)_2_ (a, black) and 2-Ce(W4Mo)_2_ (b, black) compared to those obtained after electrochemical oxidation (red) or chemical oxidation by tris(4-bromophenyl)ammoniumyl hexachloroantimonate (blue). All spectra were recorded in MeCN at room temperature.

Given the CVs of 2-Ce(Mo_5_)_2_ and 2-Ce(W_4_Mo)_2_ possess a reversible Ce^iv^/Ce^iii^ redox couple, we sought to investigate one electron oxidation of these complexes to give access to the corresponding Ce^iv^ centered sandwich-type assemblies. Initially, we attempted electrochemical oxidation. Performing bulk oxidation on 1 mM solutions of 2-Ce(Mo_5_)_2_ or 2-Ce(W_4_Mo)_2_ in MeCN/0.1 M TBA(PF_6_) at *ca.* 0.7 V *vs.* Fc^+/0^ leads a to a color change, with the red/violet and blue/purple solutions of 2-Ce(Mo_5_)_2_ and 2-Ce(W_4_Mo)_2_ turning orange and yellow respectively. Inspections the CVs of the solutions after bulk oxidation (Fig. S24 and S25) shows they are almost identical to those of pristine 2-Ce(Mo_5_)_2_ and 2-Ce(W_4_Mo)_2_, suggesting the complex is stable under these conditions. The only major difference is that the position of the open circuit potential has moved to 0.47 V and 0.42 V respectively, suggesting successful formation of the target Ce^iv^ centered complexes, referred to as 3-Ce(Mo_5_)_2_ and 3-Ce(W_4_Mo)_2_. The changes in color observed upon oxidation lead to changes in the electronic absorption spectra of the materials, as shown in Fig. 5a and b (red lines). The broad peak centered at 552 nm in the electronic absorption spectrum of 2-Ce(Mo_5_)_2_ is obscured in by a new charge transfer process that onsets at *ca.* 700 nm. Similarly, the peak that was present in the electronic absorption spectrum of 2-Ce(W_4_Mo)_2_ (564 nm) appears to be slightly red shifted in the spectrum 3-Ce(W_4_Mo)_2_ and is now a shoulder on the side of a new intense absorption feature. These intense absorptions are likely caused by a LMCT process between filled orbitals of the polyoxoalkoxide ligands and the new, low-lying 4f based LUMO centered on Ce.

To confirm this, TD-DFT calculations were performed on 3-Ce(Mo_5_)_2_ and 3-Ce(W_4_Mo)_2_ after structural optimization. For 3-Ce(Mo_5_)_2_, the simulated electronic absorption spectrum shows intense absorption below 500 nm which is caused primarily caused by two distinct types of transitions (Fig. S26). The dominant contribution is confirmed to be a LMCT process involving transitions from occupied molecular orbitals localized on the POM cage (*e.g.* HOMO-7) to unoccupied Ce^iv^ 4f orbitals (Fig. S27). The secondary contribution involves transitions from orbitals localized on the {Mo–NO}^4^ unit (*e.g.* HOMO-1) to vacant orbitals largely localized on the other Mo centers (Fig. S28). For the 3-Ce(W_4_Mo)_2_, the primary contribution is also a LMCT to unoccupied Ce^iv^ 4f orbitals but originates from occupied orbitals more delocalized across the whole polyoxoalkoxide cage (Fig. S32 and S33). This transition occurs at higher energy than in the all-molybdenum system explaining the difference in the onset of the intense absorption observed in the UV-vis spectra of 3-Ce(Mo_5_)_2_ and 3-Ce(W_4_Mo)_2_. Both simulated spectra also feature less intense absorptions at *ca.* 600 nm (Fig. S29 and S34). These are readily assigned to LMCT between filled orbitals localized on the {Mo–NO}^4^ units and empty Ce 4f orbitals (Fig. S30 and S35). This is likely the cause for shoulder observed at *ca.* 568 nm on the side of the intense absorption present in the electronic absorption spectrum of 3-Ce(W_4_Mo)_2_ (Fig. 5b). A separate feature is not seen in the electronic absorption spectrum 3-Ce(Mo_5_)_2_ (Fig. 5a), as it is likely obscured by the more intense LMCT process.

**Fig. 6:**
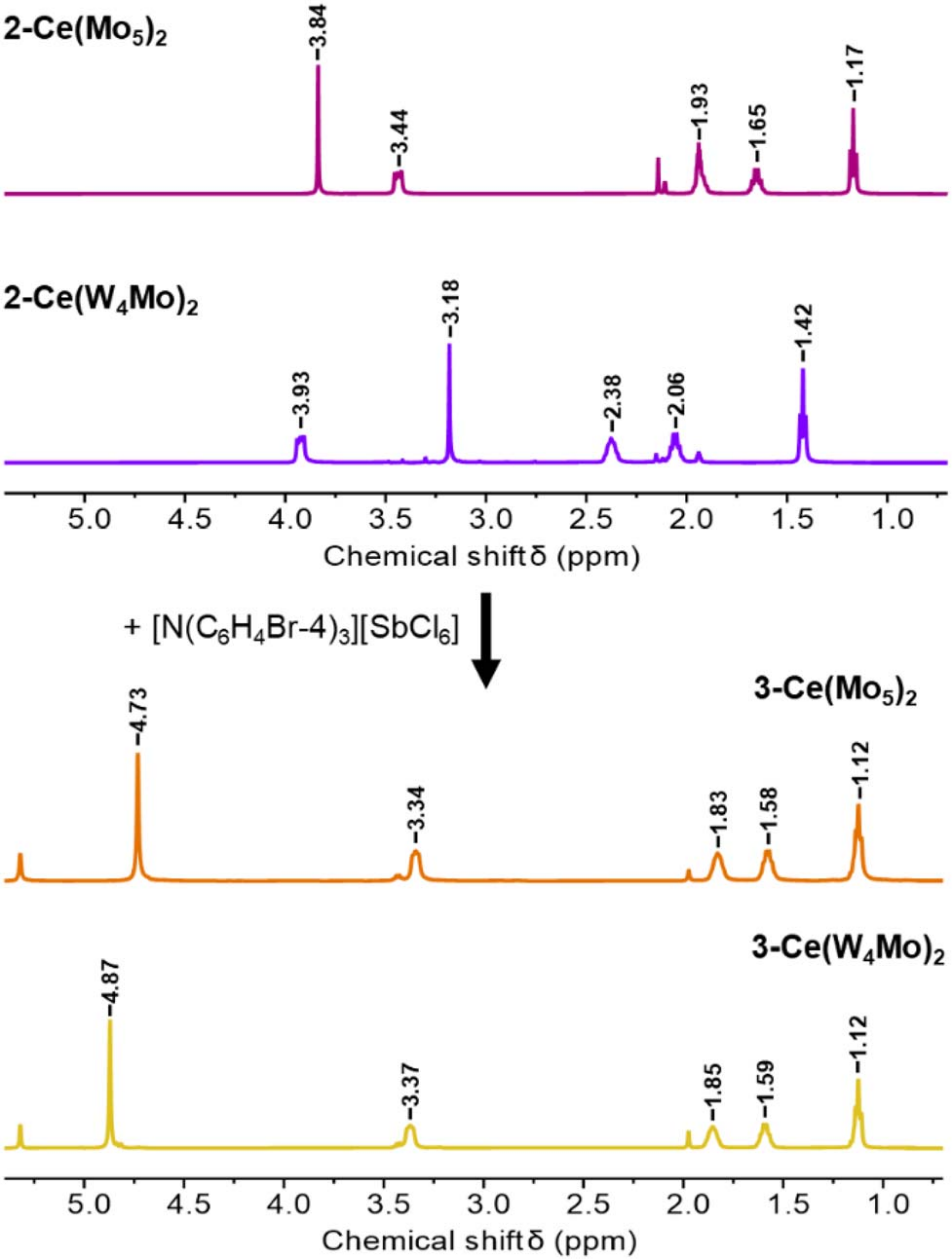
^1^H NMR spectra following the one electron oxidation of 2-Ce(Mo_5_)_2_ and 2-Ce(W_4_Mo)_2_ by tris(4-bromophenyl)ammoniumyl hexachloroantimonate to give the Ce^iv^ centered complexes 3-Ce(Mo_5_)_2_ and 3-Ce(W_4_Mo)_2_. Spectra of the Ce^iii^ complexes were obtained in CD_3_CN, while the Ce^iv^ complexes were obtained in CD_2_Cl_2_. All spectra were recorded at room temperature.

We next pursed isolation of the Ce(iv) centered complexes. Treatment of either 2-Ce(Mo_5_)_2_ and 2-Ce(W_4_Mo)_2_ with 1.1 equivalents of tris(4-bromophenyl)ammoniumyl hexachloroantimonate in MeCN (*E*_Ox_ = 0.67 V *vs.* Fc^+/0^ in MeCN) leads to an immediate color change.^115^ Following work-up, orange and yellow solutions of 3-Ce(Mo_5_)_2_ and 3-Ce(W_4_Mo)_2_, respectively, are obtained. Characterization of the crude products by ^1^H NMR spectroscopy result in the spectra shown in Fig. 6 (Fig. S3 and S6). The spectra of 3-Ce(Mo_5_)_2_ and 3-Ce(W_4_Mo)_2_ are much more similar than the ^1^H NMR spectra of the respective starting materials, with the peaks associated with the TBA cations (*ca.* 1–3.5 ppm) appearing in almost identical positions. The signals associated with the –OMe groups of the sandwich-type complexes are observed at 4.73 ppm and 4.87 ppm respectively for 3-Ce(Mo_5_)_2_ and 3-Ce(W_4_Mo)_2_. These resonances are now at identical positions to the corresponding peaks in the series of closed-shell M^iv^ centered sandwich-type complexes with the general formula (TBA)_2_[M{M′_4_O_13_(OMe)_4_MoNO}_2_] (M = Zr, Hf, Th and M′ = Mo or W), supporting successful formation of the desired Ce^iv^ sandwich-type complexes.^68,70^ This is further supported by electronic absorption spectroscopy, where spectra of 3-Ce(Mo_5_)_2_ and 3-Ce(W_4_Mo)_2_ obtained from chemical oxidation were almost identical to those obtained from electrochemical oxidation experiments (Fig. 5a and b, blue lines). The Ce(iv) complexes are relatively stable, with a half-life of more than one week when left in solution under inert atmosphere (Fig. S12).

**Fig. 7:**
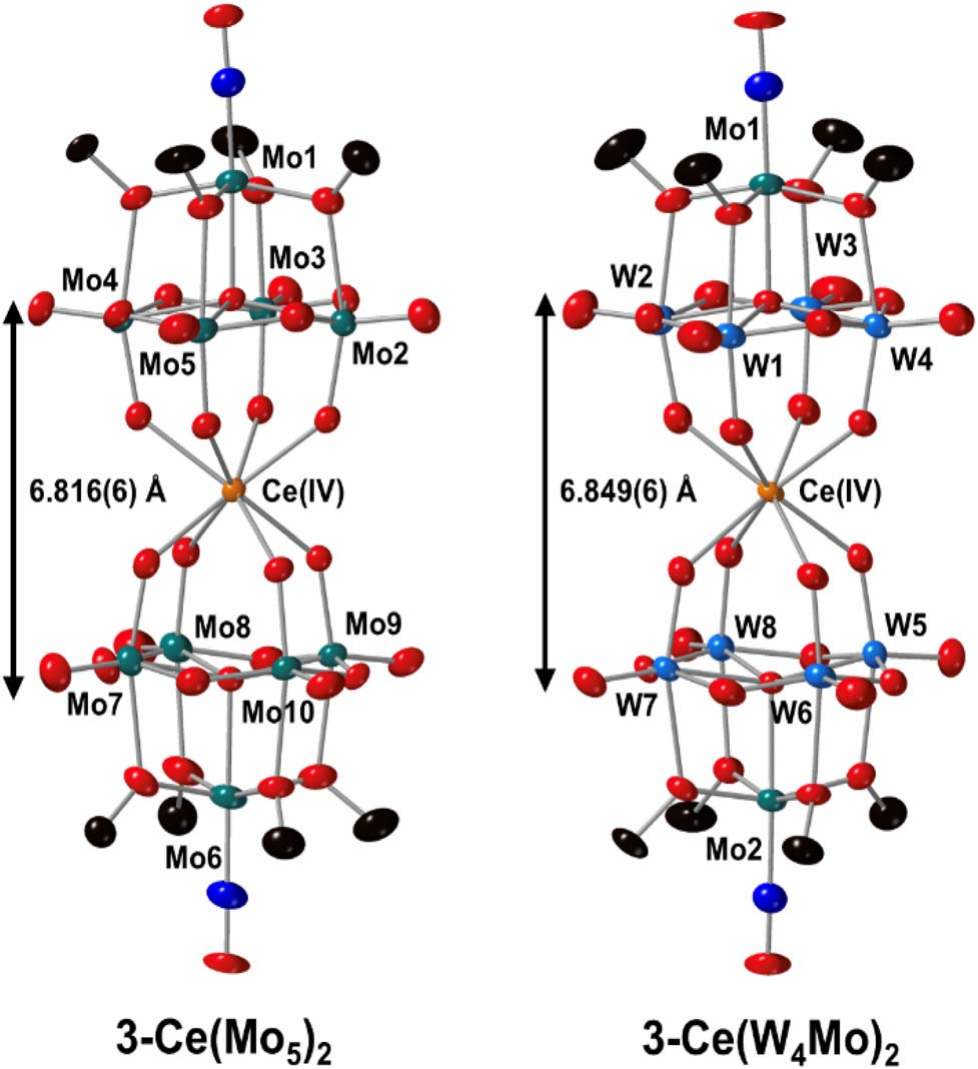
SCXRD structures of the Ce^iv^ centered complexes 3-Ce(Mo_5_)_2_ and 3-Ce(W_4_Mo)_2_ with probability ellipsoids set at 50%. The tetrabutylammonium cations, solvent molecules, and some disorder has been masked for clarity.

To confirm the structures of 3-Ce(Mo_5_)_2_ and 3-Ce(W_4_Mo)_2_, single crystals were grown by vapor diffusion of Et_2_O in to saturated solutions of the complexes dissolved in MeCN. The obtained structures are shown in Fig. 7. The sandwich-type complexes are left mostly unchanged upon oxidation, with the bond distances within the polyoxoalkoxide ligands showing only very minor variations (Table S5). As expected, the main consequence of oxidation of Ce^iii^ to Ce^iv^ is a contraction of the Ce–O bonds, with average Ce–O bond distances of *ca.* 2.34 Å and 2.35 Å respectively for 3-Ce(Mo_5_)_2_ and 3-Ce(W_4_Mo)_2_. The 0.13–0.14 Å decrease in bond lengths compared to 2-Ce(Mo_5_)_2_ and 2-Ce(W_4_Mo)_2_ can be attributed to the decrease in ionic radius accompanied with oxidation of Ce^iii^ (1.14 Å) to Ce^iv^ (0.97 Å).^116^ This Ce–O bond contraction translates to a drop in the distance between the two polyoxoalkoxide units of the sandwich-type complex (approximated by the μ_5_–O → μ_5_–O distance). To quantify the deviation from square antiprismatic geometry, continuous shape measurements (CShM) were calculated (Table S6).^117,118^ Both 3-Ce(Mo_5_)_2_ and 3-Ce(W_4_Mo)_2_ display nearly ideal square antiprismatic about the Ce centers (CShM values = 0.20960 and 0.22048, where a value of 0 reflects the ideal geometry). These values are lower than the corresponding values for 3-Ce(Mo_5_)_2_ and 3-Ce(W_4_Mo)_2_ (CShM values = 0.93012 and 0.42435), illustrating how oxidation of the Ce center influences the overall geometry of the complexes.

### ^17^O NMR spectroscopy and M–O bond covalency in M(iv) centered sandwich-type complexes

**Fig. 8:**
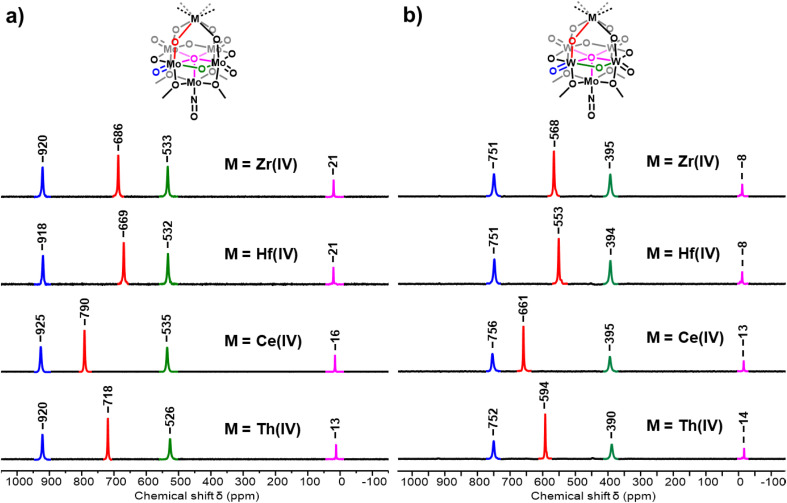
^17^O NMR spectrum of (a) 3-Ce(Mo_5_)_2_ and (b) 3-Ce(W_4_Mo)_2_ compared to other isostructural M^iv^ centered sandwich-type complexes. All spectra were obtained in CD_2_Cl_2_ at room temperature.

After successful isolation the Ce^iv^ centered sandwich-type complexes, 3-Ce(Mo_5_)_2_ and 3-Ce(W_4_Mo)_2_, we sought to isolate ^17^O enriched analogues of the complexes to verify how the ^17^O spectra of these complexes change upon oxidation and how they compare to the spectra of other isostructural M^iv^ centered sandwich-type complexes.^68,69^ Following the procedures discussed above, ^17^O enriched samples of 2-Ce(Mo_5_)_2_ and 2-Ce(W_4_Mo)_2_ were oxidized by one-electron, purified, and their ^17^O NMR spectra were recorded. The obtained spectra are shown in Fig. 8a and b, along with the corresponding spectra of other isostructural, diamagnetic, M^iv^ centered sandwich-type complexes. The peaks assigned to the terminal M = O groups (blue), bridging M–O–M groups (green), and central μ_5_–O groups (magenta) are all shifted downfield in the ^17^O NMR spectra of 3-Ce(Mo_5_)_2_ and 3-Ce(W_4_Mo)_2_ compared to the corresponding signals in the spectra of 2-Ce(Mo_5_)_2_ and 2-Ce(W_4_Mo)_2_ (M = Mo or W, Fig. 1b and 2b). The deshielding of these nuclei can be attributed to the reduction in the anionic charge of the system which accompanies the oxidation of Ce^iii^ to Ce^iv^. The only nuclei that do not follow this logical behavior are the Ce–O–M groups (red). The resonances assigned to these nuclei instead shift upfield. This is likely because oxidation of Ce^iii^ (f^1^) to Ce^iv^ (f^0^) allows these peaks, which were observed at unusually high chemical shifts in the ^17^O NMR spectra of 2-Ce(Mo_5_)_2_ and 2-Ce(W_4_Mo)_2_ due to interactions with the paramagnetic Ce^iii^ center, to move back to positions more typical of bridging oxygen nuclei.^88–91^

When comparing the ^17^O NMR spectra of 3-Ce(Mo_5_)_2_ and 3-Ce(W_4_Mo)_2_ with those of other M^iv^ sandwich-type complexes (Fig. 8a and b, M^iv^ = Zr, Hf, Th)^68,69^ we can see that the positions of the peaks associated with the terminal M = O groups (blue), the bridging M–O–M groups (green), and the central μ_5_–O groups (magenta) are insensitive to the identity of the M^iv^ ion present at the center of the sandwich-type complexes. Indeed, these peaks all occur within *ca.* 10 ppm of each other within each series of sandwich-type complexes (*i.e.* M^iv^(Mo_5_)_2_ or M^iv^(W_4_Mo)_2_). As may be expected, this contrasts the behavior of the M^iv^–O–M groups (red), with the peak assigned to these nuclei varying by >100 ppm across the series. In both series of complexes (*i.e.* M^iv^(Mo_5_)_2_ or M^iv^(W_4_Mo)_2_), the magnitude of the observed chemical shift increases in the order Hf–O–M < Zr–O–M < Th–O–M < Ce–O–M (M = Mo or W). Given this trend persists between the series, it is likely that this relative ordering of the complexes is caused by differences in the M^iv^–O bonding, while the 110–130 ppm difference in chemical shift between the signals of the pairs of M^iv^(Mo_5_)_2_ and M^iv^(W_4_Mo)_2_ complexes can be attributed to differences in the Mo^vi^–O and W^vi^–O bonds.

**Fig. 9:**
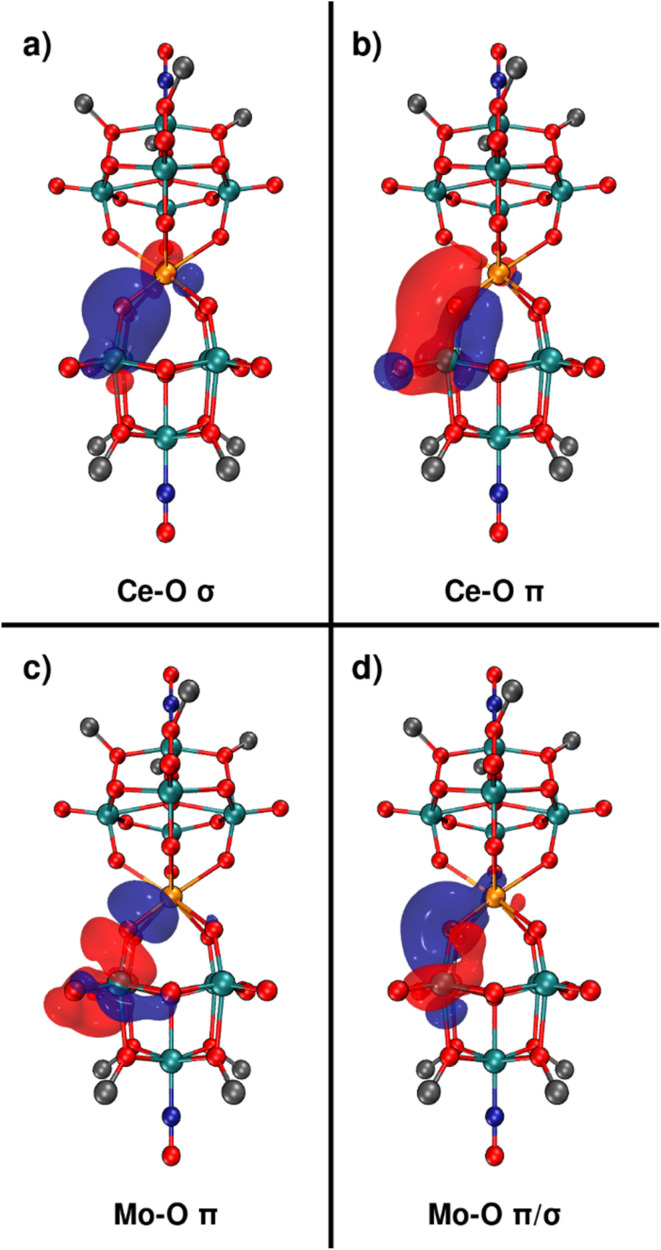
NLMOs which define the Ce^iv^–O bonds (a and b) and Mo^vi^–O bonds (c and d) of the Ce^iv^–O–Mo^vi^ bridges present in 3-Ce(Mo_5_)_2_. Similar graphics for all other complexes discussed in Fig. 8 can be found in Fig. S43–49.

Given the nature of both the M^iv^–O bonds and M^vi^–O bonds present in the sandwich-type complexes appear to strongly influence the experimentally observed ^17^O NMR chemical shifts, we sought to gain further insights into these bonding interactions using DFT calculations. Optimized structures of the M^iv^ centered sandwich complexes discussed in Fig. 8 were first obtained and frequency calculations were performed to ensure the structures represent a minimum on their respective potential energy surfaces. Natural Bond Order (NBO) analysis was then used to obtain metal atom contributions to the natural localized molecular orbitals (NLMOs) (Tables S12–15), specifically focusing on the nature of the M^iv^–O–M^vi^ bonds (M^iv^ = Zr, Hf, Ce, or Th and M^vi^ = Mo or W). The M^iv^–O bonds in our systems are defined by two NLMOs, one σ-type and one π-type (Fig. 9a and b). Both NLMOs are dominated by the s and p orbitals of oxygen, with all metal orbitals contributing <15% in all cases. Of the M^iv^ centers present, Ce^iv^ orbitals (primarily f and d orbitals) contribute more to the NLMOs than any other M^iv^, followed by Th, then Zr, and finally Hf (full details in Fig. S36–S39). This is largely in-line with the results obtained for [M^iv^Cl_6_]^2−^ (M^iv^ = Zr, Hf, and Ce) complexes, where Ce^iv^ contributes much more to M–Cl bonds than Zr^iv^ or Hf^iv^ due to increased f-orbital contribution.^21,24^ Moreover, this follows exactly the order of ^17^O NMR chemical shifts of the M^iv^–O–M^vi^ nuclei presented in Fig. 8, suggesting a correlation between the M^iv^ orbital contribution to M^iv^–O bonds (*i.e.* M–O bond covalency) and the observed chemical shifts.

Investigation of the specific contributions to the NLMOs of the Ce^iv^–O bonds highlights that the d-orbital contributions are similar to those of the Th^iv^–O bonds (see Fig. S40 and Table S17), and thus the overall increase in metal orbital contributions to the Ce^iv^–O bonds is result of increased f-orbital contributions. This finding is consistent with previous studies on f-element-oxide systems, which establish that favorable energy matching between metal-f and oxygen-p orbitals can be a more dominant factor in determining f-orbital contribution to bonding than what the greater spatial extent of orbitals in heavier actinides might imply.^119,120^ The electronic origin of this trend was elucidated by analyzing the Projected Density of States (PDOS). While the highly delocalized electronic structure of the POM framework complicates the analysis of individual canonical molecular orbitals, the PDOS provides a clear picture. Fig. S41 shows that the unoccupied Ce 4f states are energetically closer to the O 2p-dominated valence band than the Th 5f states (Fig. S42). This favorable energy matching for Ce^iv^ promotes more effective orbital mixing, thus explaining the greater f-orbital character observed in the NBO analysis. In contrast, the d-orbital contributions are more comparable for both metals, highlighting that the f-orbitals are the primary differentiators of M–O covalency in these two systems.

This interpretation aligns with the work of Minasian *et al.* on simple lanthanide dioxides, which demonstrated that an 8-coordinate environment is crucial for enabling f-orbital participation in bonding.^31^ While the perfect cubic symmetry in LnO_2_ allows for a clear analysis of specific symmetry-allowed orbital interactions, the highly delocalized valence molecular orbitals and slightly distorted structures (*i.e.* not perfectly *D*_4d_) present in our study make it difficult to identify the specific MOs with O(2p) character that are responsible for symmetry allowed overlap with the metal d/f orbitals. Even if the complex nature of the POM cage orbitals makes it difficult to isolate specific symmetry allowed overlap effects, a qualitative agreement with Minasian *et al.*, supported by NBO analysis and QTAIM DI delocalization indices (see below) provides insight into the importance of 8-coordinate geometry for M-O orbital mixing, and the specific nature of the metal.

The M^vi^–O bonds (M^vi^ = Mo or W) of the M^iv^–O–M^vi^ bridges are similarly defined by two NLMOs, one π-type and one σ/π hybrid (Fig. 9c and d). Again, these orbitals have primarily O character with minor contributions from the M^vi^ center. These orbitals remain consistent across the series of complexes studied, with Mo^vi^ orbitals always contributing more to the NLMOs than W^vi^ orbitals, regardless of the M^iv^ center present. This could explain the fact that ^17^O NMR chemical shifts of the oxygen nuclei of the M^iv^–O–M^vi^ bridges in the M^iv^(Mo_5_)_2_ complexes are 110–130 ppm higher than in the corresponding signal in the ^17^O NMR spectra of the M^iv^(W_4_Mo)_2_ complexes, suggesting that an overall increase in metal orbital contribution to the bonds of the M^iv^–O–M^vi^ bridges drives an increase in the observed ^17^O NMR chemical shift.

**Fig. 10:**
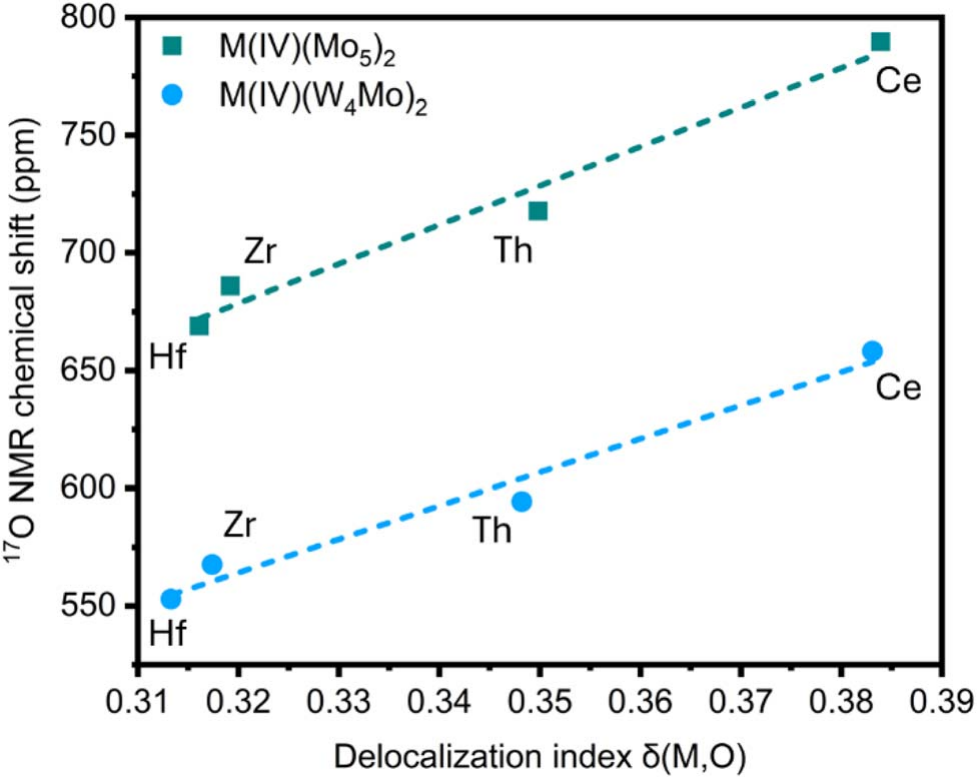
Plot of M^iv^–O delocalization index obtained from QTAIM analysis *vs.*^17^O NMR chemical shift (ppm) for the complexes discussed in this work.

Though the intuitive understanding of orbital mixing is provided by NBO analysis, other metrics have proved effective in the literature for assessing bonding.^121–123^ Specifically, delocalization indices (DI) obtained from Quantum Theory of Atoms in Molecules (QTAIM) analyses, which provide a measure of the number of electron pairs exchanged in an interaction, have been reported as an effective measure of metal bond covalency.^23,25,27,33,122,124,125^ The DI values for the atoms involved in both the M^iv^–O and M^vi^–O bonds of the M^iv^–O–M^vi^ bridges present in the series of complexes studied are given in Table S16. Focusing on the M^iv^–O bonds, plotting DI *vs.*^17^O NMR chemical shift of the oxygen nuclei present in the M^iv^–O–M^vi^ bridges gives the graph shown in Fig. 10. It is immediately apparent that as DI increases, which implies an increase in bond covalency, the observed ^17^O NMR chemical shift also increases, with very good linear fits observed. The observed deshielding of the ^17^O nuclei as M–O bond covalency increases can be attributed to Spin–Orbit Heavy Atom on the Light Atom (SO-HALA) effects.^42,120,126–132^ Specifically, the presence of empty low-lying valence d- or f-orbitals allows the deshielding 
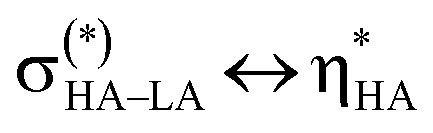
 coupling mechanism to dominate. This leads to relatively small deshieiding effects in 4d^0^/5d^0^ transition metals, but the magnitude increases drastically when moving to 4f^0^ Ce^iv^.^120^ While even larger effects are expected for 5f^0^ systems, moderate deshielding effects (comparable to 5d systems) are often reported for Th^iv^ as bonding is typically dominated by 6d/7s orbitals with only minor contributions of 5f orbitals.^120^ This is in line with the NBO and PDOS analysis presented above and thus the relationship between ^17^O NMR chemical shift and M^iv^–O bond covalency shown in Fig. 10 reproduces reported trends in the magnitude of SO-HALA effects well.

The vertical offset between the two series can be attributed to difference in covalency of the M^vi^–O bonds of the M^iv^–O–M^vi^ bridges. On average, DI values of 1.30 for Mo^vi^–O bonds and 1.24 for W^vi^–O bonds were obtained. This is in line with literature precedent that W^vi^–O bonds are typically more ionic than Mo^vi^–O bonds.^88,89^ Plotting the sum of the DIs of the M^iv^–O and M^vi^–O bonds *vs.*^17^O NMR chemical shift of the oxygen nuclei present in the M^iv^–O–M^vi^ bridges (Fig. S50) allows the two series (*i.e.* M^iv^(Mo_5_)_2_ and M^iv^(W_4_Mo)_2_ complexes) to be treated collectively. A strong correlation between the total DI and ^17^O NMR chemical shift is still observed, however there are some anomalies which suggest that this simplistic approach cannot completely capture the influence that varying the framework metal has on metal–oxygen bonding. The bond covalency trends obtained from our combined experimental and computational analysis are in line with the computational studies from Kaltsoyannis and co-workers, who assessed M–O bond covalency in a series of M(OC_6_H_5_)_4_ (M = Ti^iv^, Zr^iv^, Hf^iv^, Ce^iv^, Th^iv^, Pa^iv^, U^iv^, and Np^iv^), and the experimental study from Schelter, who observed Ce^iv^=N bonds are more covalent than Th^iv^=N bonds.^33,34,123^

## Conclusions

In conclusion, we have built on the original methods of Proust and Villanneau to access the Ce^iii^ centered sandwich-type complexes 2-Ce(Mo_5_)_2_ and 2-Ce(W_4_Mo)_2_.^71^ Cyclic voltammetry revealed the complexes possess a reversible Ce^iv^/Ce^iii^ redox couples at *ca.* 0.35 V *vs.* Fc^+/0^ in MeCN. One-electron chemical oxidation using tris(4-bromophenyl)ammoniumyl hexachloroantimonate provides convenient access to the corresponding Ce^iv^ complexes 3-Ce(Mo_5_)_2_ and 3-Ce(W_4_Mo)_2_. While the optical properties of 2-Ce(Mo_5_)_2_ and 2-Ce(W_4_Mo)_2_ are very similar to the reported sodium complexes 1-NaMo_5_ and 1-NaW_4_Mo,^68,70,71,76^ oxidation to Ce^iv^ leads to the immergence of a new charge transfer process assigned to a LMCT between filled orbitals of the polyoxoalkoxide ligands and the newly empty 4f orbitals of Ce^iv^. ^17^O enriched analogues of the complexes were readily prepared from the corresponding ^17^O enriched starting materials. While analysis of the ^17^O NMR spectra of the Ce^iii^ centered complexes shows the influence of the paramagnetic Ce^iii^ (4f^1^) center, the ^17^O NMR spectra of the diamagnetic Ce^iv^ centered complexes are in line with the spectra of the previously prepared Zr^iv^, Hf^iv^, and Th^iv^ containing complexes.^68,69^ The only major deviation in the spectra of the Ce^iv^ complexes is the high chemical shift of the peaks assigned to the oxygen nuclei of the Ce–O–M^vi^ bridges (M^vi^ = Mo or W). Computational calculations clearly indicate that this increase in chemical shift can be attributed to an increase in the M^iv^–O bond covalency. Further investigations revealed a strong correlation between ^17^O NMR chemical shift and M–O delocalization indices (which act as measure of bond covalency) obtained from QTAIM analysis, in this series of isostructural diamagnetic complexes. These results display the utility of ^17^O NMR spectroscopy as an experimental tool for investigating metal–oxygen bonding and covalency.

## Author contributions

E. M. M. and D. S. conceived of the project. D. S., N. G., and A. C. B. synthesized and characterized all compounds. W. W. B. determined the crystal structures and carried out elemental analysis measurements. Calculations were performed by M. P. and M. T. R., while E. M. M. directed the project. The manuscript was written through contributions of all authors. All authors have given approval to the final version of the manuscript.

## Conflicts of interest

The authors declare no competing financial interest.

## Supplementary Material

SC-OLF-D5SC06415E-s001

SC-OLF-D5SC06415E-s002

SC-OLF-D5SC06415E-s003

## Data Availability

The data supporting this article, including the coordinates of the optimized geometries of the structures discussed in this work obtained in computational studies, have been included as part of the supplementary information (SI). All other data is available upon requests made to the corresponding author. CCDC 2481875–2481878 contain the supplementary crystallographic data for this paper.^133*a*–*d*^ Supplementary information: additional spectroscopic, crystallographic, and computational data (PDF). See DOI: https://doi.org/10.1039/d5sc06415e.
