# Supplementary material for: Isolation of Ce(iv) centered polyoxoalkoxide sandwich-type complexes allows comparison of metal–oxygen bond covalency
Source: Chem Sci. 2025 Oct 13;16(46):21917–31. doi: 10.1039/d5sc06415e (PMC12533601; doi:10.1039/d5sc06415e)
Supplement: SC-016-D5SC06415E-s001 [file SC-016-D5SC06415E-s001.pdf]

Supplementary Information (SI) for Chemical Science.
This journal is © The Royal Society of Chemistry 2025

Electronic Supporting Information

Isolation of Ce(IV) centered polyoxoalkoxide sandwich-type complexes allows comparison of metal-oxygen bond covalency

Dominic Shiels[1]*, Michele Pittalis[1], Nadeeshan Gunarathna[1], Adriana C. Berlfein[1], William W. Brennessel[1], Michael T. Ruggiero[1]*, and Ellen M. Matson[1]*

[1] *Department of Chemistry, University of Rochester, Rochester NY 14627 USA*

Corresponding Author Contact Information:

Dominic Shiels: dshiels@ur.rochester.edu

Michael T. Ruggiero: michael.ruggiero@rochester.edu

Ellen M. Matson: matson@chem.rochester.edu

S1.^{1}H NMR spectra **S3**
S2.^{17}O NMR spectra **S6**
S3. UV-Vis-NIR spectra **S10**
S4. Electrochemistry **S13**
S5. Single Crystal X-ray Diffraction **S17**
S6. Computational calculations **S23**

S1. ^{1}H NMR spectra

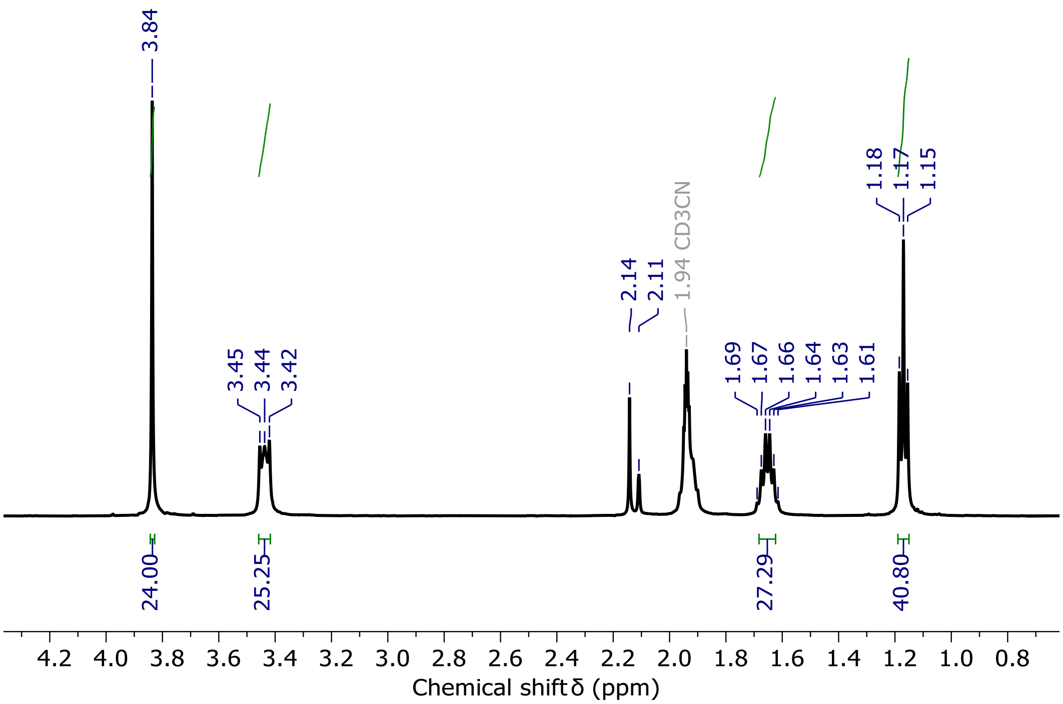

Figure S1. ^{1}H NMR spectrum (500 MHz) of $(TBA)_3[Ce\{Mo_5O_{13}(OMe)_4NO\}_2]$ (**2-Ce(Mo$_5$)$_2$**) in CD_3CN.

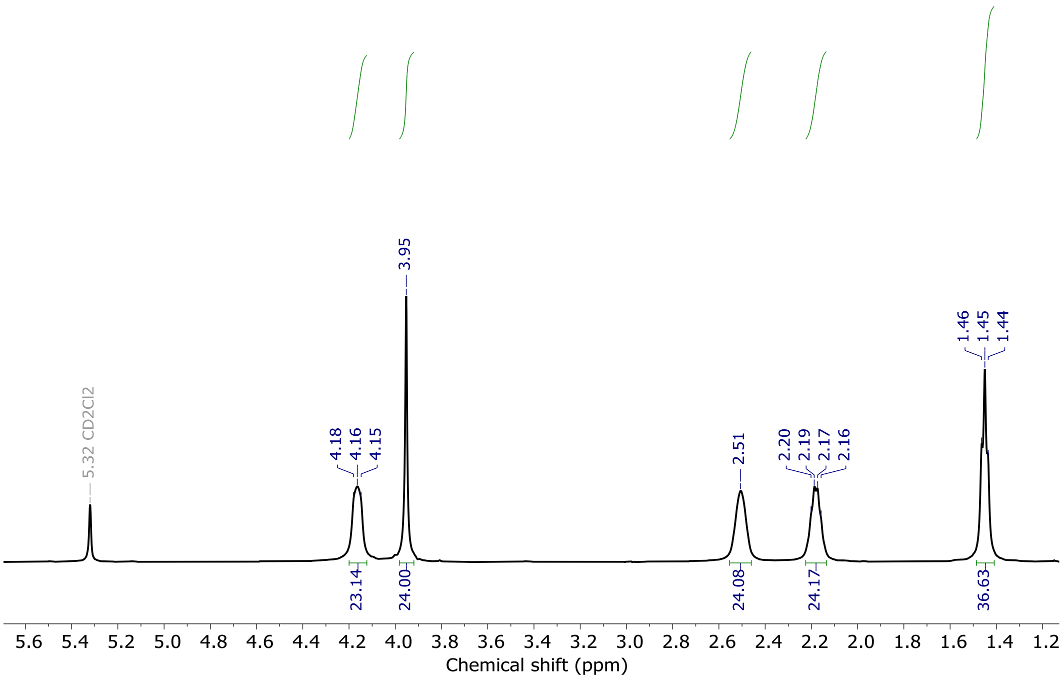

Figure S2. ^{1}H NMR spectrum (500 MHz) of $(TBA)_3[Ce\{Mo_5O_{13}(OMe)_4NO\}_2]$ (**2-Ce(Mo$_5$)$_2$**) in CD_2Cl_2.

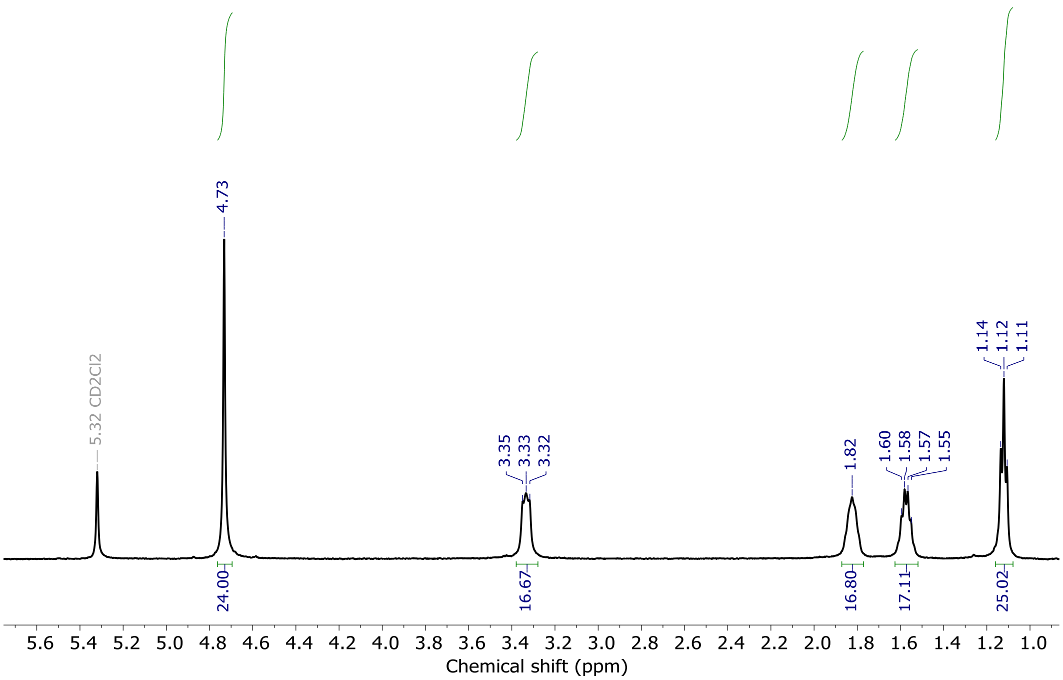

Figure S3: ^{1}H-NMR spectrum (500 MHz) of $(TBA)_2[Ce\{Mo_5O_{13}(OMe)_4NO\}_2]$ (**3-Ce(Mo$_5$)$_2$**) in CD_2Cl_2.

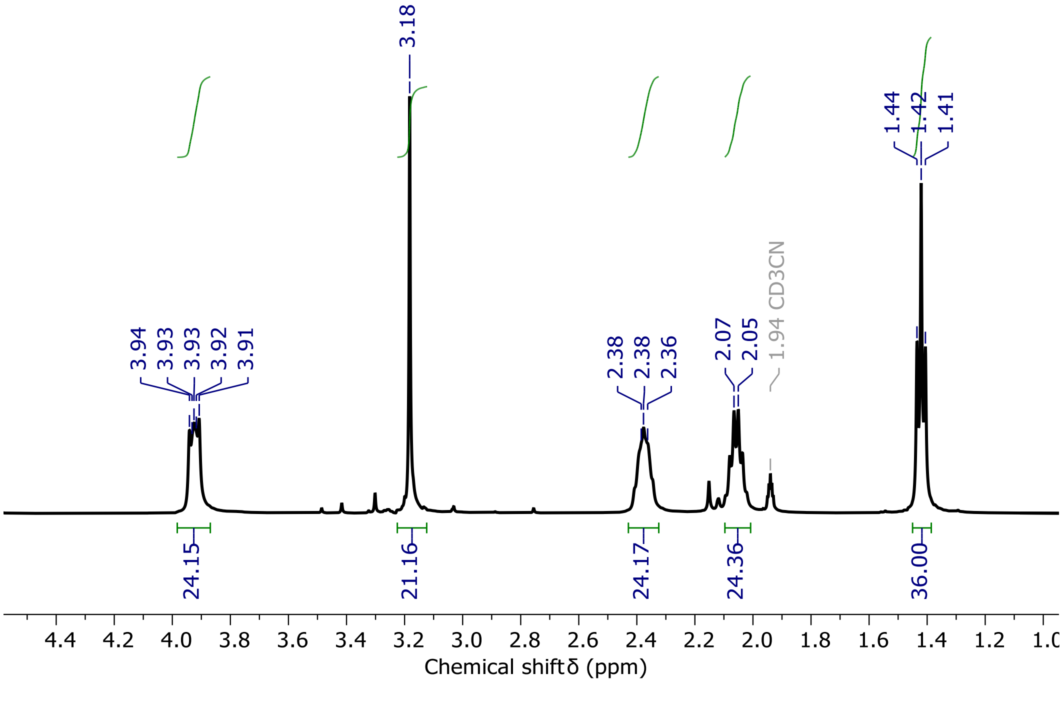

Figure S4.^{1}H NMR spectrum (500 MHz) of $(TBA)_3[Ce\{W_4O_{13}(OMe)_4MoNO\}_2]$ (**2-Ce(W$_4$Mo)$_2$**) in CD_3CN.

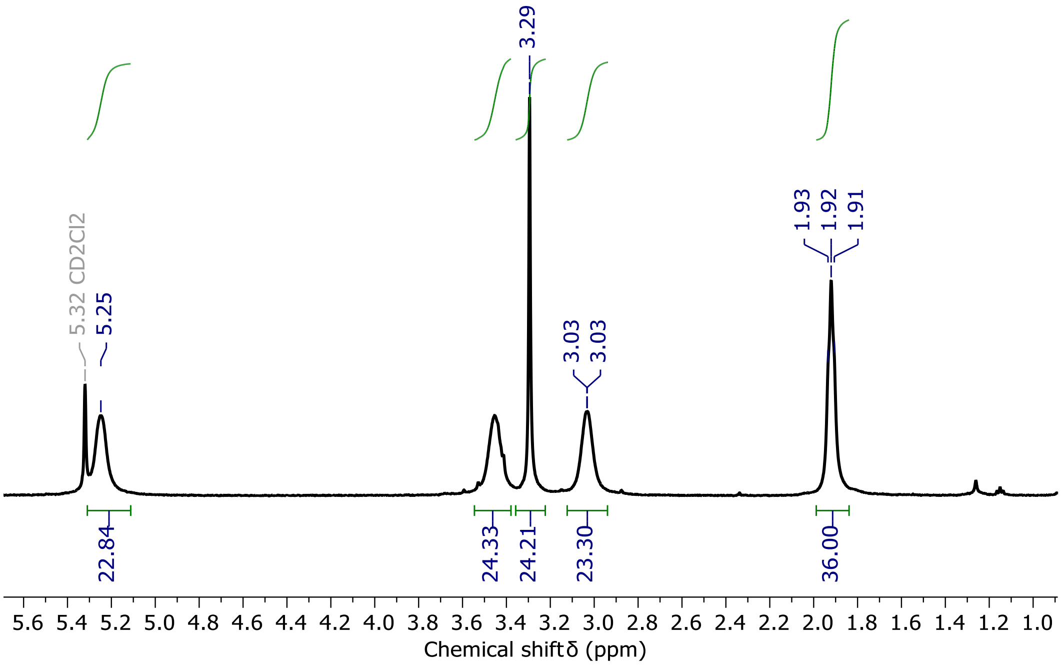

Figure S5. ^{1}H NMR spectrum (500 MHz) of $(TBA)_3[Ce\{W_4O_{13}(OMe)_4MoNO\}_2]$ (**2-Ce(W$_4$Mo)$_2$**) in CD_2Cl_2.

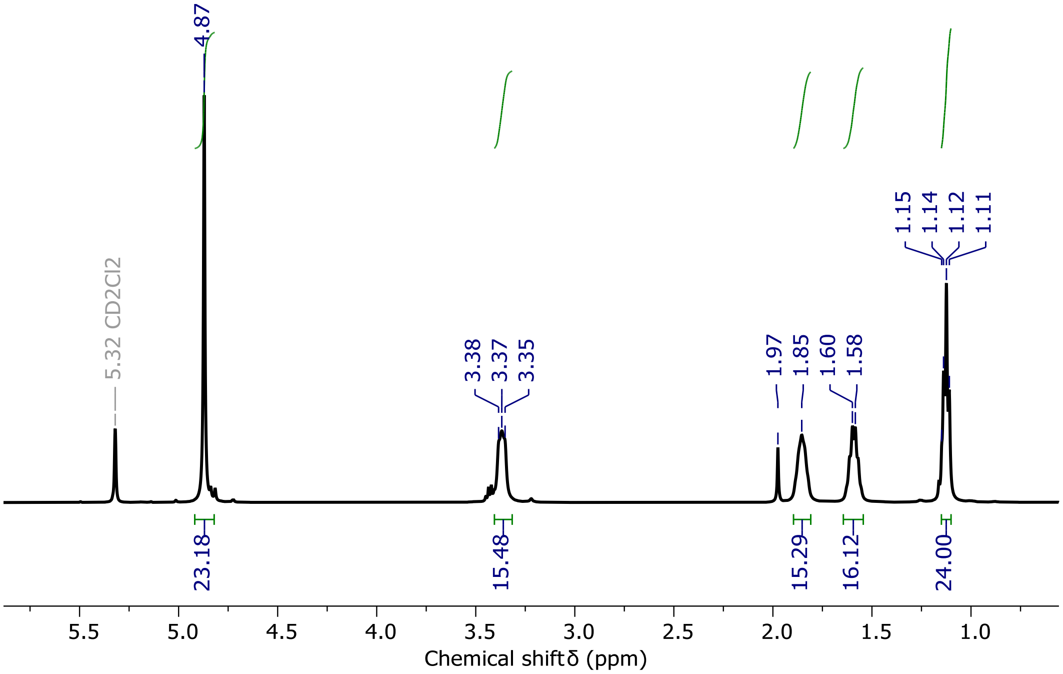

Figure S6. ^{1}H NMR spectrum (500 MHz) of $(TBA)_2[Ce\{W_4O_{13}(OMe)_4MoNO\}_2]$ (**3-Ce(W$_4$Mo)$_2$**) in CD_2Cl_2.

S2. ^{17}O NMR spectra

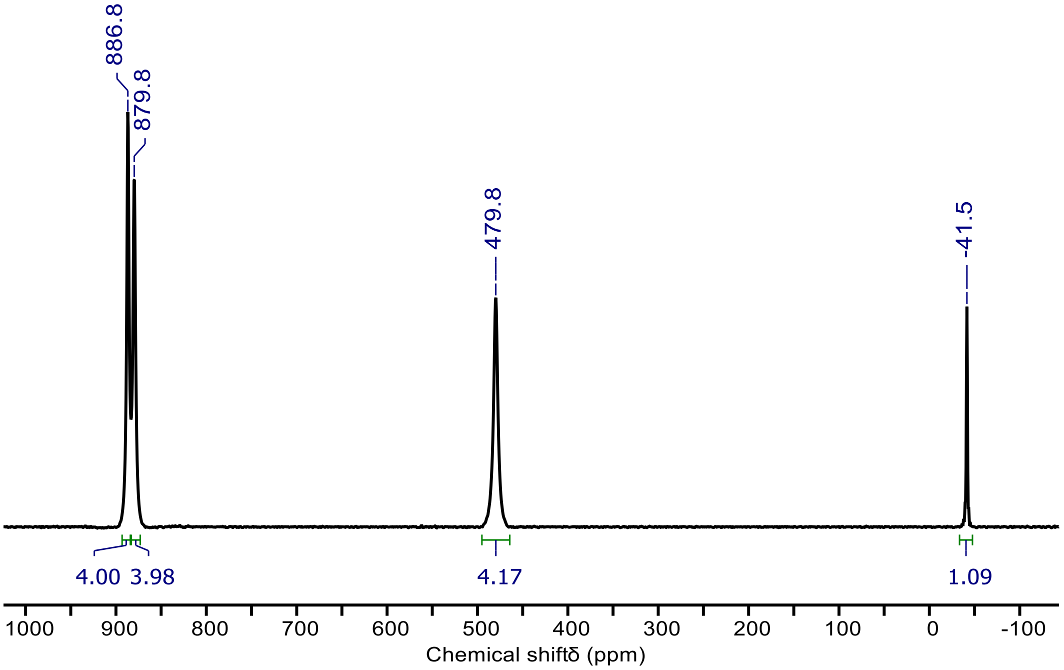

Figure S7. ^{17}O NMR spectrum (500 MHz) of $(TBA)_3[Ce\{Mo_5O_{13}(OMe)_4NO\}_2]$ (**2-Ce(Mo$_5$)$_2$**) in CD_3CN at 19.4 °C.

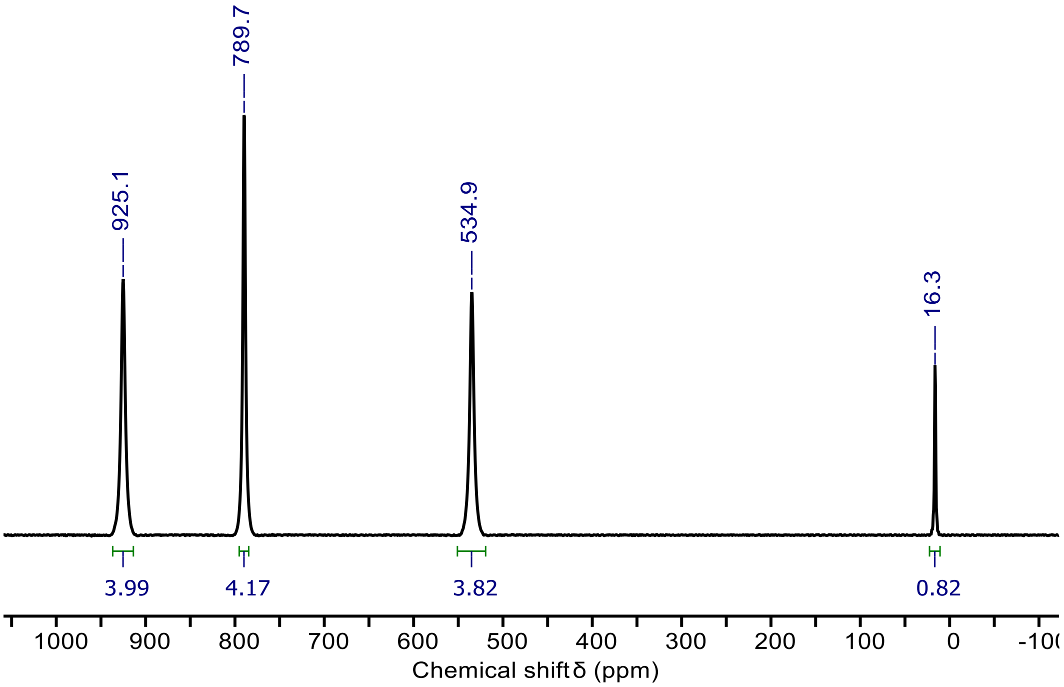

Figure S8. ^{17}O NMR spectrum (500 MHz) of $(TBA)_2[Ce\{Mo_5O_{13}(OMe)_4NO\}_2]$ (**3-Ce(Mo$_5$)$_2$**) in CD_2Cl_2.

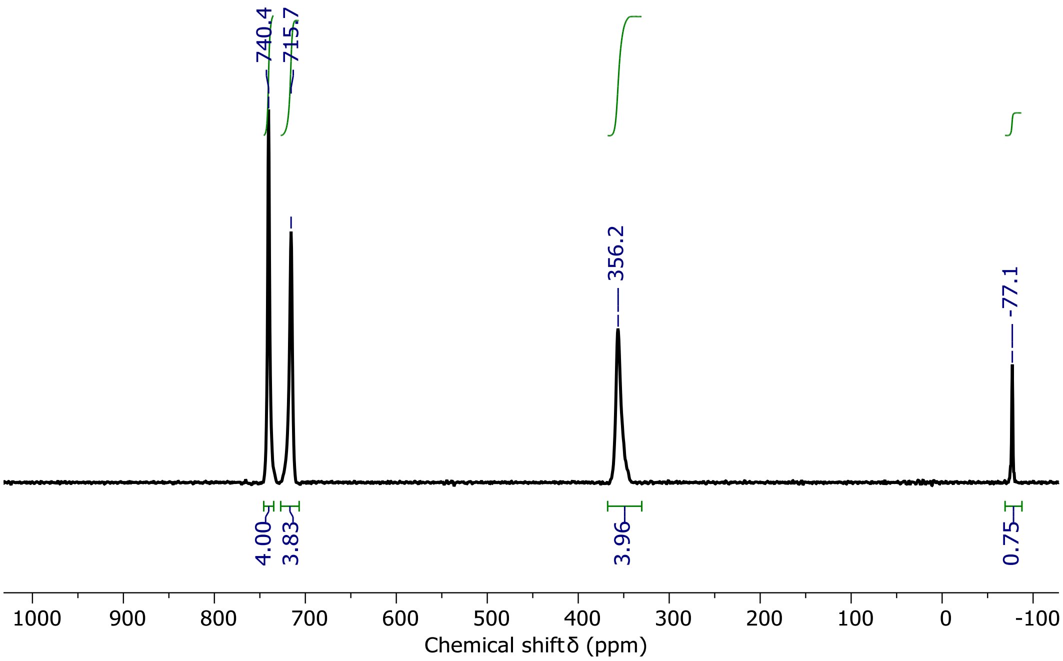

Figure S9.^{17}O NMR spectrum (500 MHz) of $(TBA)_3[Ce\{W_4O_{13}(OMe)_4MoNO\}_2]$ (**2-Ce(W$_4$Mo)$_2$**) in CD_3CN at 19.3 °C.

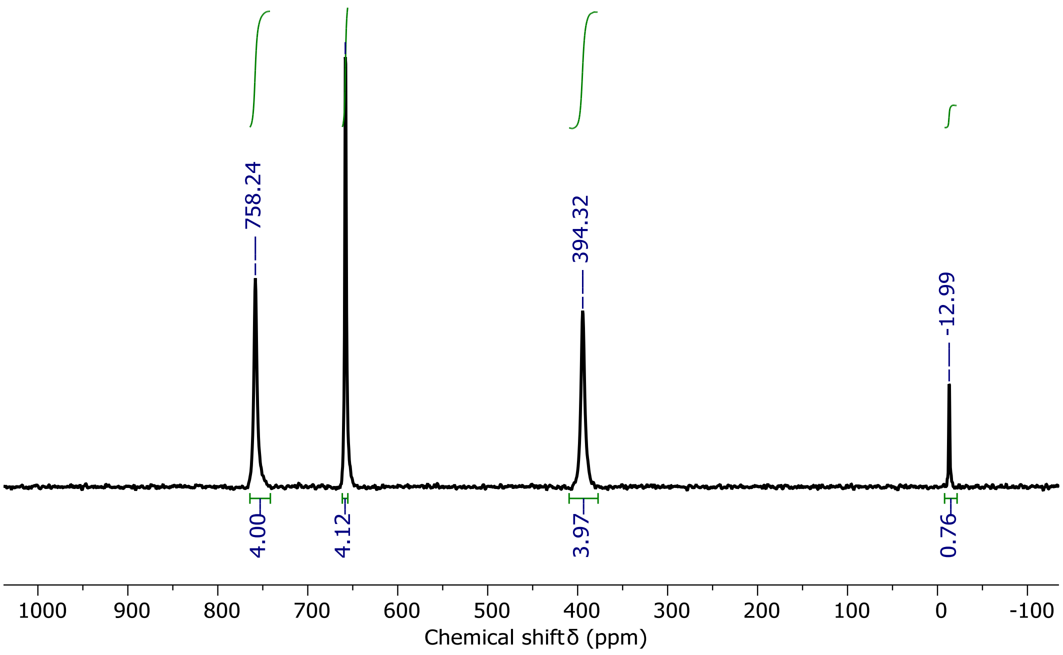

Figure S10.^{17}O NMR spectrum (500 MHz) of $(TBA)_2[Ce\{W_4O_{13}(OMe)_4MoNO\}_2]$ (**3-Ce(W$_4$Mo)$_2$**) in CD_2Cl_2.

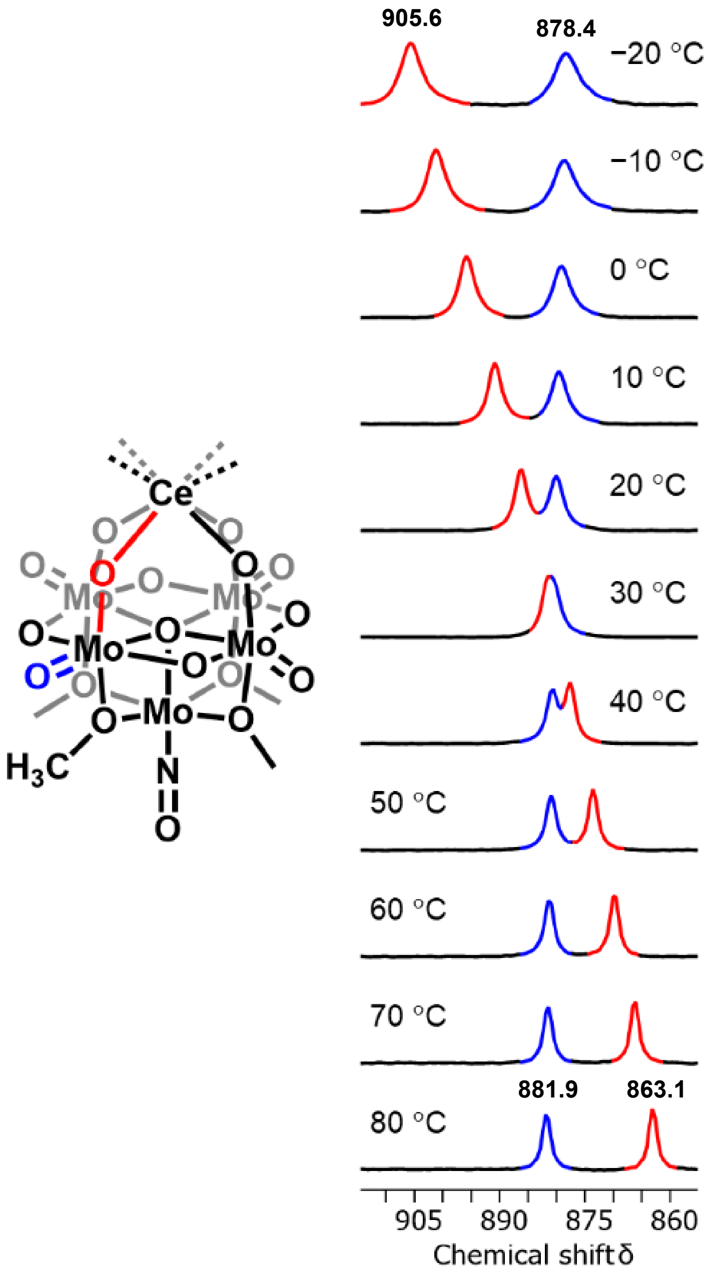

Figure S11. Variable temperature ^{17}O NMR spectra (500 MHz) of $(TBA)_3[Ce\{Mo_5O_{13}(OMe)_4NO\}_2]$ (**2-Ce(Mo$_5$)$_2$**) in CD_3CN. Spectra show the distinct upfield shifting of the Ce-O-Mo signal as the temperature was varied between −20 °C and 80 °C.

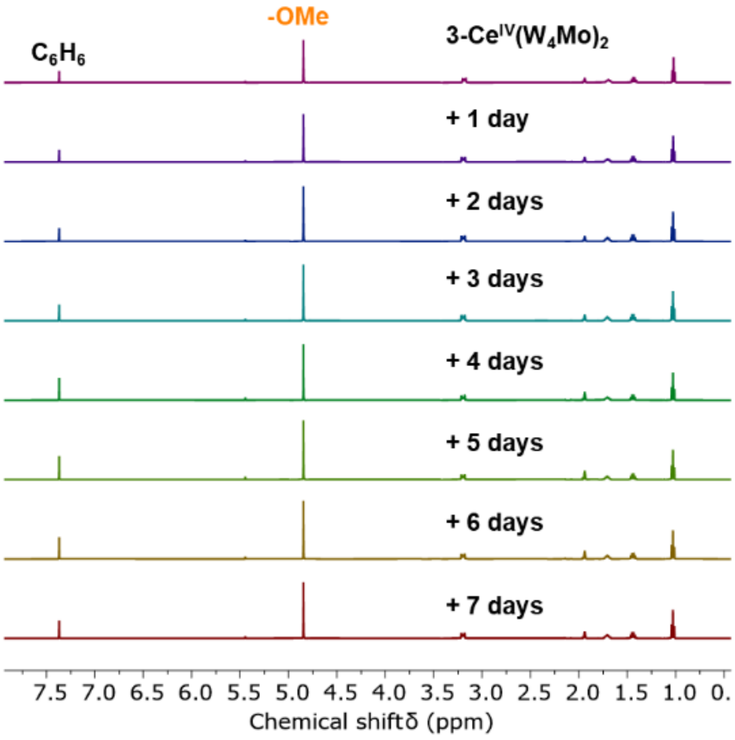

Time	C_6H_6 Integration	-OMe Integration	%Ce(W_4Mo)$_2$ remaining
Fresh	1.00	6.97	100%
1 day	1.00	6.40	92%
2 days	1.00	5.99	86%
3 days	1.00	5.35	77%
4 days	1.00	4.78	69%
5 days	1.00	4.46	64%
6 days	1.00	4.17	60%
7 days	1.00	4.05	58%

Figure S12. ^{1}H NMR of **3-Ce(W_4Mo)$_2$** in CD_3CN with an internal C_6H_6 standard. The sample was prepared under inert atmosphere and sealed in a J-Young tube. A ^{1}H NMR spectrum was recorded every day for seven days and the amount **of 3-Ce(W_4Mo)$_2$** remaining was determined from the relative size of the -OMe peak of the complex compared to that of the internal standard.

S3. UV-Vis-NIR spectra

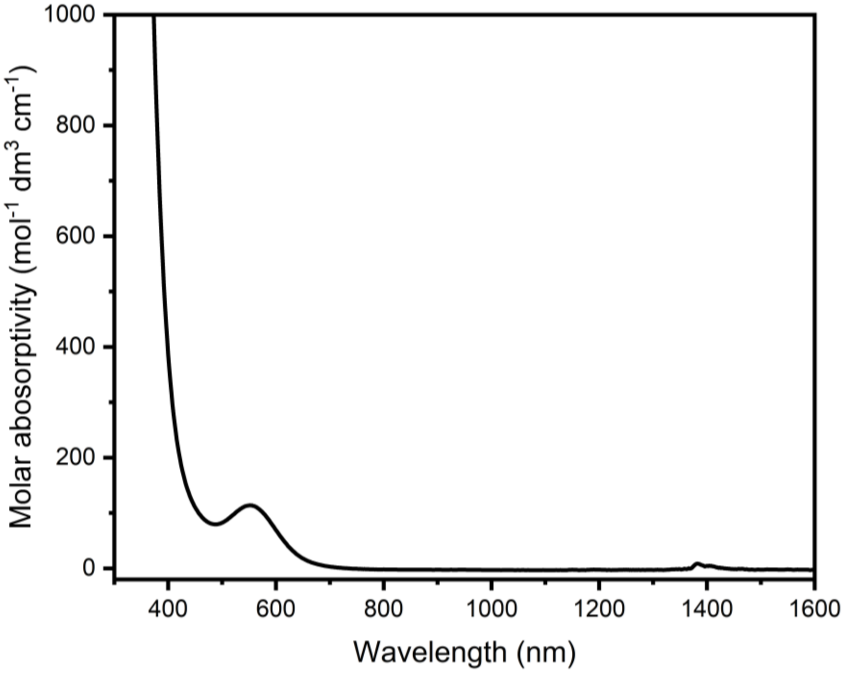

Figure S13. UV-Vis-NIR spectrum of a 1 mM solution $(TBA)_3[Ce\{Mo_5O_{13}(OMe)_4NO\}_2]$ (**2-Ce(Mo$_5$)$_2$**). The spectrum was acquired at room temperature (19.5 °C) in in MeCN.

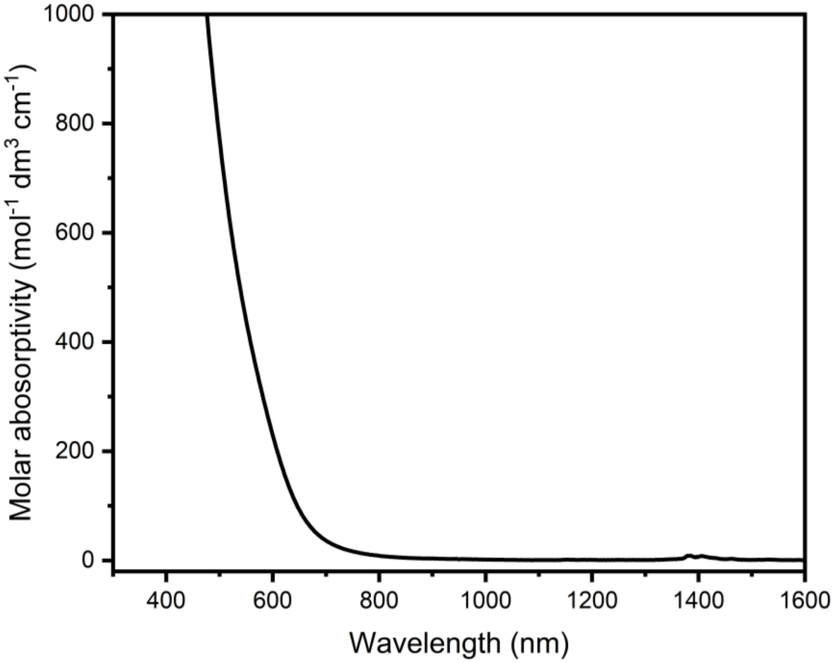

Figure S14. UV-Vis-NIR spectrum of a 1 mM solution $(TBA)_2[Ce\{Mo_5O_{13}(OMe)_4NO\}_2]$ (**3-Ce(Mo$_5$)$_2$**) obtained by chemical oxidation of **2-Ce(Mo$_5$)$_2$** with $[N(C_6H_4Br\text{-}4)_3][SbCl_6]$. Spectrum was acquired at room temperature (19.5 °C) in MeCN.

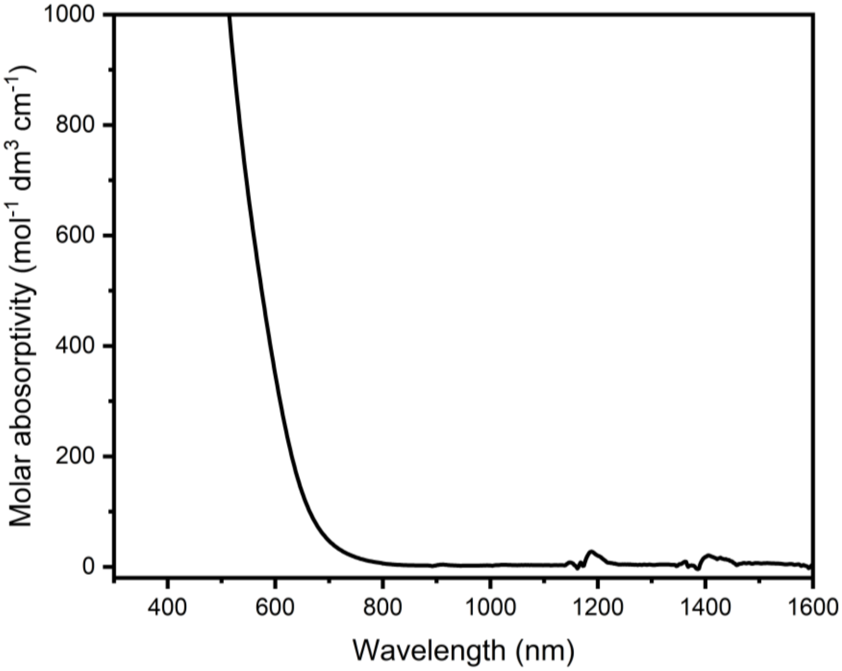

Figure S15. UV-Vis-NIR spectrum of a 1 mM solution $(TBA)_2[Ce\{Mo_5O_{13}(OMe)_4NO\}_2]$ (**3-Ce(Mo$_5$)$_2$**) obtained by electrochemical oxidation of **2-Ce(Mo$_5$)$_2$**. The spectrum was acquired at room temperature (19.5 °C) in a 0.1 M solution of TBA(PF_6) in MeCN.

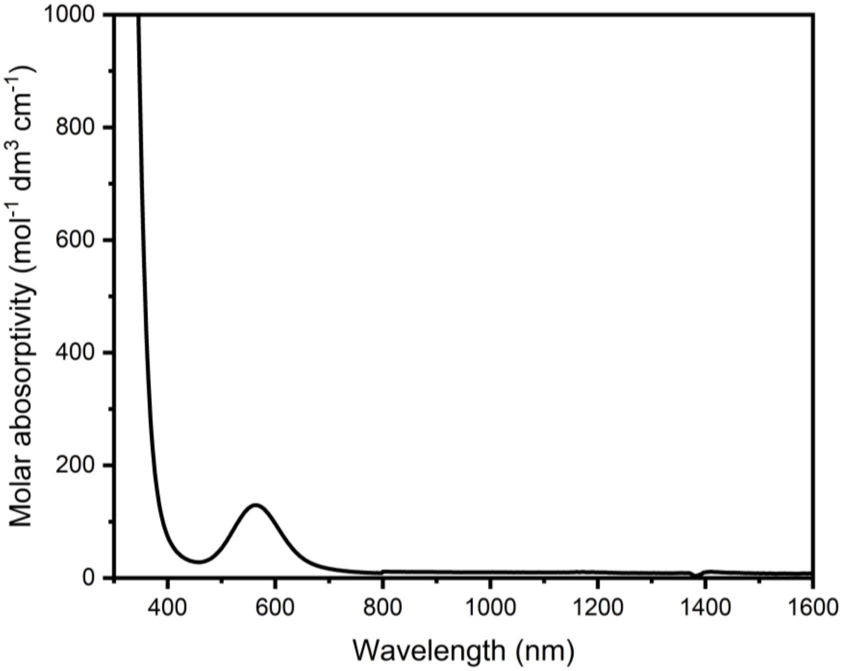

Figure S16. UV-Vis-NIR spectrum of a 1 mM solution $(TBA)_3[Ce\{W_4O_{13}(OMe)_4MoNO\}_2]$ (**2-Ce(W$_4$Mo)$_2$**). The spectrum was acquired at room temperature (19.5 °C) in in MeCN.

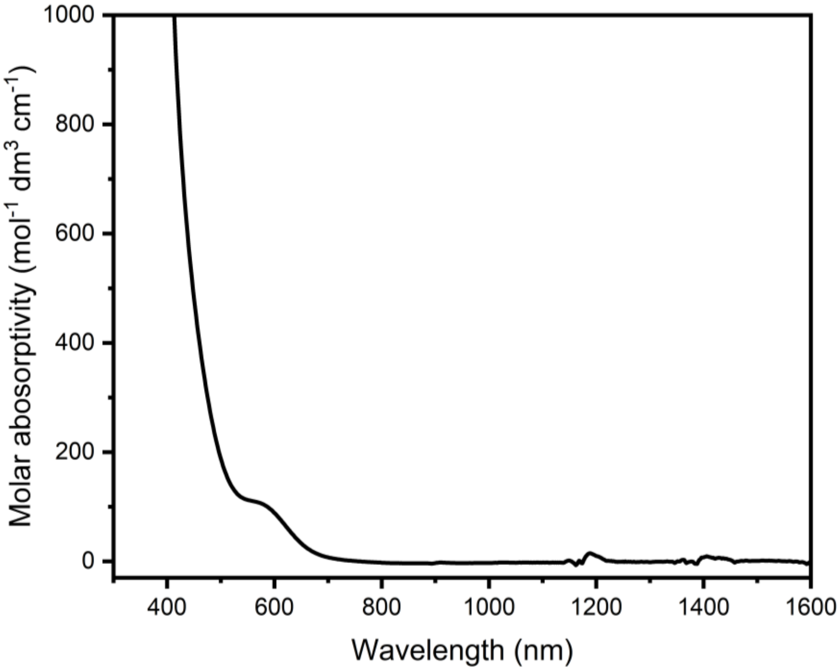

Figure S17. UV-Vis-NIR spectrum of a 1 mM solution $(TBA)_2[Ce\{W_4O_{13}(OMe)_4MoNO\}_2]$ (**3-Ce(W$_4$Mo)$_2$**) obtained by electrochemical oxidation of **2-Ce(W$_4$Mo)$_2$**. The spectrum was acquired at room temperature (19.5 °C) in a 0.1 M solution of TBA(PF_6) in MeCN.

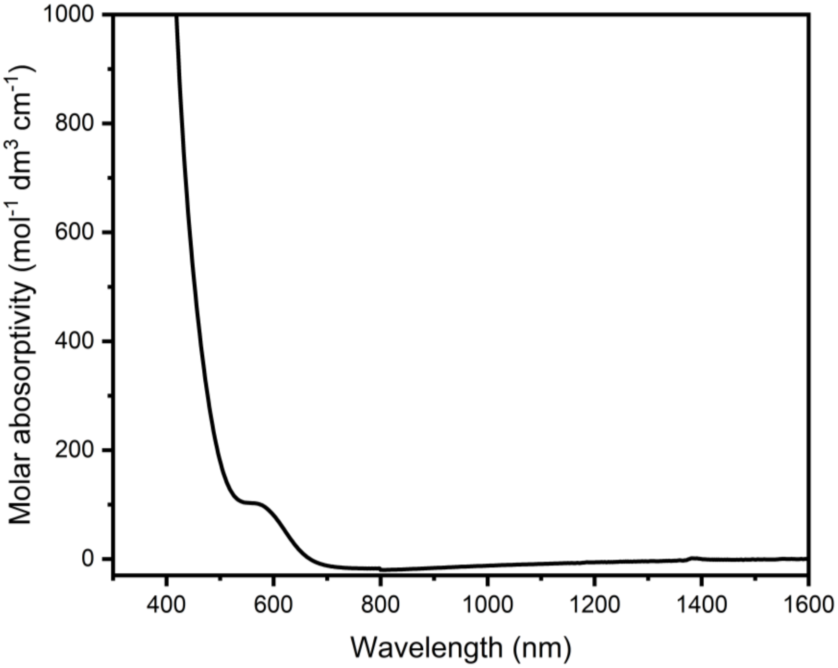

Figure S18. UV-Vis-NIR spectrum of a 1 mM solution $(TBA)_2[Ce\{W_4O_{13}(OMe)_4MoNO\}_2]$ (**3-Ce(W$_4$Mo)$_2$**) obtained by chemical oxidation of **2-Ce(W$_4$Mo)$_2$** with $[N(C_6H_4Br\text{-}4)_3][SbCl_6]$. Spectrum was acquired at room temperature (19.5 °C) in MeCN.

S4. Electrochemistry

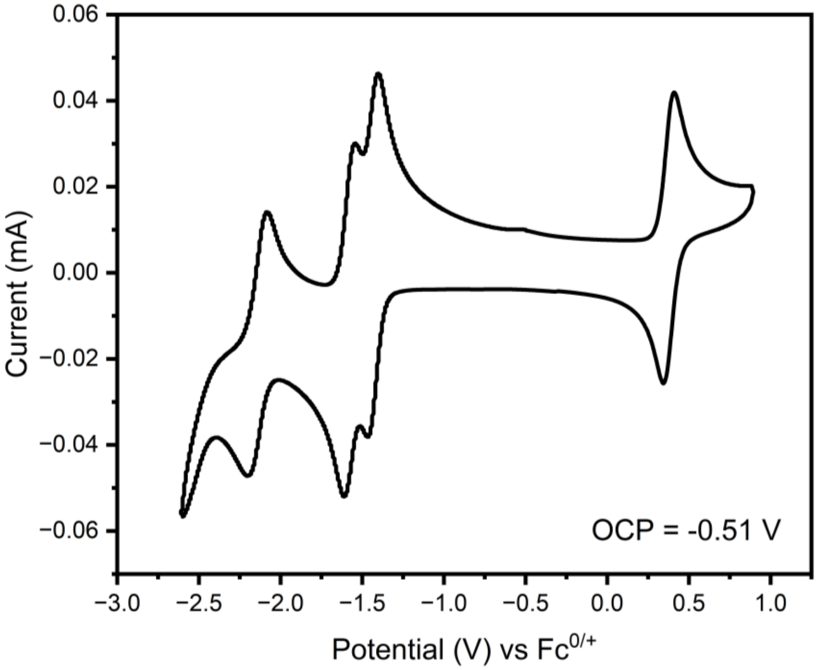

Figure S19. Cyclic voltammogram of a 1 mM solution $(TBA)_3[Ce\{Mo_5O_{13}(OMe)_4NO\}_2]$ (**2-Ce(Mo$_5$)$_2$**). The voltammogram was acquired in a 0.1 M solution of $TBA(PF_6)$ in MeCN at room temperature (19.5 °C).

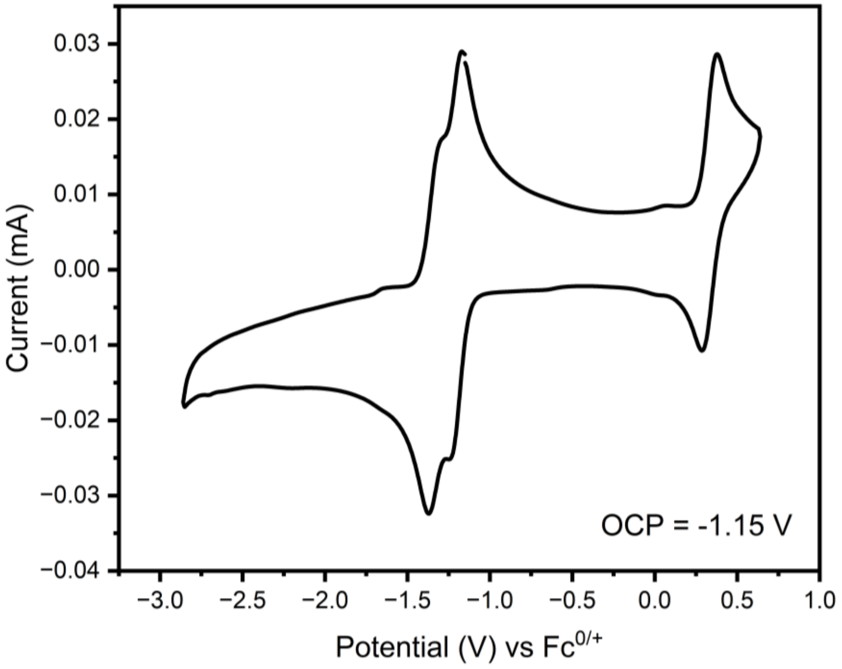

Figure S20. Cyclic voltammogram of a 1 mM solution $(TBA)_3[Ce\{W_4O_{13}(OMe)_4MoNO\}_2]$ (**2-Ce(W$_4$Mo)$_2$**). The voltammogram was acquired in a 0.1 M solution of $TBA(PF_6)$ in MeCN at room temperature (19.5 °C).

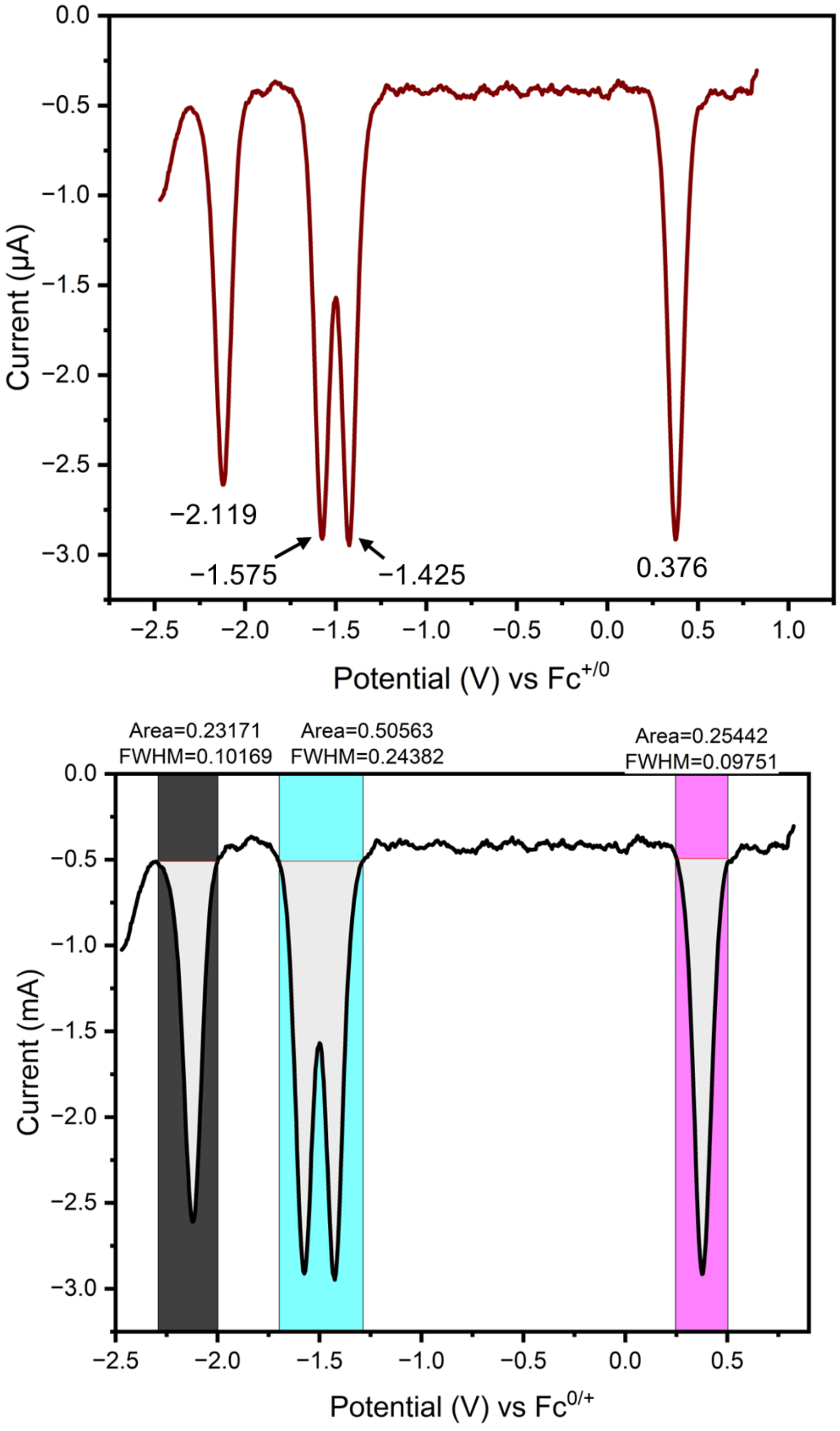

Figure S21. Differential pulse voltammogram (DPV) of a 1 mM solution $(TBA)_3[Ce\{Mo_5O_{13}(OMe)_4NO\}_2]$ (**2-Ce(W$_4$Mo)$_2$**). The voltammogram was acquired in a 0.1 M solution of TBA(PF_6) in MeCN at room temperature (19.5 °C). Pulse height = 2.5 mV, Pulse width = 100 ms, Step height = −5 mV, Step time = 500 ms. The scan started at 0.825 V vs $Fc^{+/0}$.

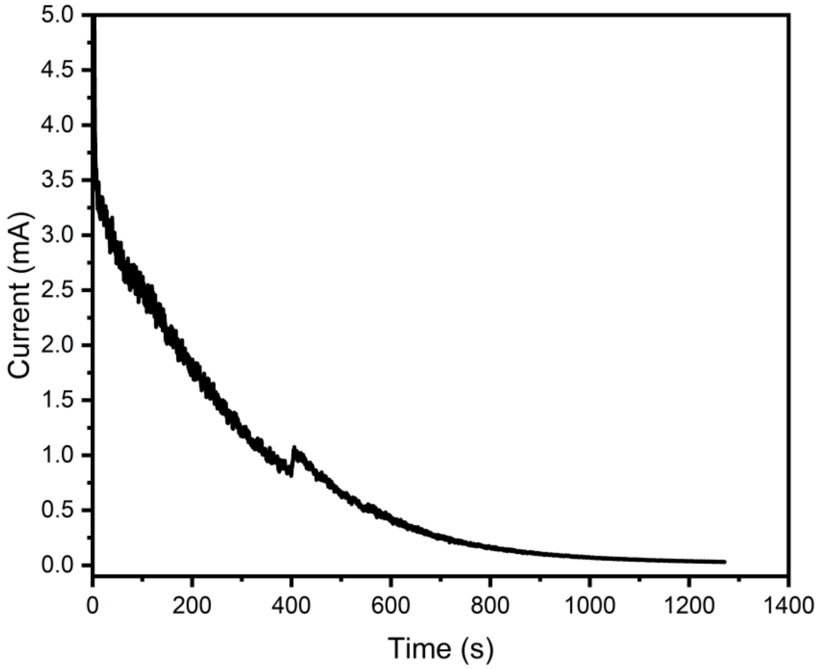

Figure S22. Controlled potential electrolysis of a 1 mM solution $(TBA)_3[Ce\{Mo_5O_{13}(OMe)_4NO\}_2]$ (**2-Ce(Mo$_5$)$_2$**) at 0.69 V vs $Fc^{+/0}$ in 0.1 M $TBA(PF_6)$/MeCN. The experiment was performed at room temperature (19.5 °C).

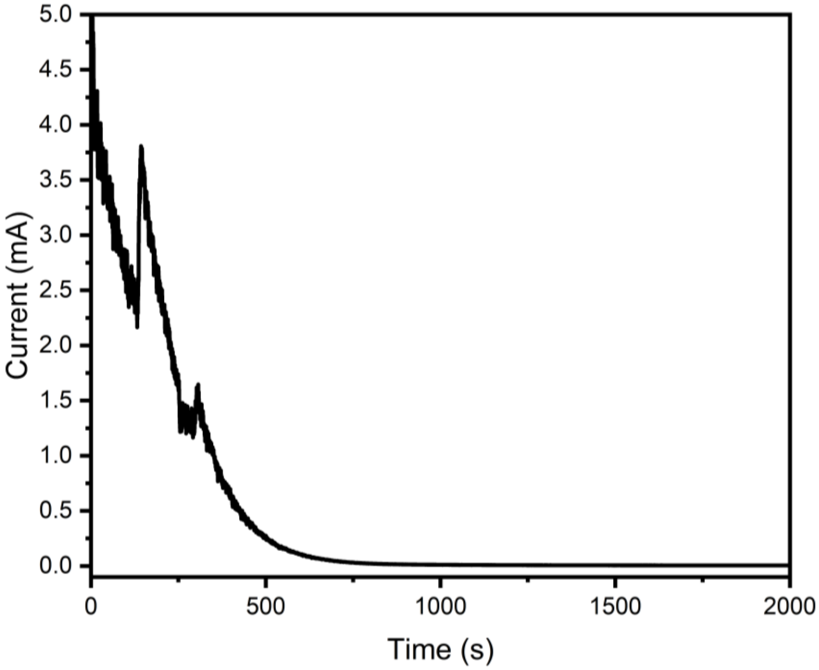

Figure S23. Controlled potential electrolysis of a 1 mM solution $(TBA)_3[Ce\{W_4O_{13}(OMe)_4MoNO\}_2]$ (**2-Ce(W$_4$Mo)$_2$**) at 0.70 V vs $Fc^{+/0}$ in 0.1 M $TBA(PF_6)$/MeCN. The experiment was performed at room temperature (19.5 °C).

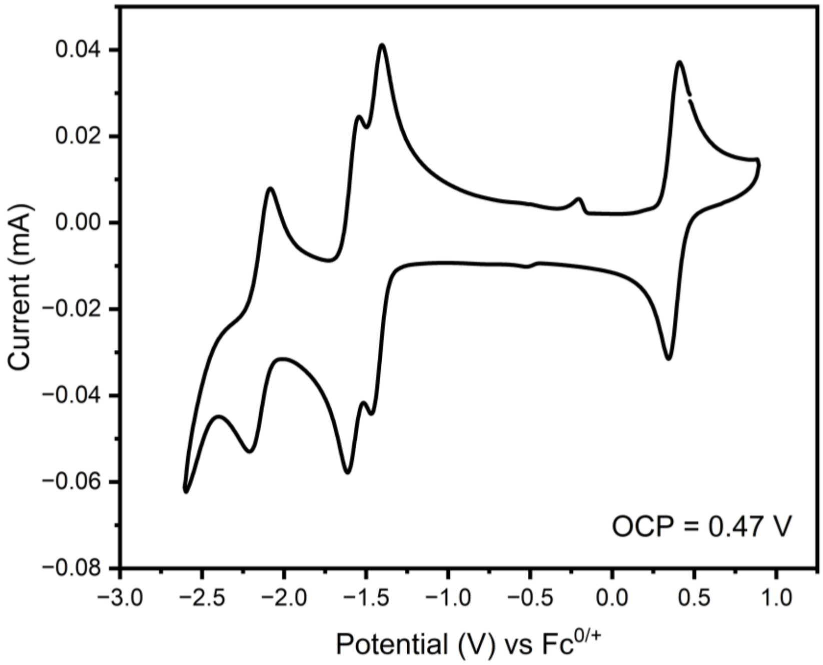

Figure S24. Cyclic voltammogram obtained after controlled potential electrolysis of a 1 mM solution $(TBA)_3[Ce\{Mo_5O_{13}(OMe)_4NO\}_2]$ (**2-Ce(Mo$_5$)$_2$**) at 0.69 V vs $Fc^{+/0}$ in 0.1 M $TBA(PF_6)$/MeCN. The voltammogram was acquired at room temperature (19.5 °C).

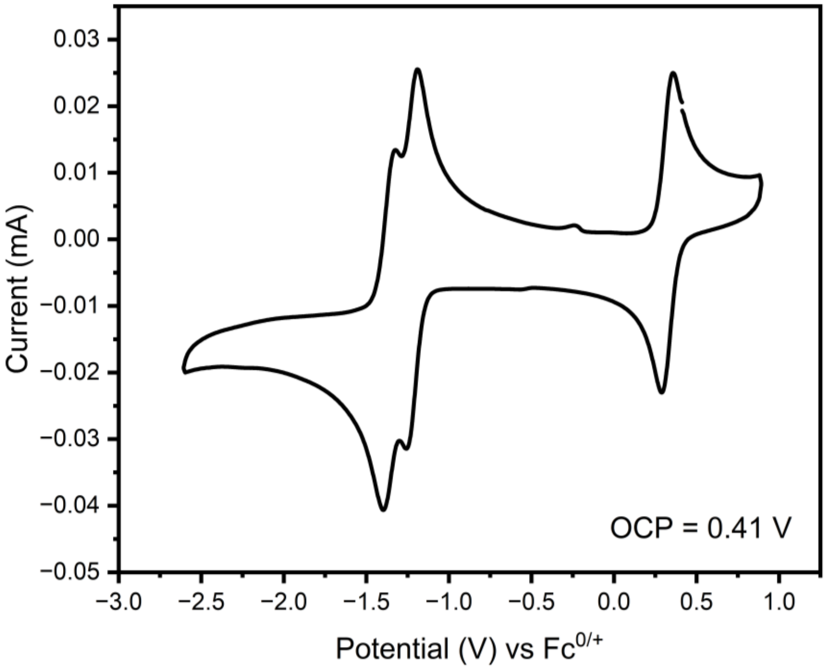

Figure S25. Cyclic voltammogram obtained after controlled potential electrolysis of a 1 mM solution $(TBA)_3[Ce\{W_4O_{13}(OMe)_4MoNO\}_2]$ (**2-Ce(W$_4$Mo)$_2$**) at 0.70 V vs $Fc^{+/0}$ in 0.1 M $TBA(PF_6)$/MeCN. The voltammogram was acquired at room temperature (19.5 °C).

S5. Single Crystal X-ray Diffraction

Additional refinement details

For **2-Ce(Mo$_5$)$_2$**, the asymmetric unit contains one trianionic $CeMo_{10}$ cluster, three tetrabutylammonium cations, and one diethyl ether solvent molecule, all in general positions. Butyl group C53-C56 and the diethyl ether solvent molecule are modeled as disordered over two positions each (0.63:0.37). The common disorder ratio is due to their adjacency.

For **2-Ce(W$_4$Mo)$_2$**, the asymmetric unit contains one trianionic $CeMo_2W_8$ cluster, three tetrabutylammonium cations, and three ethanol solvent molecules, all in general positions. Methoxy ligands O11-C1 and O24-C6 are modeled as disordered over two positions each (0.71:0.29 and 0.57:0.43, respectively). The majority of tetrabutylammonium cation N5/C41-C56 is modeled as disordered over two positions (0.50:0.50 exactly, due to the disorder extending across a crystallographic inversion center). Methanol solvent molecule O39-C59 is modeled as disordered over two positions (0.69:0.31). The O-H hydrogen atom on adjacent methanol solvent molecule O38-C58 is modeled as disordered to account for the O39-C59 disorder.

For **3-Ce(Mo$_5$)$_2$**, the asymmetric unit contains one dianionic $CeMo_{10}$ cluster, two tetrabutylammonium cations, and one acetonitrile solvent molecule in general positions, and one-half of a diethyl ether solvent molecule adjacent to a crystallographic inversion center. Two methoxy ligands are modeled as disordered over two positions each: O13-C3, 0.74:0.26, O25-C7, 0.72:0.28. Butyl groups C13-C16 and C21-C24 are modeled as disordered over two positions each (0.68:0.32 and 0.66:0.34, respectively). Cation N4/C25-C40 is modeled as disorder over two positions (0.53:0.47). The diethyl ether solvent molecule is modeled as disordered over the nearby crystallographic inversion center (0.50:0.50).

For **3-Ce(W$_4$Mo)$_2$**, the asymmetric unit contains one dianionic CeW_8Mo_2 cluster, two tetrabutylammonium cations, and one acetonitrile solvent molecule in general positions, and one-half of a diethyl ether solvent molecule adjacent to a crystallographic inversion center. Methoxy ligand O13-C3 is modeled as disordered over two positions (0.74:0.26). Butyl groups C17-C20 and C21-C24 are modeled as disordered over two positions each (both 0.61:0.39). Tetrabutylammonium cation N4/C21-C40 is modeled as disordered over two positions (0.58:0.42). The diethyl ether solvent molecule is modeled as disordered over a crystallographic inversion center (0.50:0.50). **3-Ce(Mo$_5$)$_2$** and **3-Ce(W$_4$Mo)$_2$** are isomorphous.

Table S1. Crystal data and structure refinement for **2-Ce(Mo$_5$)$_2$**.

Empirical formula	$C_{60}H_{142}CeMo_{10}N_5O_{37}$	
Formula weight	2625.30	
Temperature	100.00(10) K	
Wavelength	1.54184 Å	
Crystal system	monoclinic	
Space group	$P2_1/c$	
Unit cell dimensions	*a* = 23.92560(10) Å	α = 90°
	b = 22.61610(10) Å	β = 108.1910(10)°
	c = 18.27070(10) Å	γ = 90°
Volume	9392.23(9) Å^3	
Z	4	
Density (calculated)	1.857 Mg/m^3	
Absorption coefficient	14.876 mm^{-1}	
F(000)	5244	
Crystal color, morphology	violet, block	
Crystal size	0.197 x 0.074 x 0.074 mm^3	
Theta range for data collection	2.756 to 80.624°	
Index ranges	-29 £ *h* £ 30, -27 £ *k* £ 28, -23 £ *l* £ 22	
Reflections collected	162835	
Independent reflections	20285 [*R*(int) = 0.0475]	
Observed reflections	19589	
Completeness to theta = 74.504°	100.0%	
Absorption correction	Multi-scan	
Max. and min. transmission	1.00000 and 0.33105	
Refinement method	Full-matrix least-squares on F^2	
Data / restraints / parameters	20285 / 96 / 1112	
Goodness-of-fit on F^2	1.066	
Final *R* indices [*I*>2sigma(*I*)]	*R*1 = 0.0263, *wR*2 = 0.0660	
R indices (all data)	*R*1 = 0.0274, *wR*2 = 0.0667	
Largest diff. peak and hole	0.795 and -0.880 e.Å^{-3}	

Table S2. Crystal data and structure refinement for **2-Ce(W$_4$Mo)$_2$**.

Empirical formula	$C_{59}H_{144}CeMo_2N_5O_{39}W_8$	
Formula weight	3350.58	
Temperature	100.01(10) K	
Wavelength	1.54184 Å	
Crystal system	triclinic	
Space group	*P*-1	
Unit cell dimensions	*a* = 13.11989(7) Å	α = 77.0060(6)°
	b = 14.37437(10) Å	β = 89.2214(5)°
	c = 25.9380(2) Å	γ = 75.4995(5)°
Volume	4609.84(6) Å^3	
Z	2	
Density (calculated)	2.414 Mg/m^3	
Absorption coefficient	24.444 mm^{-1}	
F(000)	3158	
Crystal color, morphology	violet, block	
Crystal size	0.137 x 0.077 x 0.027 mm^3	
Theta range for data collection	3.262 to 80.330°	
Index ranges	-16 £ *h* £ 16, -18 £ *k* £ 18, -29 £ *l* £ 33	
Reflections collected	153233	
Independent reflections	19810 [*R*(int) = 0.0439]	
Observed reflections	19202	
Completeness to theta = 74.504°	99.7%	
Absorption correction	Multi-scan	
Max. and min. transmission	1.00000 and 0.29542	
Refinement method	Full-matrix least-squares on F^2	
Data / restraints / parameters	19810 / 360 / 1211	
Goodness-of-fit on F^2	1.108	
Final *R* indices [*I*>2sigma(*I*)]	*R*1 = 0.0287, *wR*2 = 0.0627	
R indices (all data)	*R*1 = 0.0298, *wR*2 = 0.0632	
Largest diff. peak and hole	1.665 and -1.759 e.Å^{-3}	

Table S3. Crystal data and structure refinement for **3-Ce(Mo$_5$)$_2$**.

Empirical formula	$C_{44}H_{104}CeMo_{10}N_5O_{36.5}$	
Formula weight	2386.84	
Temperature	100.00(10) K	
Wavelength	1.54184 Å	
Crystal system	monoclinic	
Space group	$P2_1/c$	
Unit cell dimensions	*a* = 21.78196(10) Å	α = 90°
	b = 15.03091(7) Å	β = 92.8717(4)°
	c = 24.10430(13) Å	γ = 90°
Volume	7881.90(7) Å^3	
Z	4	
Density (calculated)	2.011 Mg/m^3	
Absorption coefficient	17.645 mm^{-1}	
F(000)	4692	
Crystal color, morphology	red, needle	
Crystal size	0.212 x 0.097 x 0.07 mm^3	
Theta range for data collection	3.467 to 80.125°	
Index ranges	-27 £ *h* £ 27, -17 £ *k* £ 19, -30 £ *l* £ 30	
Reflections collected	134413	
Independent reflections	16997 [*R*(int) = 0.0463]	
Observed reflections	16179	
Completeness to theta = 74.504°	100.0%	
Absorption correction	Multi-scan	
Max. and min. transmission	1.00000 and 0.12773	
Refinement method	Full-matrix least-squares on F^2	
Data / restraints / parameters	16997 / 740 / 1131	
Goodness-of-fit on F^2	1.112	
Final *R* indices [*I*>2sigma(*I*)]	*R*1 = 0.0481, *wR*2 = 0.1091	
R indices (all data)	*R*1 = 0.0503, *wR*2 = 0.1102	
Largest diff. peak and hole	2.579 and -2.231 e.Å^{-3}	

Table S4. Crystal data and structure refinement for **3-Ce(W$_4$Mo)$_2$**.

Empirical formula	$C_{44}H_{104}CeMo_2N_5O_{36.5}W_8$	
Formula weight	3090.12	
Temperature	173.00(10) K	
Wavelength	1.54184 Å	
Crystal system	monoclinic	
Space group	$P2_1/c$	
Unit cell dimensions	*a* = 21.8546(2) Å	α = 90°
	b = 15.10070(10) Å	β = 92.8090(10)°
	c = 24.2678(2) Å	γ = 90°
Volume	7999.23(11) Å^3	
Z	4	
Density (calculated)	2.566 Mg/m^3	
Absorption coefficient	28.067 mm^{-1}	
F(000)	5716	
Crystal color, morphology	orange-blue, block	
Crystal size	0.106 x 0.096 x 0.058 mm^3	
Theta range for data collection	3.448 to 80.763°	
Index ranges	-27 £ *h* £ 27, -19 £ *k* £ 18, -28 £ *l* £ 31	
Reflections collected	137249	
Independent reflections	17176 [*R*(int) = 0.0541]	
Observed reflections	15797	
Completeness to theta = 74.504°	99.8%	
Absorption correction	Multi-scan	
Max. and min. transmission	1.00000 and 0.66058	
Refinement method	Full-matrix least-squares on F^2	
Data / restraints / parameters	17176 / 752 / 1126	
Goodness-of-fit on F^2	1.048	
Final *R* indices [*I*>2sigma(*I*)]	*R*1 = 0.0380, *wR*2 = 0.0830	
R indices (all data)	*R*1 = 0.0417, *wR*2 = 0.0848	
Largest diff. peak and hole	2.446 and -2.797 e.Å^{-3}	

Table S5: Average bond length data for the structures discussed. All values in Å. A schematic is given below to highlight bond assignments.

	**2-Ce(Mo$_5$)$_2$**	**3-Ce(Mo$_5$)$_2$**		**2-Ce(W$_4$Mo)$_2$**	**3-Ce(W$_4$Mo)$_2$**
**Ce-O**	2.483	2.343	**Ce-O**	2.483	2.352
**Mo-O-Mo**	1.919	1.913	**W-O-W**	1.917	1.915
**Mo=O**	1.699	1.689	**W=O**	1.719	1.700
**Mo-O-Ce**	1.754	1.774	**W-O-Ce**	1.767	1.785
**Mo-O(μ$_5$)**	2.331	2.324	**W-O(μ$_5$)**	2.342	2.333
**Mo-O(μ$_5$) (ax)**	2.134	2.117	**Mo-O(μ$_5$)**	2.133	2.118
**Mo-O (eq)**	2.009	2.007	**Mo-O (eq)**	2.004	2.008
**Mo-O (ax)**	2.242	2.201	**W-O (ax)**	2.227	2.192
**M$_5$O- μ$_5$O**	7.095	6.816	**M$_5$O- μ$_5$O**	7.073	6.849
**O-O**	3.245	2.976	**O-O**	3.214	2.995
**Mo-NO**	1.775	1.769	**Mo-NO**	1.771	1.764

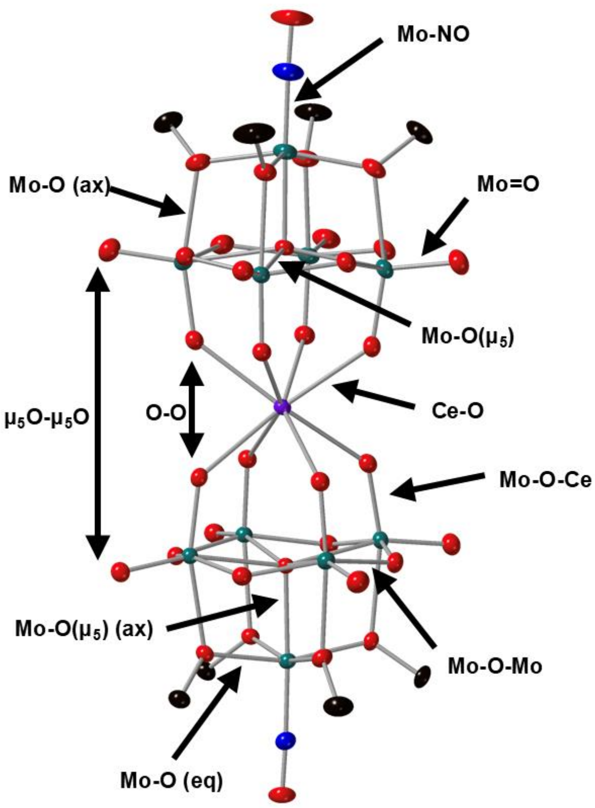

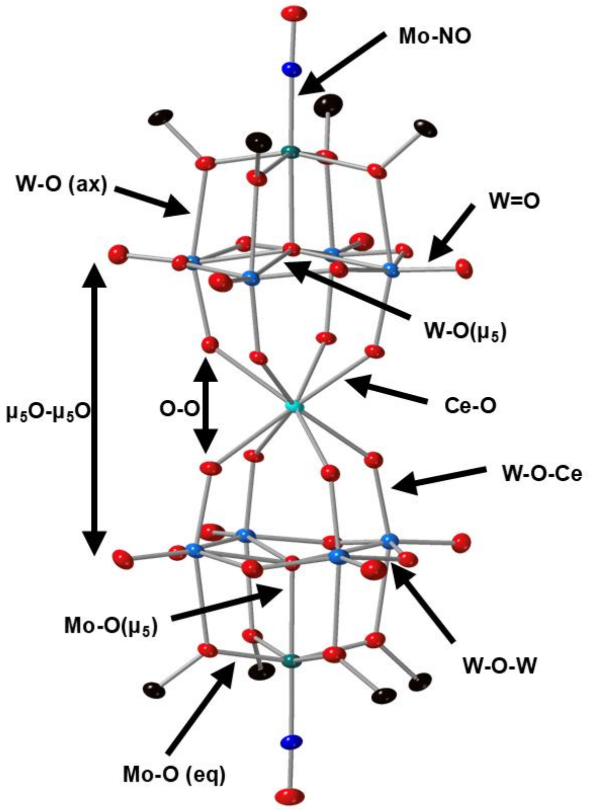

Table S6. Results for square antiprismatic continuous shape measurement (CShM) calculations using program *SHAPE*. Positions in Cartesian coordinates (x,y,z).

Structure	**2-Ce(Mo$_5$)$_2$**		
Ce	4.3860	10.9799	4.1858
O	5.8169	12.3821	2.7453
O	4.7451	9.7778	2.0671
O	5.4782	8.7554	4.6367
O	6.5567	11.3273	5.3307
O	3.5060	10.4950	6.4416
O	4.0626	13.1492	5.3796
O	2.7903	12.4223	2.9209
O	2.2185	9.8068	3.9409

SAPR-8	Ideal structure	CShM =	0.93012	
Ce	M	4.3956	11.0106	4.1832
O	L1	5.8262	12.5253	2.8538
O	L2	4.5661	9.9496	1.9576
O	L3	5.1962	8.7141	4.6225
O	L4	6.4563	11.2898	5.5187
O	L8	3.7255	10.5280	6.5126
O	L5	4.1710	13.2229	5.2619
O	L6	2.8344	12.2752	2.7438
O	L7	2.3889	9.5803	3.9945

Structure	**2-Ce(W$_4$Mo)$_2$**		
Ce	6.2981	12.0190	19.0521
O	4.8032	12.7778	17.2065
O	7.4517	11.6739	16.8575
O	8.3856	13.3394	19.0579
O	5.7332	14.4439	19.3998
O	7.9096	10.2458	19.6666
O	6.6703	12.1006	21.5049
O	4.1274	11.7356	20.1996
O	5.3799	9.8503	18.3424

SAPR-8	Ideal structure	CShM =	0.42435	
Ce	M	6.3066	12.0207	19.0319
O	L1	4.6944	12.6644	17.2634
O	L2	7.4729	11.5607	16.8943
O	L3	8.4501	13.2547	19.1855
O	L4	5.6717	14.3584	19.5546
O	L7	8.0055	10.3014	19.5785
O	L8	6.7319	12.2797	21.4595
O	L5	4.0762	11.8623	20.1004
O	L6	5.3498	9.8840	18.2193

Structure	**3-Ce(Mo$_5$)$_2$**		
Ce	15.4290	5.8410	18.7289
O	13.6753	5.6988	17.1405
O	14.0974	7.7761	18.9426
O	14.6504	5.9319	20.9408
O	14.2339	3.8678	19.1451
O	17.0745	4.5852	19.7949
O	16.3855	4.4699	17.1202
O	16.2358	7.2144	17.0271
O	16.9282	7.3483	19.7118

SAPR-8	Ideal structure	CShM =	0.20960	
Ce	M	15.4122	5.8593	18.7280
O	L1	13.7593	5.7159	17.0777
O	L2	14.1645	7.8277	18.9399
O	L3	14.7492	5.9242	20.9713
O	L4	14.3439	3.8124	19.1091
O	L8	16.9836	4.5525	19.8679
O	L5	16.2836	4.4053	17.1147
O	L6	16.1568	7.2445	16.9951
O	L7	16.8568	7.3918	19.7482

Structure	**3-Ce(W$_4$Mo)$_2$**		
Ce	4.5561	5.8766	18.8397
O	2.8043	5.7383	17.2388
O	3.2260	7.8140	19.0678
O	3.7768	5.9396	21.0530
O	3.3581	3.8911	19.2355
O	5.3915	7.2606	17.1406
O	5.5378	4.4980	17.2271
O	6.2134	4.6075	19.9154
O	6.0711	7.3829	19.8313

SAPR-8	Ideal structure	CShM =	0.22048	
Ce	M	4.5483	5.8898	18.8388
O	L1	2.8949	5.7561	17.1736
O	L2	3.2953	7.8663	19.0577
O	L3	3.8731	5.9426	21.0896
O	L4	3.4728	3.8324	19.2055
O	L6	5.3041	7.2906	17.1093
O	L5	5.4296	4.4382	17.2138
O	L8	6.1213	4.5701	19.9828
O	L7	5.9958	7.4225	19.8782

S6. Computational calculations

Table S7. The five most significant electronic transitions below 400 nm, obtained with TDDFT for **3-Ce(Mo$_5$)$_2$**. These transitions are the ones contributing to the broad absorption band observed below 400 nm in the experimental UV-Vis spectrum. All of these transitions involve a LMCT process from orbitals localized on the POM cage to unoccupied 4f orbitals localized on Ce, and a lesser LLCT process from orbitals on the Mo-NO unit to the ligand cage. Differently from **3-Ce(W$_4$Mo)$_2$**, the dominant transitions fall in the 385 nm range, while other types of LMCT present at lower energy (~350 nm) contribute less to the overall band.

**Transition Number**	**Transition Wavelength**	**Contribution (%)**
63	385.8	12.2
74	378.6	11.6
67	384.0	10.7
50	392.1	9.0
62	386.1	8.8

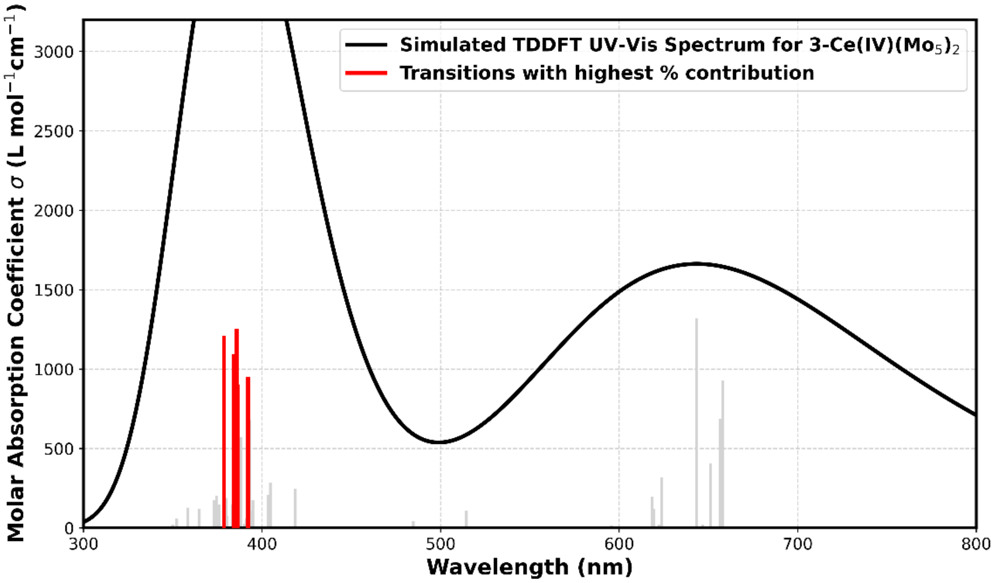

Figure S26. Simulated TDDFT UV-Vis spectrum for **3-Ce(Mo$_5$)$_2$**, highlighting the five most important transitions contributing to the broad band below 400 nm. The orbitals involved in a representative transition (63) are shown in the following figures.

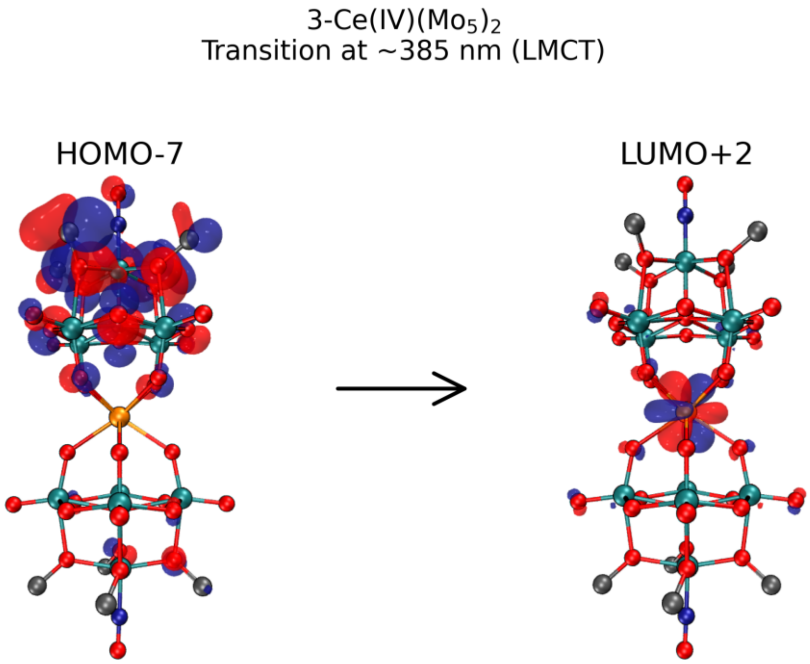

Figure S27: Representative molecular orbitals involved in the dominant LMCT transition for **3-Ce(Mo$_5$)$_2$**. This is the dominant contribution to the absorption band below 400 nm (Figure S1), and it involves transfer from orbitals localized on the POM cage to the unoccupied 4f orbitals on Ce.

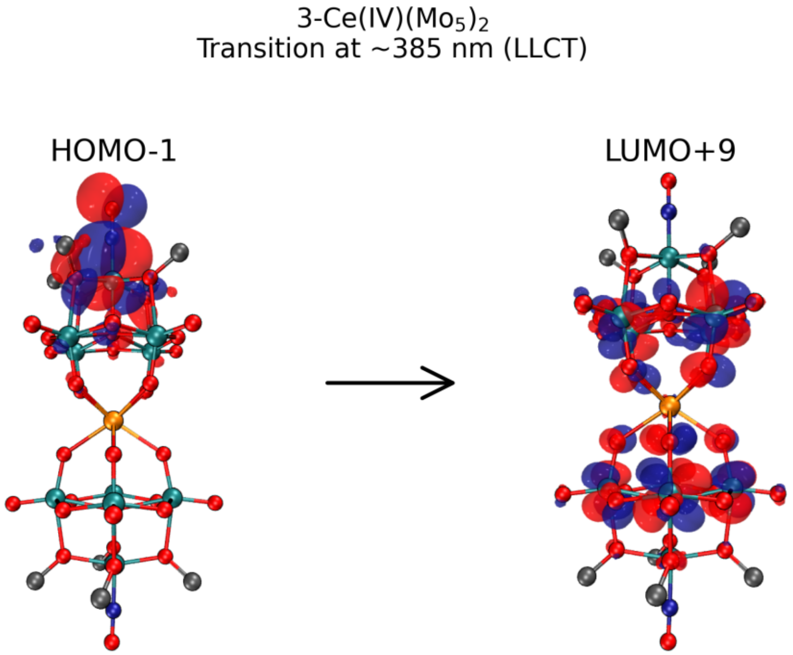

Figure S28. Representative molecular orbitals for the secondary LLCT process contributing to the absorption band of **3-Ce(Mo$_5$)$_2$**. This less intense transition involves orbitals localized on the {MoNO} unit to the main POM cage.

Table S8. The five most significant electronic transitions around 640nm, obtained with TDDFT for **3-Ce(Mo$_5$)$_2$**. These transitions are the ones contributing to the absorption band observed around 640nm in the experimental UV-Vis spectrum. All these transitions involve a LMCT process from orbitals localized on the {Mo-NO}$_4$ to unoccupied 4f orbitals localized on Ce. This type of transition is consistent with our previous paper, with the difference in the LUMO orbitals involved which are of 4f nature in the case of Ce.

Transition Number	Transition Wavelength	Contribution (%)
15	643.4	32.1
2	658.1	22.3
5	656.6	16.5
6	651.3	9.8
17	623.9	7.6

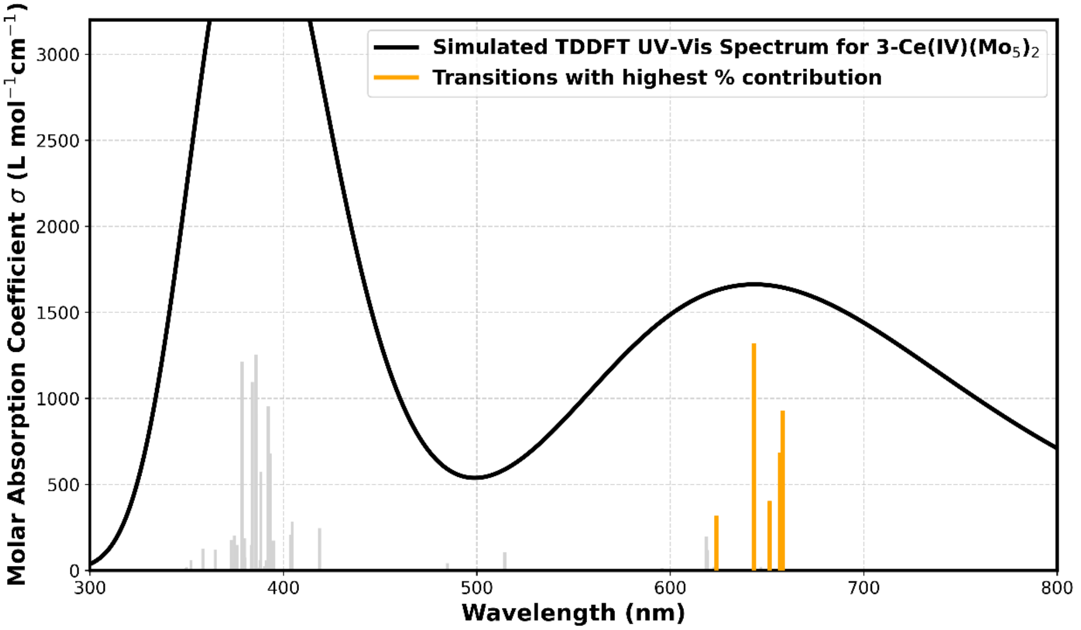

Figure S29. Simulated TDDFT UV-Vis spectrum for **3-Ce(Mo$_5$)$_2$**, highlighting the five most important transitions contributing to the band around 640nm. The orbitals involved in a representative transition (15) are shown in the following figures.

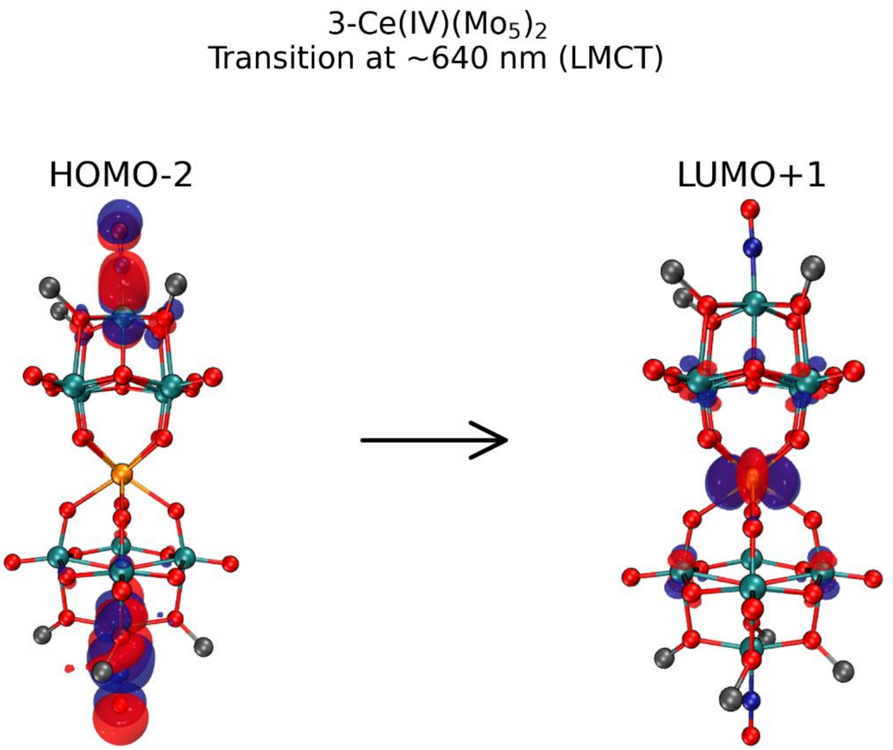

Figure S30. Representative molecular orbitals involved in the transition around 640nm for **3-Ce(Mo$_5$)$_2$**. This is the dominant contribution to the absorption band in this region (Figure S7), and it involves transfer from orbitals localized on the {Mo-NO}$_4$ region to the unoccupied 4f orbitals on Ce.

Table S9. The five most significant electronic transitions below 400 nm, obtained with TDDFT for **3-Ce(W$_4$Mo)$_2$**. These transitions are the ones contributing to the broad absorption band observed below 400 nm in the experimental UV-Vis spectrum. Differently from **3-Ce(Mo$_5$)$_2$**, there are two types of LMCT transitions. The dominant one, around 340-350 nm (transitions 88, 80, 82), is a LMCT from orbitals delocalized across the whole ligand to unoccupied 4f orbitals localized on Ce. The other, less important type, around 365 nm (transitions 44, 59), is similar to the LMCT of the molybdenum analogue, involving orbitals localized on the POM cage transferring to the unoccupied 4f orbitals.

**Transition Number**	**Transition Wavelength**	**Contribution (%)**
88	339.8	30.4
44	370.1	11.1
80	352.1	10.9
59	362.4	8.5
82	351.5	6.1

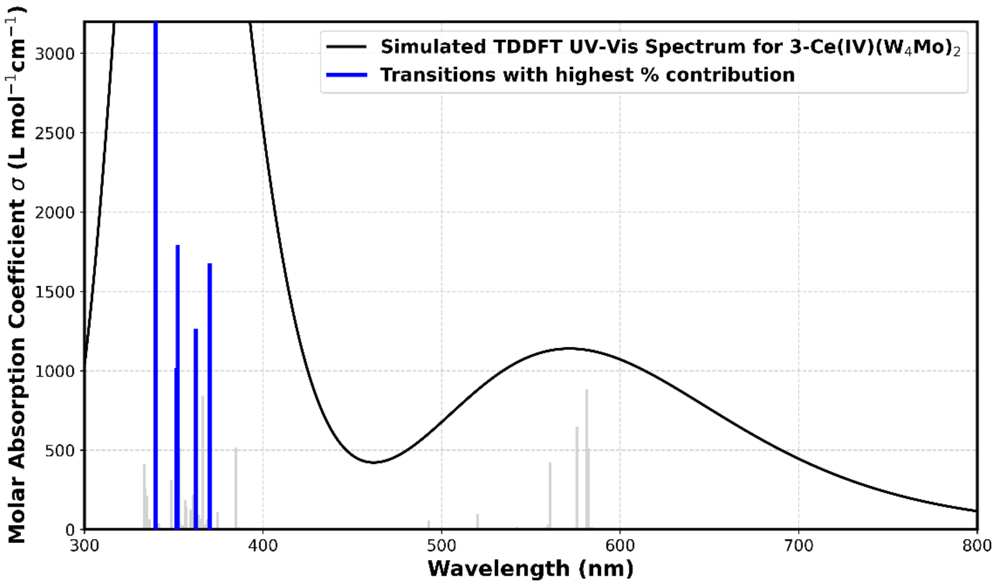

Figure S31. Simulated TDDFT UV-Vis spectrum for **3-Ce(W_4Mo)$_2$**, highlighting the five most important transitions contributing to the broad band below 400 nm. The orbitals involved in two representative transitions (88 and 44) are shown in the following figures.

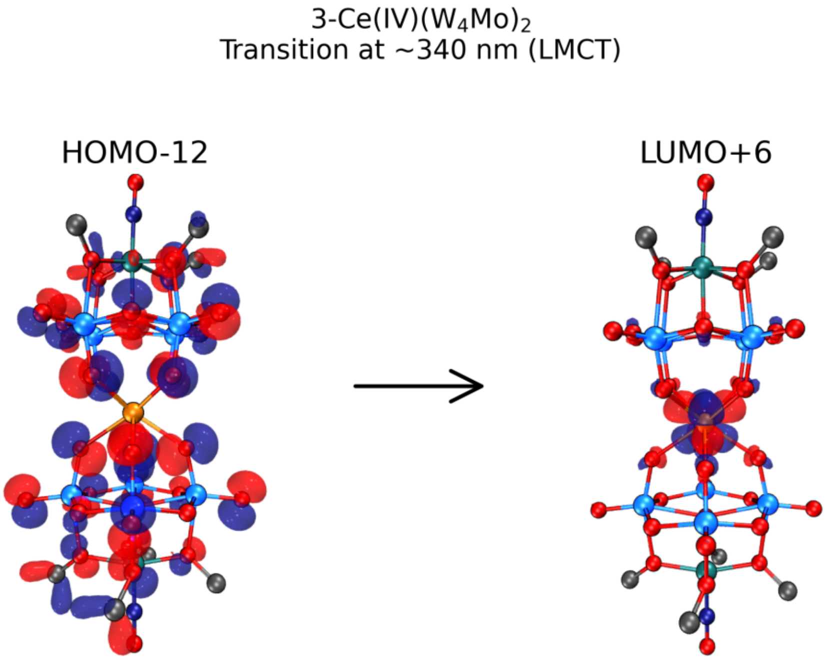

Figure S32. Representative molecular orbitals for the dominant LMCT transition in **3-Ce(W_4Mo)$_2$**. This LMCT transition involves highly delocalized orbitals spread across the equatorial plane of the POM ligand to the unoccupied 4f orbitals on the Ce center.

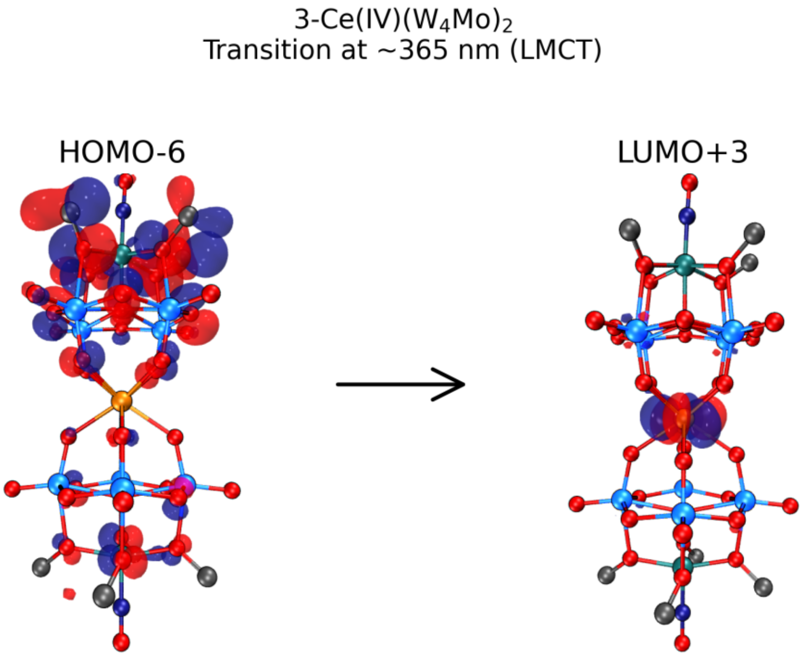

Figure S33. Representative molecular orbitals for the secondary type of **LMCT** transition in **3-Ce(W_4Mo)$_2$**. This process is analogous to that observed in the **3-Ce(Mo_5)$_2$** system, involving electron transfer from orbitals more localized on the POM cage to the unoccupied Ce 4f orbitals.

Table S10. The five most significant electronic transitions around 640nm, obtained with TDDFT for **3-Ce(W_4Mo)$_2$**. These transitions are the ones contributing to the absorption band observed around 640nm in the experimental UV-Vis spectrum. All these transitions involve a LMCT process from orbitals localized on the {Mo-NO}$_4$ to unoccupied 4f orbitals localized on Ce. This type of transition is consistent with our previous paper, with the difference in the LUMO orbitals involved which are of 4f nature in the case of Ce.

Transition Number	Transition Wavelength	Contribution (%)
13	581.4	31.1
15	575.7	23.0
11	582.2	17.9
20	560.7	14.8
30	520.0	2.6

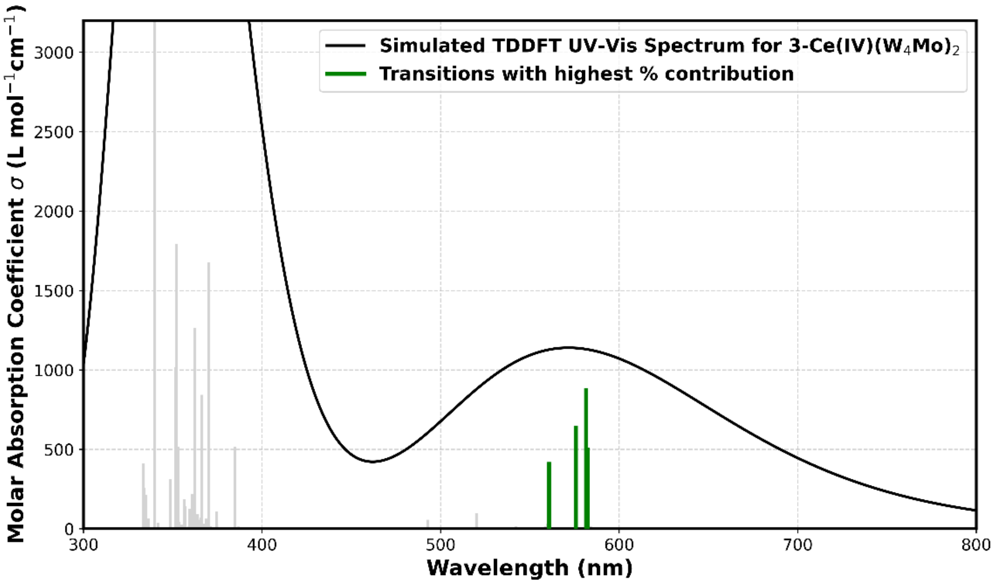

Figure S34. Simulated TDDFT UV-Vis spectrum for **3-Ce(W$_4$Mo)$_2$**, highlighting the five most important transitions contributing to the broad band below 400 nm. The orbitals involved in a representative transition (13) are shown in the following figures.

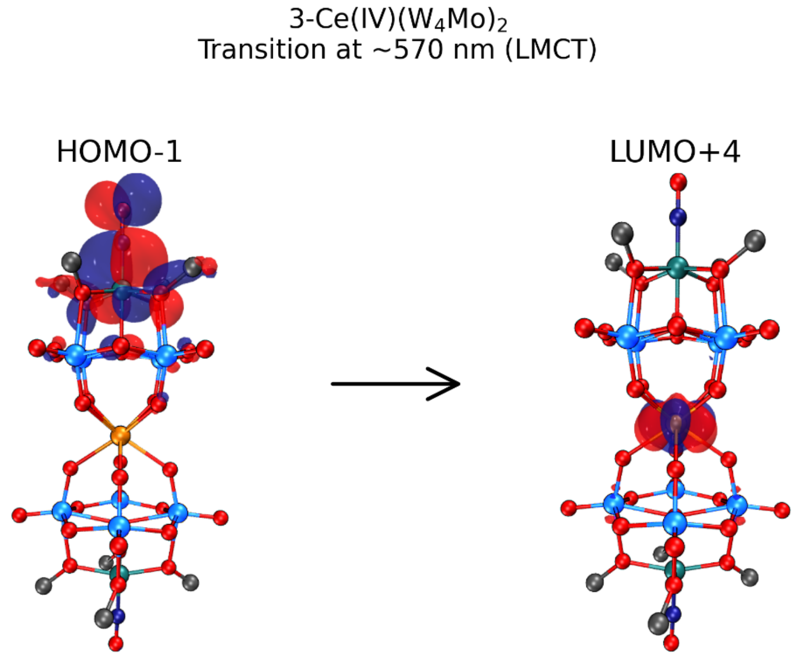

Figure S35. Representative molecular orbitals involved in the transition around 570nm for **3-Ce(W$_4$Mo)$_2$**. This is the dominant contribution to the absorption band in this region (Figure S8), and it involves transfer from orbitals localized on the {Mo-NO}$_4$ region to the unoccupied 4f orbitals on Ce. This is consistent with **3-Ce(Mo$_5$)$_2$** with a noticeable redshift observed for the latter, consistent with the experimental spectrum.

Table S11. Comparison of experimental and DFT calculated bond lengths (Å) for **3-Ce(Mo$_5$)$_2$**. The performance of two density functionals, PBE0 and B3LYP, is reported; combined with three distinct basis sets. The computational setups include: def2-TZVPP for O,N,C,H atoms and a Stuttgart pseudopotential and basis set for Ce and Mo; 6-311G(2d,2p) for O,N,C,H atoms and a Stuttgart pseudopotential and basis set for Ce and Mo; all electron ZORA/SARC-def2-TZVP basis for all atoms. The percentage error relative to the experimental values is shown in parentheses.

Bond Type	Exp.	PBE0/def2-TZVPP	B3LYP/def2-TZVPP	PBE0/6311G(2d,2p)	B3LYP/6311G(2d,2p)	PBE0-ZORA/SARC-def2-TZVP	B3LYP-ZORA/SARC-def2-TZVP
**Ce(IV)-O**	**2.3419**	2.3575 (0.67%)	2.3878 (1.96%)	2.3594 (0.75%)	2.3901 (2.06%)	2.3539 (0.51%)	2.3819 (1.71%)
**Mo(VI)-O**	**1.7739**	1.7503(-1.33%)	1.7641(-0.55%)	1.7513(-1.28%)	1.7652(-0.49%)	1.7503(-1.33%)	1.7653(-0.48%)

Table S12. NBO analysis of the σ-type NLMOs for the Ce-O bonds. NBO results for the **3-Ce(Mo$_5$)$_2$** system, focusing on the σ-type Natural Localized Molecular Orbitals (NLMOs) that constitute the Ce-O bonds. The average value obtained for the eight equivalent Ce-O-Mo bridges is presented, and this was obtained using various DFT functional/basis set combinations as listed in the first column. The remaining columns show the average percentage contributions of the O, Ce, and Mo atoms to the NLMO, along with the corresponding average orbital character for each atomic contribution.

Functional/Basis Set	O			Ce				Mo		
	% O	s	p	%Ce	s	d	f	%Mo	s	d
B3LYP/def2-TZVPP	90.81	60.20	39.78	7.62	11.73	46.77	41.40	1.07	18.47	80.57
PBE0/def2-TZVPP	90.51	59.53	40.44	7.96	11.24	48.49	40.17	1.02	20.14	78.79
B3LYP/6311G(2d,2p)	91.34	60.73	39.24	7.25	13.90	46.46	39.50	0.98	17.50	80.73
PBE0/6311G(2d,2p)	90.96	60.32	39.65	7.63	13.21	47.98	38.69	0.96	18.76	79.33
B3LYP/SARC-def2-TZVP	90.81	60.20	39.78	7.62	11.73	46.77	41.40	1.07	18.47	80.57
PBE0/SARC-def2-TZVP	90.69	64.35	35.64	8.00	12.52	55.78	31.57	0.86	28.14	70.58

Table S13. NBO analysis of the π-type NLMOs for the Ce-O bonds. NBO results for the **3-Ce(Mo$_5$)$_2$** system, focusing on the π-type Natural Localized Molecular Orbitals (NLMOs) that constitute the Ce-O bonds. The average value obtained for the eight equivalent Ce-O-Mo bridges is presented, and this was obtained using various DFT functional/basis set combinations as listed in the first column. The remaining columns show the average percentage contributions of the O, Ce, and Mo atoms to the NLMO, along with the corresponding average orbital character for each atomic contribution.

Functional/Basis Set	O			Ce				Mo	
	% O	s	p	%Ce	s	d	f	%Mo	d
B3LYP/def2-TZVPP	81.55	1.35	98.55	3.73	2.25	31.48	65.83	10.53	98.63
PBE0/def2-TZVPP	81.55	2.02	97.88	3.93	2.81	34.57	62.15	10.30	98.39
B3LYP/6311G(2d,2p)	81.81	1.65	98.26	3.77	2.56	33.07	64.10	10.29	98.52
PBE0/6311G(2d,2p)	81.67	2.18	97.73	3.88	2.96	35.68	61.14	10.32	98.22
B3LYP/SARC-def2-TZVP	81.55	1.35	98.55	3.73	2.25	31.48	65.83	10.53	98.63
PBE0/SARC-def2-TZVP	81.72	2.16	97.71	3.44	3.97	44.74	50.98	10.44	98.74

Table S14. NBO analysis of the π-type NLMOs for the Mo-O bonds. NBO results for the **3-Ce(Mo$_5$)$_2$** system, focusing on the π-type Natural Localized Molecular Orbitals (NLMOs) that constitute the Mo-O bonds within the bridging units. The average value obtained for the eight equivalent Ce-O-Mo bridges is presented, and this was obtained using various DFT functional/basis set combinations as listed in the first column. The remaining columns show the average percentage contributions of the O, Ce, and Mo atoms to the NLMO, along with the corresponding average orbital character for each atomic contribution.

Functional/Basis Set	O		Ce			Mo	
	% O	p	%Ce	d	f	%Mo	d
B3LYP/def2-TZVPP	75.72	99.75	2.15	28.95	70.67	20.82	99.41
PBE0/def2-TZVPP	75.69	99.88	2.20	30.78	68.85	20.33	99.35
B3LYP/6311G(2d,2p)	75.33	99.88	2.37	33.11	66.66	19.04	99.33
PBE0/6311G(2d,2p)	75.67	99.89	2.44	34.92	64.86	17.15	99.25
B3LYP/SARC-def2-TZVP	75.72	99.75	2.15	28.95	70.67	20.82	99.41
PBE0/SARC-def2-TZVP	75.50	99.87	2.15	39.19	60.22	20.46	99.62

Table S15. NBO analysis of the σ/ π -type NLMOs for the Mo-O bonds. NBO results for the **3-Ce(Mo$_5$)$_2$** system, focusing on the σ-type Natural Localized Molecular Orbitals (NLMOs) that constitute the Mo-O bonds within the bridging units. The average value obtained for the eight equivalent Ce-O-Mo bridges is presented, and this was obtained using various DFT functional/basis set combinations as listed in the first column. The remaining columns show the average percentage contributions of the O, Ce, and Mo atoms to the NLMO, along with the corresponding average orbital character for each atomic contribution.

**Functional/Basis Set**	**O**			**Ce**			**Mo**		
	**% O**	**s**	**p**	**%Ce**	**d**	**f**	**%Mo**	**s**	**d**
B3LYP/def2-TZVPP	77.74	40.43	59.46	0.65	31.14	66.51	20.92	10.87	88.61
PBE0/def2-TZVPP	77.80	40.09	59.79	0.67	31.33	66.23	20.82	11.07	88.39
B3LYP/6311G(2d,2p)	78.23	39.20	60.70	0.68	30.41	68.24	20.43	12.58	86.63
PBE0/6311G(2d,2p)	78.29	38.68	61.21	0.69	29.94	68.64	20.34	12.70	86.45
B3LYP/SARC-def2-TZVP	77.74	40.43	59.46	0.65	31.14	66.51	20.92	10.87	88.61
PBE0/SARC-def2-TZVP	77.54	33.75	66.12	0.90	30.74	67.52	20.85	9.90	89.62

Table S16. Average DI values for the M(IV)-O-M(VI) bridges bonds for the four different central metal atoms respectively for **[M(IV){M'$_4$O$_{13}$(OMe)$_4$MoNO}$_2$]** (where M(IV) = Zr, Hf, Ce, Th and M' = Mo or W) systems. The second column shows the DI values for the M(IV)-O bonds in the Mo analogues, while third column shows the DI values for the tungsten ones. Fourth and fifth column show the DI values for the M(VI)-O bonds respectively for the Mo and W systems.

	**[M(IV){Mo$_4$O$_{13}$(OMe)$_4$MoNO}$_2$]**		**[M(IV){W$_4$O$_{13}$(OMe)$_4$MoNO}$_2$]**	
	M(IV)-O	Mo(VI)-O	M(IV)-O	W(VI)-O
Zr	0,3192	1,3066	0,3174	1,2588
Hf	0,3161	1,3058	0,3133	1,2592
Ce	0,3839	1,3141	0,3831	1,2635
Th	0,3498	1,3172	0,3482	1,2667

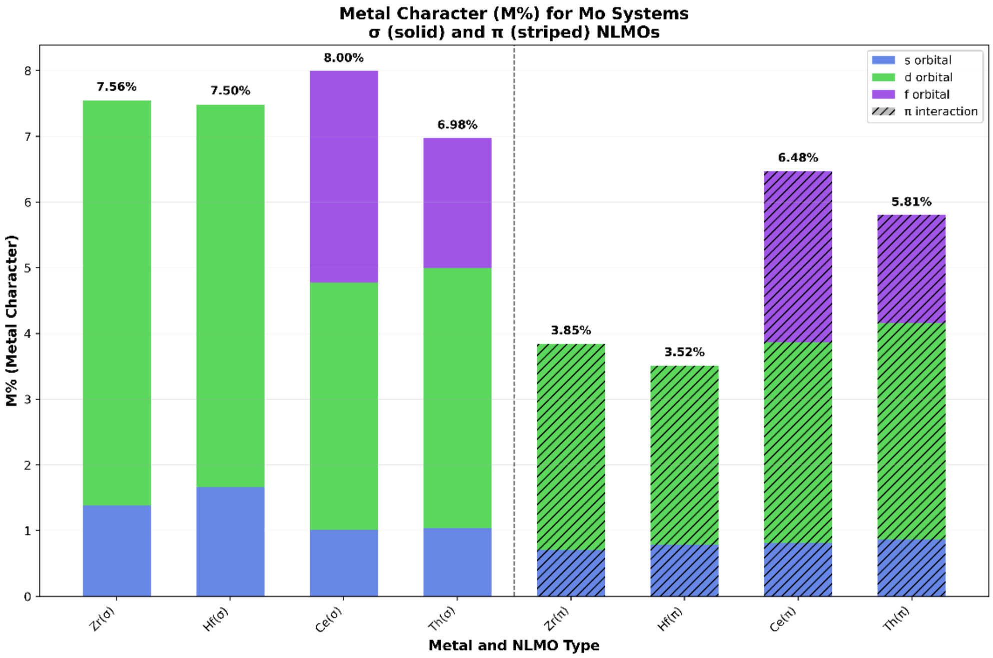

Figure S36. Breakdown of the central metal atom contribution to M(IV)−O bonding in the **[M(IV){$Mo_4O_{13}(OMe)_4MoNO$}$_2$]** (where M(IV) = Zr, Hf, Ce, Th) systems. The bar plots shows the average percentage contribution from the central M(IV) atom to the M(IV)−O Natural Localized Molecular Orbitals (NLMOs). The contributions are separated into their σ and π components, with the specific character of the contributing metal orbitals also detailed.

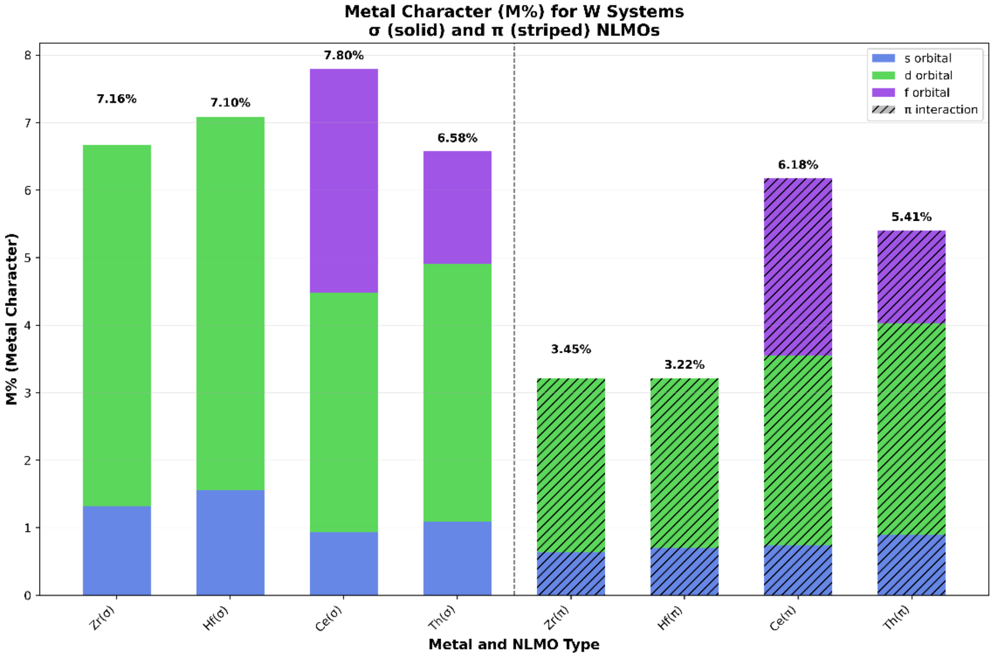

Figure S37. Breakdown of the central metal atom contribution to M(IV)−O bonding in the **[M(IV){$W_4O_{13}(OMe)_4MoNO$}$_2$]** (where M(IV) = Zr, Hf, Ce, Th) systems. The bar plot shows the average percentage contribution from the central M(IV) atom to the M(IV)−O Natural Localized Molecular Orbitals (NLMOs). The contributions are separated into their σ and π components, and the specific character of the contributing metal orbitals is detailed.

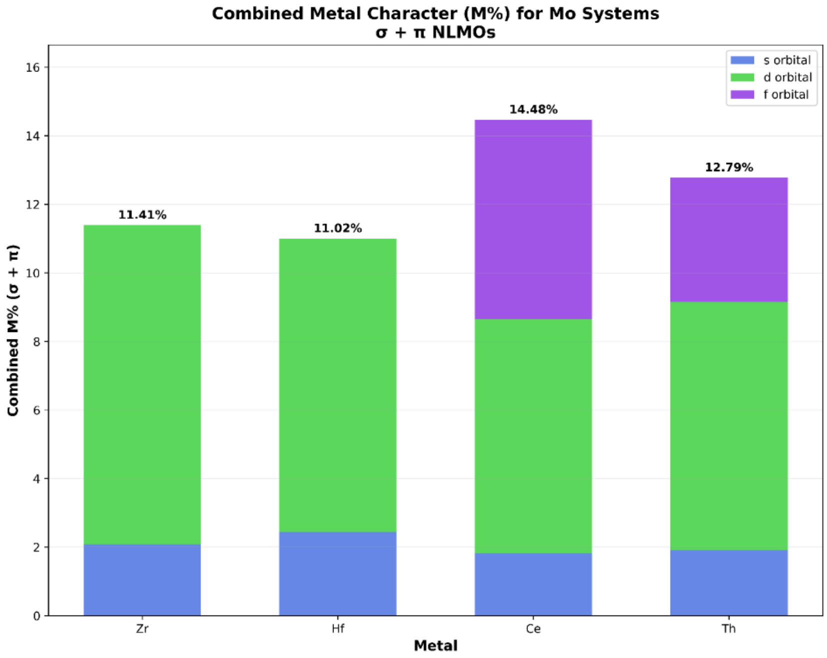

Figure S38. Total central M(IV) atom contribution to M(IV)−O bonding in the **[M(IV){$Mo_4O_{13}(OMe)_4$MoNO}$_2$]** systems. This bar plot summarizes the combined average (σ + π) percentage contribution from the central M(IV) atom to the M(IV)−O NLMOs. The overall character of the contributing metal orbitals is also shown for each system.

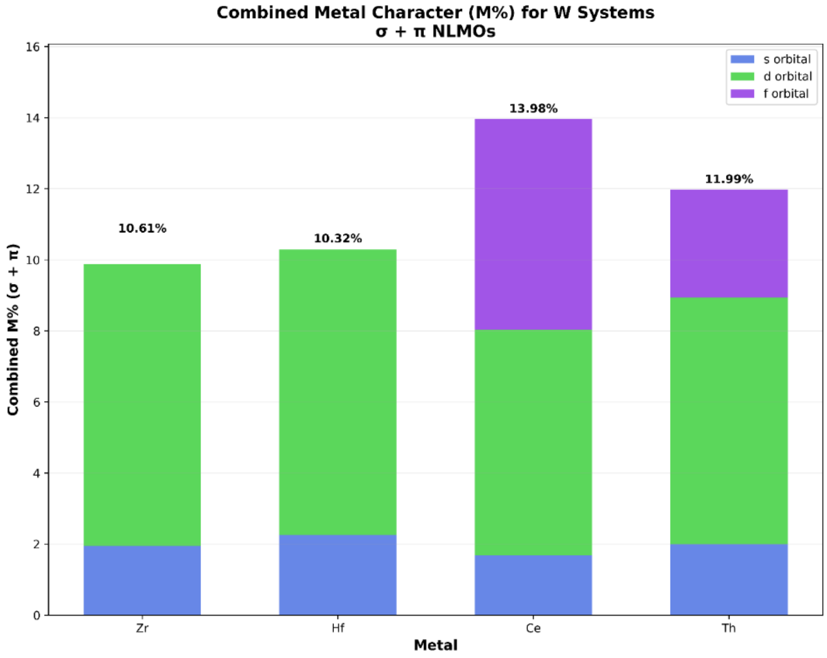

Figure S39. Total central M(IV) atom contribution to M(IV)−O bonding in the **[M(IV){$W_4O_{13}(OMe)_4$MoNO}$_2$]** systems. This bar plot summarizes the combined average (σ + π) percentage contribution from the central M(IV) atom to the M(IV)−O NLMOs. The overall character of the contributing metal orbitals is presented for each system.

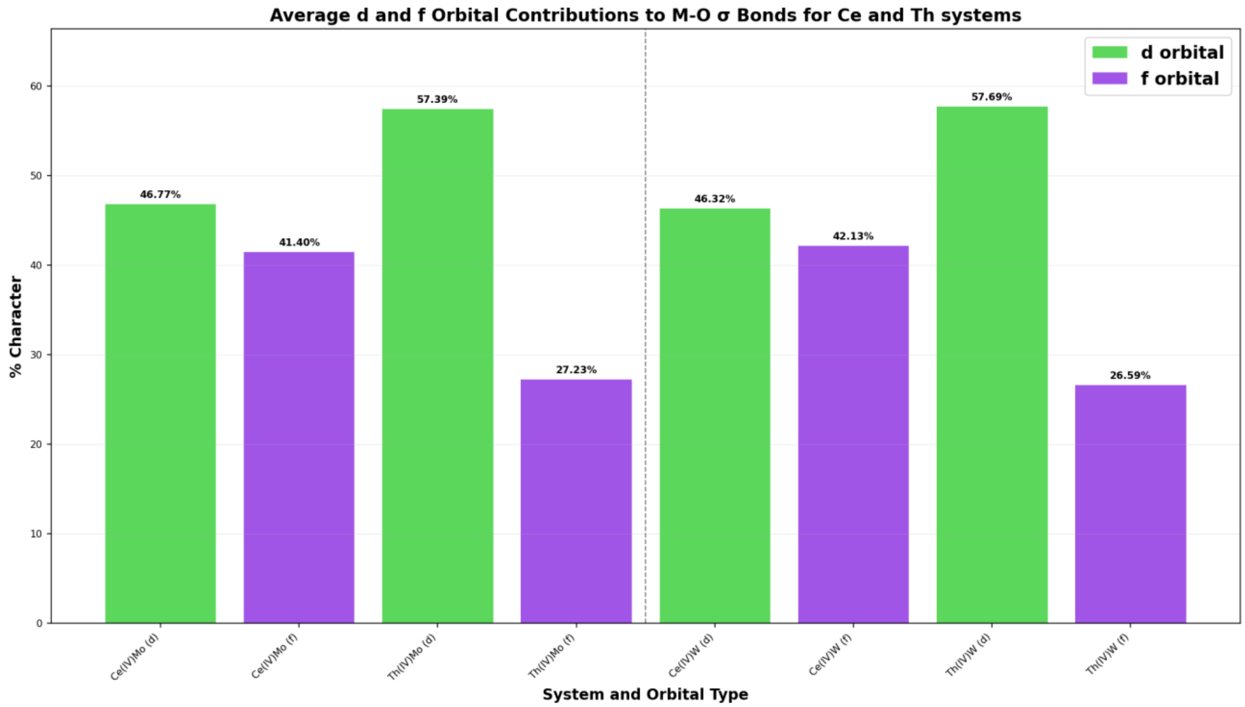

Figure S40. Bar plot highlighting the average d and f orbital character of the σ-type NLMOs describing the M(IV)-O bonds.

Table S17. Average d and f orbital character of the σ-type NLMOs describing the M(IV)-O bonds in **3-Ce(Mo_5)$_2$** and **3-Ce(W_4Mo)$_2$** and **(TBA)$_2$[Th{M'$_4$O$_{13}$(OMe)$_4$MoNO}$_2$]** (where M' = Mo or W)

	Avg. d-orbital Contribution (%)	Avg. f-orbital Contribution (%)
**Ce(IV)Mo**	**46.77**	**41.40**
**Th(IV)Mo**	**57.39**	**27.23**
**Ce(IV)W**	**46.32**	**42.13**
**Th(IV)W**	**57.69**	**26.59**

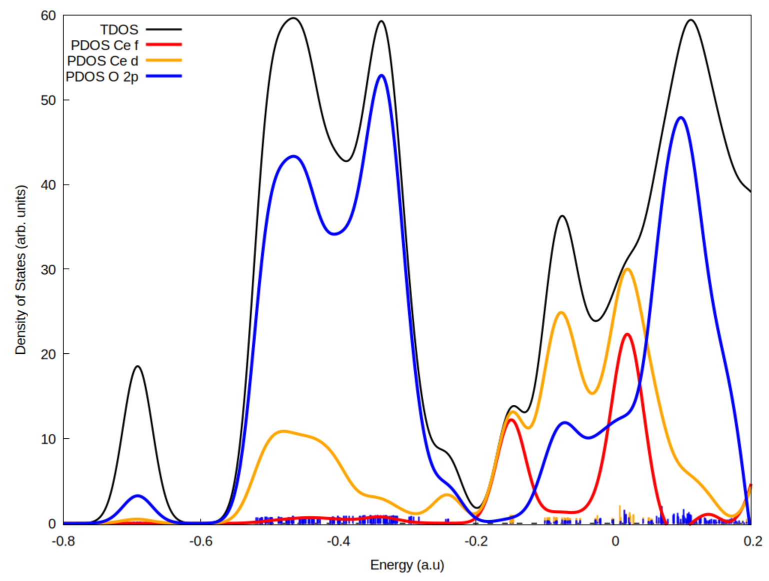

Figure S41. Calculated DOS for **3-Ce(Mo$_5$)$_2$**. The black curve shows the total DOS (TDOS). The blue curve shows the projected DOS (PDOS) of the 2p orbitals of the O atoms involved in Ce(IV)-O bonds. The orange curve shows the PDOS for Ce 5d orbitals, and the red curve shows the vacant Ce 4f orbitals (left peak) and 5f (right peak). The presence of lower energy 4f orbitals is what dictates the better energy matching with the O 2p orbitals.

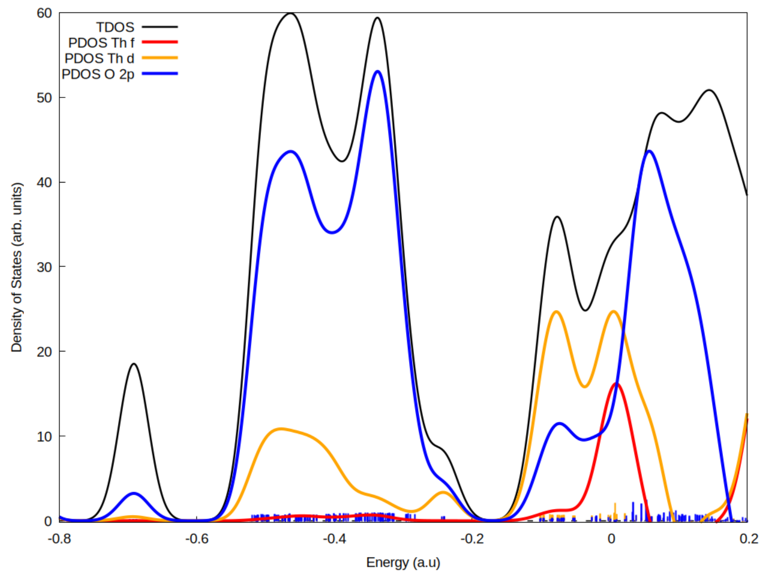

Figure S42. Calculated DOS for **(TBA)$_2$[Th{Mo$_4$O$_{13}$(OMe)$_4$MoNO}$_2$]**. The black curve shows the total DOS (TDOS). The blue curve shows the projected DOS (PDOS) of the 2p orbitals of the O atoms involved in Th(IV)-O bonds. The orange curve shows the PDOS for Th 6d orbitals, and the red curve shows the vacant 5f orbitals. The presence of lower energy 6d orbitals can be connected to the higher d orbital character in the corresponding NLMOs.

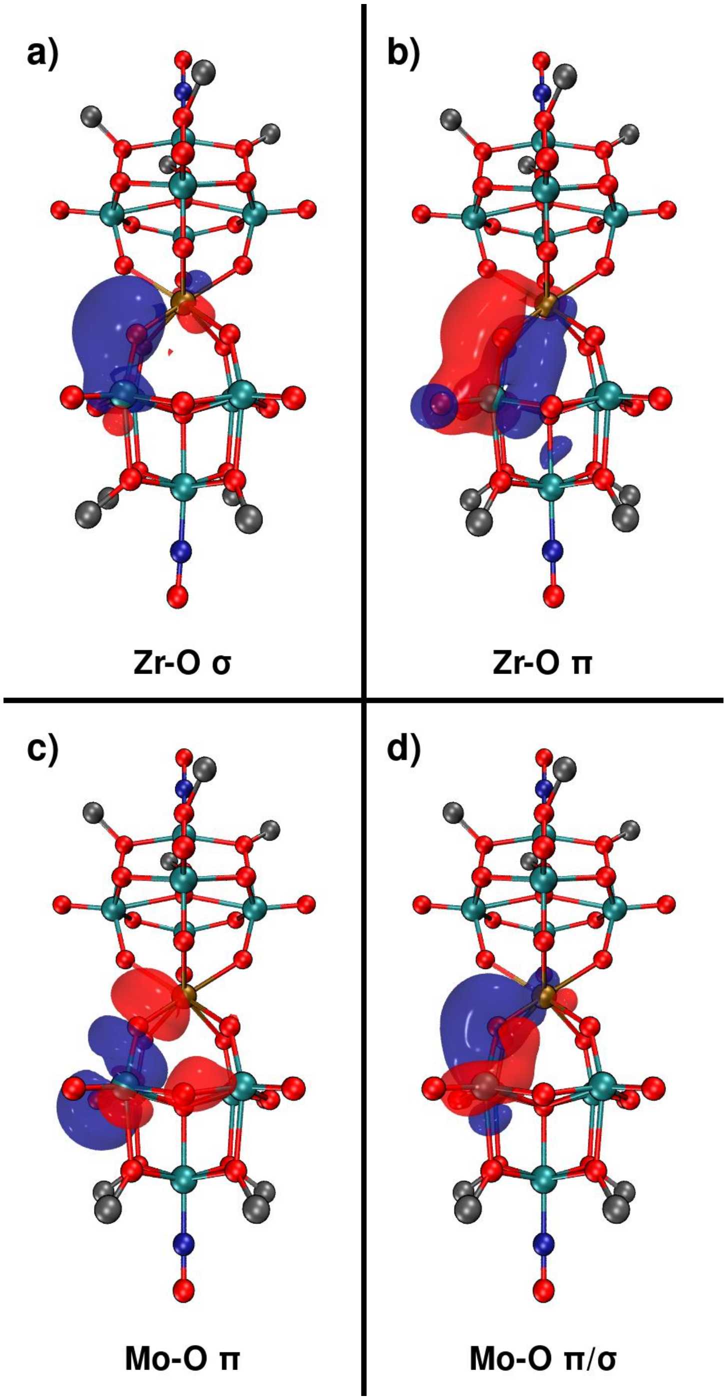

Figure S43. Isosurface plots of the main NLMOs for the Total central M(IV) atom contribution to M(IV)−O bonding in the $[Zr(IV)\{Mo_4O_{13}(OMe)_4MoNO\}_2]$ system. Isosurface plots (rendered at 0.23 a.u.) of the four representative NLMOs involved in the Zr-O-Mo bridging bonds. The panel shows: **a)** the σ-type NLMO of the Zr−O bond; **b)** the π-type NLMO of the Zr−O bond; **c)** the primary π-type NLMO of the Mo−O bond; and **d)** the the σ /π-type NLMO of the Mo−O bond.

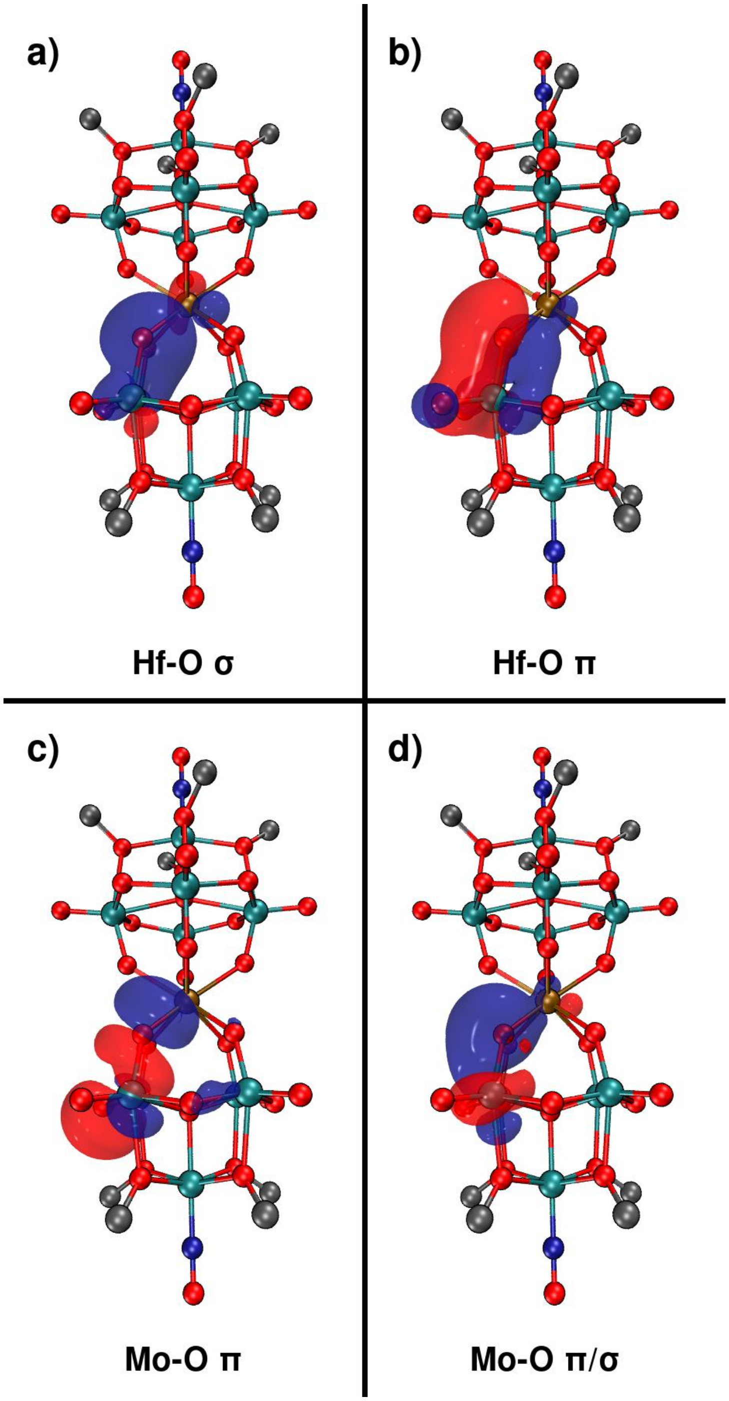

Figure S44. Isosurface plots of the main NLMOs for the Total central M(IV) atom contribution to M(IV)−O bonding in the [Hf(IV){$Mo_4O_{13}(OMe)_4MoNO$}$_2$] system. Isosurface plots (rendered at 0.23 a.u.) of the four representative NLMOs involved in the Hf-O-Mo bridging bonds. The panel shows: a) the σ-type NLMO of the Hf−O bond; b) the π-type NLMO of the Hf−O bond; c) the primary π-type NLMO of the Mo−O bond; and d) the σ/π-type NLMO of the Mo−O bond.

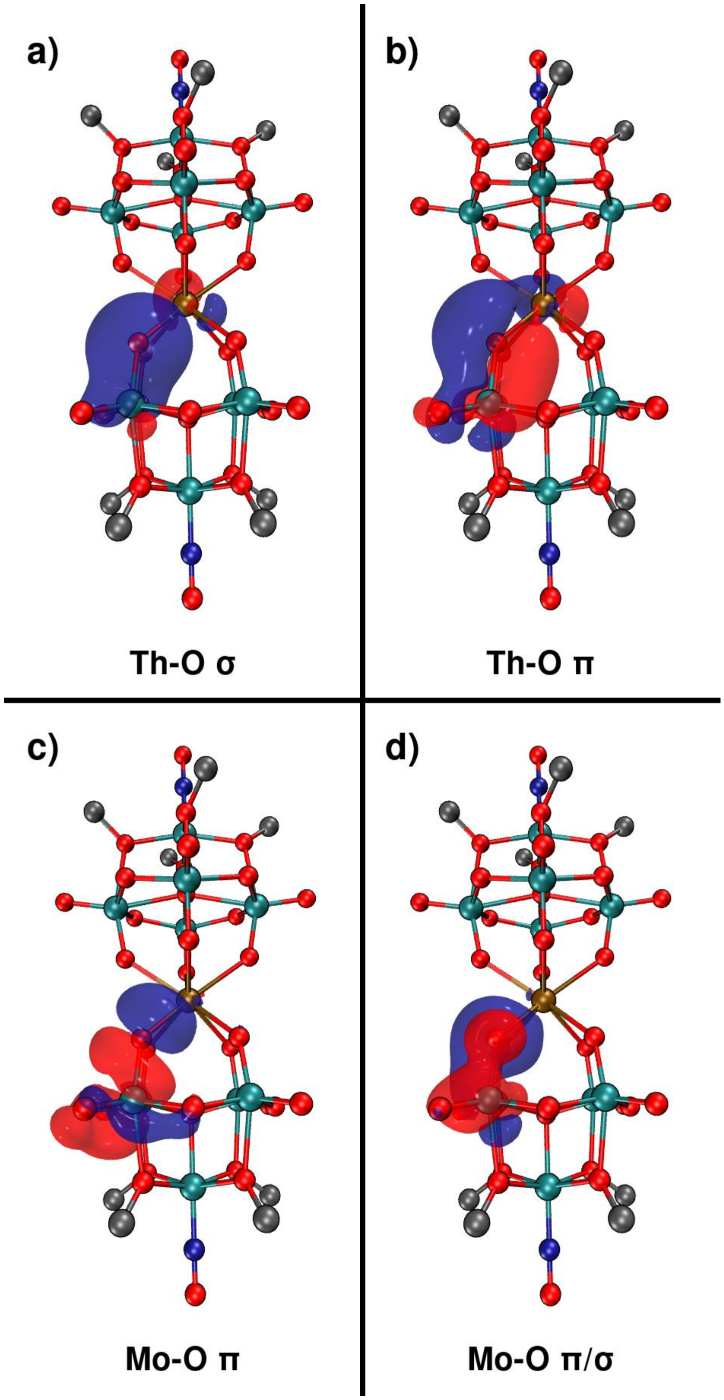

Figure S45. Isosurface plots of the main NLMOs for the Total central M(IV) atom contribution to M(IV)−O bonding in the [Th(IV){$Mo_4O_{13}(OMe)_4MoNO$}$_2$] system. Isosurface plots (rendered at 0.23 a.u.) of the four representative NLMOs involved in the Th-O-Mo bridging bonds. The panel shows: a) the σ-type NLMO of the Th−O bond; b) the π-type NLMO of the Th−O bond; c) the primary π-type NLMO of the Mo−O bond; and d) the σ/π-type NLMO of the Mo−O bond.

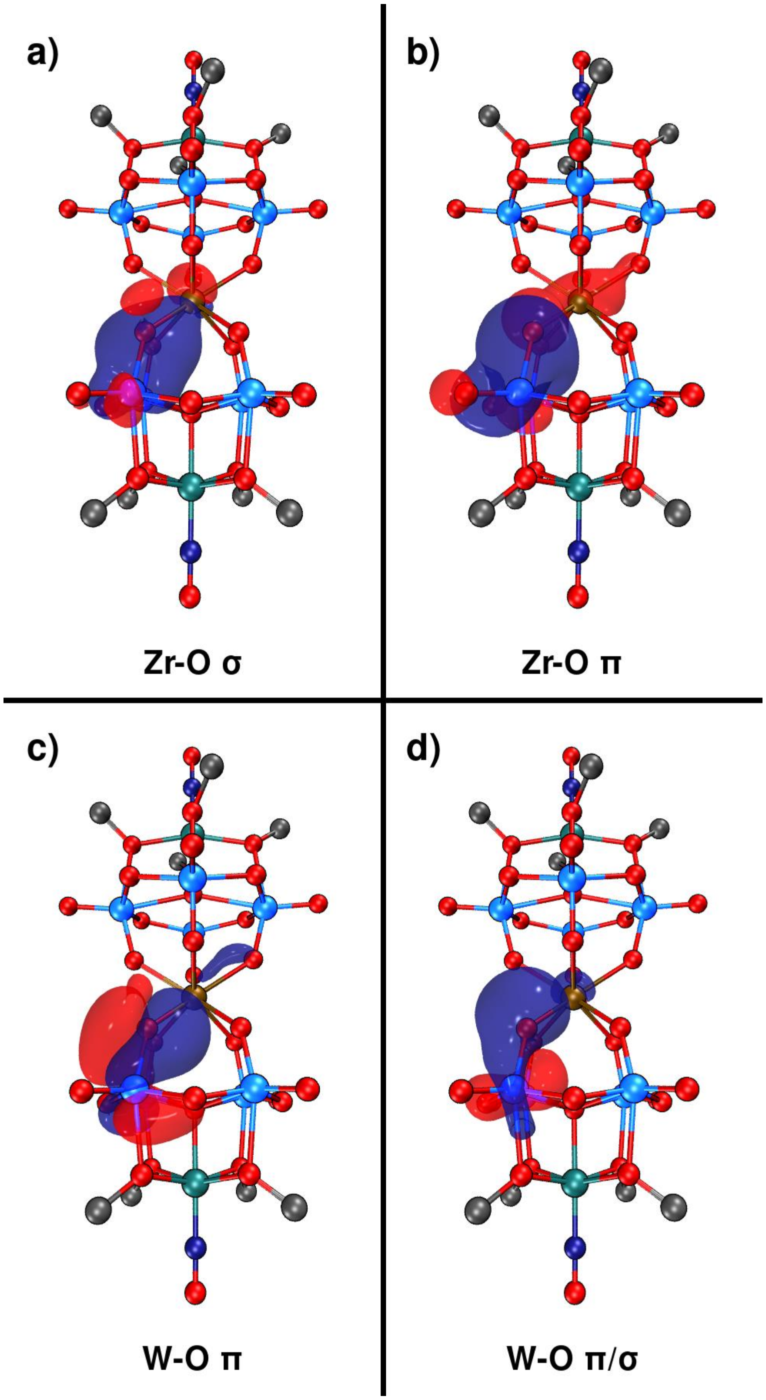

Figure S46. Isosurface plots of the main NLMOs for the Total central M(IV) atom contribution to M(IV)−O bonding in the $[Zr(IV)\{W_4O_{13}(OMe)_4MoNO\}_2]$ system. Isosurface plots (rendered at 0.23 a.u.) of the four representative NLMOs involved in the Zr-O-W bridging bonds. The panel shows: a) the σ-type NLMO of the Zr−O bond; b) the π-type NLMO of the Zr−O bond; c) the primary π-type NLMO of the W−O bond; and d) the σ/π-type NLMO of the W−O bond.

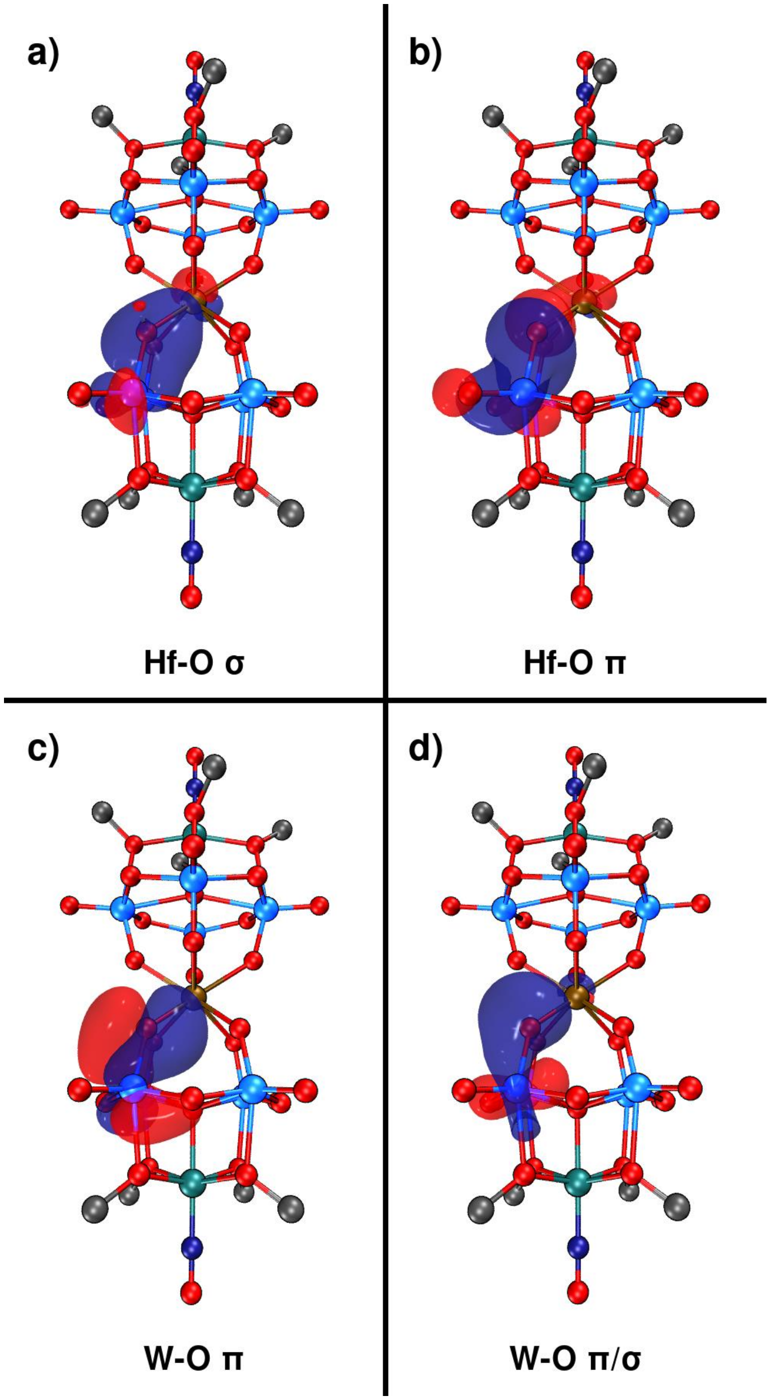

Figure S47. Isosurface plots of the main NLMOs for the Total central M(IV) atom contribution to M(IV)−O bonding in the [Hf(IV){$W_4O_{13}(OMe)_4MoNO$}$_2$] system. Isosurface plots (rendered at 0.23 a.u.) of the four representative NLMOs involved in the Hf-O-W bridging bonds. The panel shows: a) the σ-type NLMO of the Hf−O bond; b) the π-type NLMO of the Hf−O bond; c) the primary π-type NLMO of the W−O bond; and d) the σ/π-type NLMO of the W−O bond.

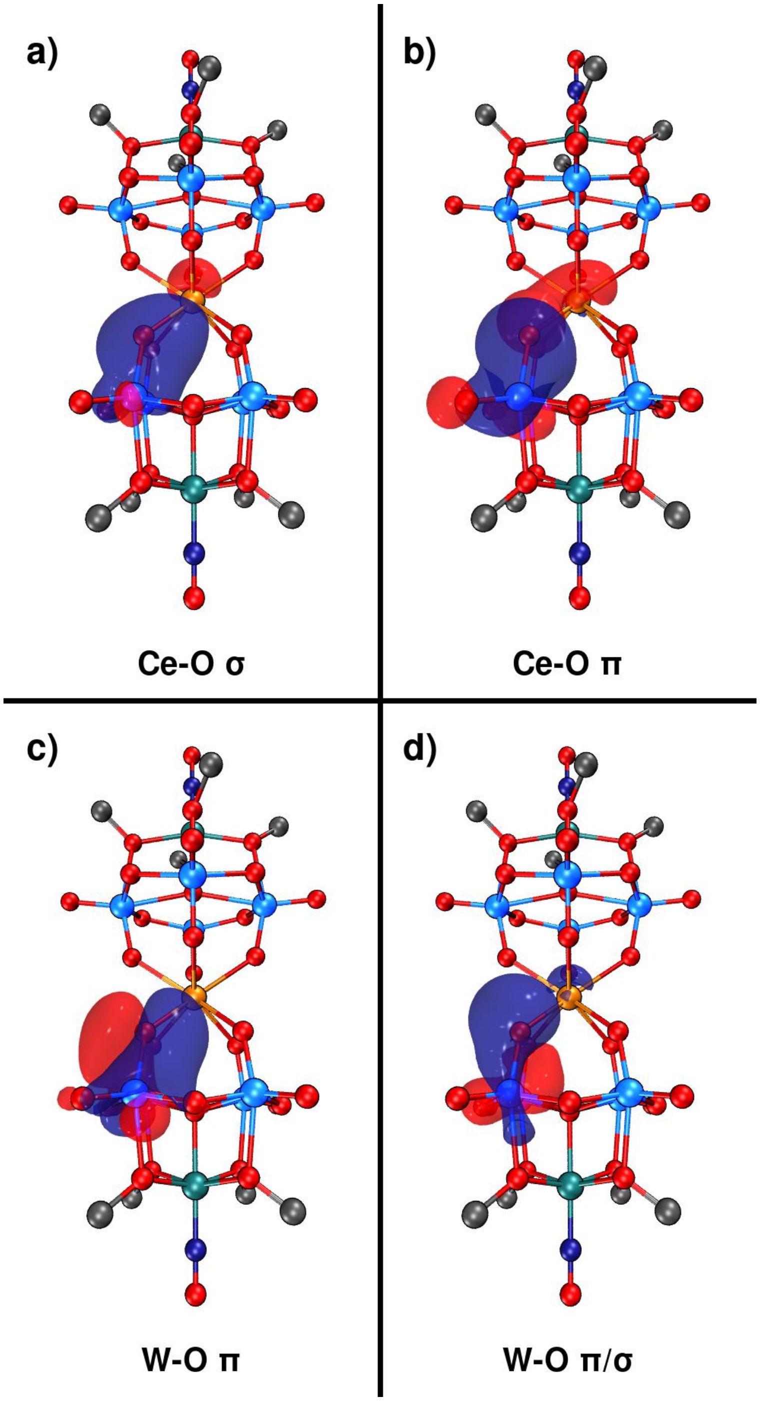

Figure S48. Isosurface plots of the main NLMOs for the Total central M(IV) atom contribution to M(IV)−O bonding in the [Ce(IV){$MW_4O_{13}(OMe)_4MoNO$}$_2$] system. Isosurface plots (rendered at 0.23 a.u.) of the four representative NLMOs involved in the Ce-O-W bridging bonds. The panel shows: a) the σ-type NLMO of the Ce−O bond; b) the π-type NLMO of the Ce−O bond; c) the primary π-type NLMO of the W−O bond; and d) the σ/π-type NLMO of the W−O bond.

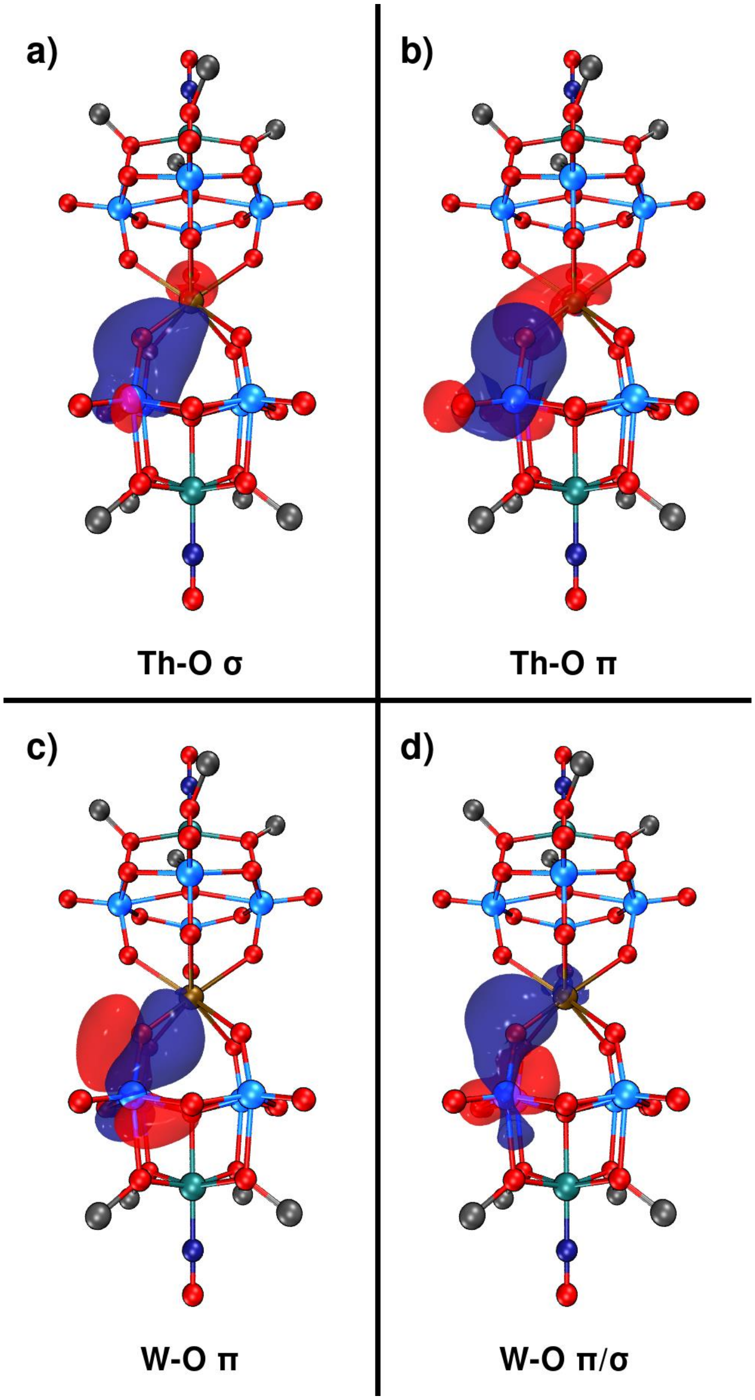

Figure S49. Isosurface plots of the main NLMOs for the Total central M(IV) atom contribution to M(IV)−O bonding in the [Th(IV){$W_4O_{13}(OMe)_4MoNO$}$_2$] system. Isosurface plots (rendered at 0.23 a.u.) of the four representative NLMOs involved in the Th-O-W bridging bonds. The panel shows: a) the σ-type NLMO of the Th−O bond; b) the π-type NLMO of the Th−O bond; c) the primary π-type NLMO of the W−O bond; and d) the σ/π-type NLMO of the W−O bond.

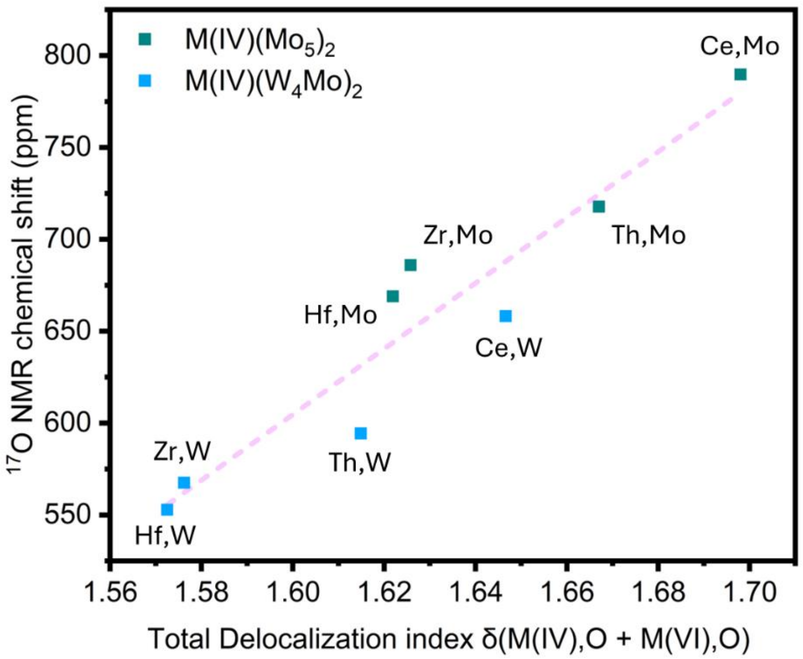

Figure S50. Plot of the sum of the average M(IV)-O and M(VI)-O delocalization indices obtained from QTAIM analysis vs. ^{17}O NMR chemical shift (ppm) for the complexes discussed in this work.
